# Probiotic strain *Bacillus subtilis* TO-A modulates the formation of neutrophil extracellular traps

**DOI:** 10.1080/29933935.2025.2572788

**Published:** 2025-10-29

**Authors:** Takuto Hayashi, Rena Nakagomi, Yoichi Okino, Satoshi Shimazaki, Diansheng Wang

**Affiliations:** Research Division, TOA Biopharma Co., Ltd., Tatebayashi-shi, Japan

**Keywords:** Probiotics, neutrophil extracellular traps, NET formation, *Bacillus subtilis* TO-A, HL-60 cell line, neutrophil-like cells, intestinal bacterial infection, inflammatory bowel disease

## Abstract

Neutrophil extracellular traps (NETs) have important innate immune functions that prevent pathogenic infections. In the intestinal tract, NET formation contributes to the prevention of the systemic spread of commensal bacteria associated with mucosal barrier dysfunction, and exacerbates inflammation in various chronic enteritis conditions. Therefore, NETs in the intestinal tract are bilateral and appropriate control methods based on the host's condition are required. In this study, we found that the probiotic bacterial strain *Bacillus subtilis* TO-A (BSTOA), used in both human and veterinary medicine, modulates NET formation in a bidirectional manner. Co-culture of neutrophil-like HL-60 (nHL-60) cells with BSTOA at a low ratio of 0.1:1 (BSTOA:nHL-60 cells) promoted subsequent NET formation. Conversely, co-culture of nHL-60 cells with BSTOA at a high ratio of 10:1 suppressed NET formation. Furthermore, transcriptome analysis demonstrated that BSTOA affects cellular processes through pathways such as peptidyl-prolyl isomerization, regulation of cyclin-dependent protein serine/threonine kinase activity, and chromatin remodeling to exert its function in nHL-60 cells. This is the first report of a single bacterial strain with the ability to regulate NET formation in a bidirectional manner, suggesting that BSTOA has potential utility as a preventive or therapeutic agent for NET-related diseases.

## Graphical abstract

**Figure:**
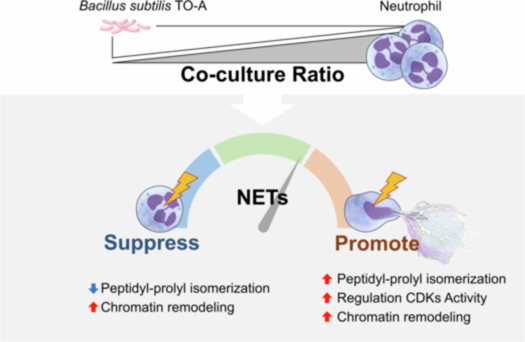

## Introduction

Neutrophils migrate from the peripheral blood to the infection site when pathogens invade the body, capturing the pathogens through phagocytosis or by forming neutrophil extracellular traps (NETs). They use granule enzymes, reactive oxygen species, and antimicrobial peptides, to lyse the pathogens thereby preventing their systemic spread.^1^^,^^2^ Thus, if pathogens suppress the formation of NETs or degrade them, their infectivity and ability to spread throughout the body would be high.^3^ Various pathogens such as gram-positive bacteria,^4-6^ gram-negative bacteria,^7-10^ mycobacteria,^11^ fungi,^6^^,^^12^ viruses,^13^ and protozoa,^14^ can induce neutrophils to form NETs, which are web-like structures comprising chromatin with granule proteins.^4^ NETs inhibit pathogens but can also harm host tissues owing to their lack of specificity.^15^ In addition, as chromatin is confined within the nucleus, its extracellular exposure owing to NET formation is believed to trigger autoantibody production.^16^ Hence, NET formation is crucial for defense against pathogens; however, they exacerbate various inflammatory diseases.

Pathogens invade the body through body surfaces or hollow organs, such as the digestive and respiratory tracts. The intestinal tract is a typical entrance for pathogens and home to a large number of commensal bacteria. Therefore, if the intestinal barrier comprising mucus layer and epithelial cells is destroyed, there is a risk that a large numbers of commensal bacteria and pathogens will infiltrate the tissues and bloodstream.^17^ Thus, in the intestine, NET formation is an essential immune mechanism to inhibit invading pathogens locally and prevent their systemic spread.^18^ Additionally, NETs are detected in the intestine of patients with non-steroidal anti-inflammatory drugs (NSAIDs)-induced small intestinal inflammation and inflammatory bowel diseases, wherein specific pathogenic bacteria have not been identified.^19-21^ Experimental studies show that these pathological conditions are ameliorated by the inhibition of NET formation via gene deletion, chemical reagent administration,^19^^,^^21^^,^^22^ or the degradation and removal of NETs by DNase I.^23^

In addition to pathogenic or commensal bacteria, consumption of beneficial bacteria can influence the intestinal function. Probiotics have been defined as ‘live microorganisms that when administered in adequate amounts confer a health benefit on the host.’^24^ Oral probiotic products, which are easy to administer, discontinue, and adjust the dosage, have been proposed to be useful for controlling NET formation in the gastrointestinal tract.^14^ However, the only probiotic strain reported to affect NET formation is *Lacticaseibacillus rhamnosus* GG (LGG).^25^

A traditional probiotic product containing three bacterial strains namely, *Bacillus subtilis* TO-A (BSTOA), *Enterococcus faecium* T-110 (EFT110), and *Clostridium butyricum* TO-A (CBTOA) has been used to treat intestinal dysbiosis in both human and veterinary medicine in Japan for more than half a century. These three strains are thought to engage in a symbiotic interaction that promotes the proliferation of each strain and produces each beneficial effects characteristic of respective species.^26^ Currently, its efficacy has been reported for the treatment of infectious diarrhea^27^ and gastroenteritis,^26^ prevention of *Clostridioides difficile* infections^28^ and surgical site infections in colorectal cancer,^29^ treatment and maintenance of remission in ulcerative colitis,^30^^,^^31^ and mitigation of gastrointestinal side effects during oxaliplatin therapy.^32^ Recent studies have also reported the beneficial potentials of each bacterium. For example, BSTOA extended the lifespan of *Caenorhabditis elegans*,^33^ while the CBTOA culture supernatant downregulated the expression of several inflammation- and DNA replication/repair-related genes in human cervical cancer HeLa S3 cells.^34^ Despite the strong implication of NETs in infections, ulcerative colitis, and oxaliplatin therapy,^20^^,^^21^^,^^35^ the relationship between these probiotic strains and NETs remains unexplored. Here, to the best of our knowledge, it is reported for the first time that when co-cultured with neutrophil-like cells, BSTOA exerts bidirectional effects on NET formation, with promoting and suppressive effects at low and high concentrations, respectively. These findings suggest the novel possibility that a single bacterial strain may regulate NET formation in both directions, offering a new perspective for controlling NET-related diseases using probiotics.

## Results

### BSTOA modulates NET formation in neutrophil-like cells with a co-culture ratio-dependent manner

To investigate whether the probiotic strains modulated NET formation, we used differentiated HL-60 cells (see Materials and Methods for the induction method) as neutrophil-like (nHL-60) cells. nHL-60 cells exhibited mature neutrophil characteristics, including growth arrest, lobulated nuclei, and phagocytic activity (Supplementary Figure S1a−c). nHL-60 cells also responded to the NET formation inducers phorbol 12-myristate 13-acetate (PMA) and calcium ionophore A23187 (CI) in a concentration- and time-dependent manner (Supplementary Figure S1d−g). Furthermore, when nHL-60 cells were co-cultured with the probiotic strain LGG for 1 h before PMA stimulation, NET level in the supernatant decreased (Supplementary Figure S1h). These results demonstrate that our experimental system is consistent with previous reports and successfully replicates the induction of HL-60 cells into neutrophil-like cells, as well as the inhibition of NET formation when co-cultured with LGG.^25^

**Figure 1.**
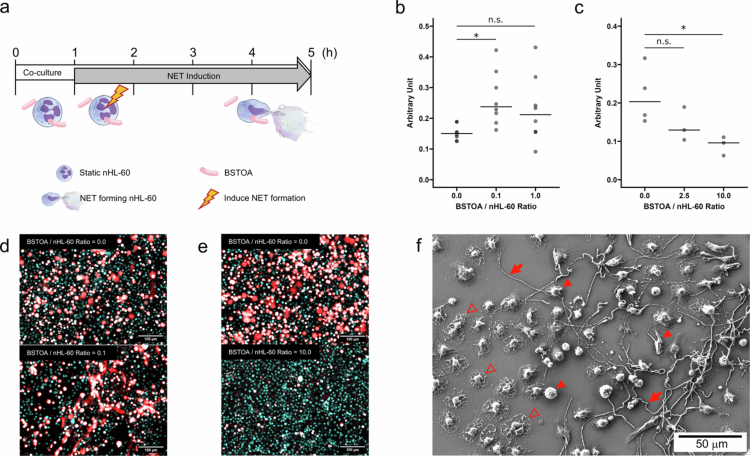
Co-culture with BSTOA alters NET formation in nHL-60 cells. (a) Schematic representation of experimental design. nHL-60 cells were co-cultured with BSTOA at various ratios for 1 h, followed by NET formation induction using PMA for 4 h. Illustrations are sourced from NIAID NIH BIOART (bioart.niaid.nih.gov). (b) BSTOA was added to cultured nHL-60 cells at ratios (BSTOA/nHL-60 cells) of 0.0, 0.1, or 1.0, and (c) 0.0, 2.5, or 10.0, to quantify the NET levels of supernatant using the ELISA method. Data are shown as raw value points with mean bars (*n* > 3 for each condition). *p < 0.05, determined by Dunn's test with Bonferroni correction. (d, e) NET-forming nHL-60 cells were stained and observed via epifluorescence microscopy after 4 h of PMA addition, at the co-culture ratio (BSTOA/nHL-60 cells) of 0.0 (upper) and 0.1 (lower) (d) or 0.0 (upper) and 10.0 (lower) (e). Cyan fluorescence indicates the nuclei of nHL-60 cells, while orange fluorescence indicates NETs. Representative results from three independent experiments were shown. Scale bars: 100 µm. (f) NET- forming nHL-60 cells were fixed and observed via scanning electron microscopy after 4 h of PMA addition, at the co-culture ratio (BSTOA/nHL-60 cells) of 1.0 as described above (b). Solid arrow: BSTOA; solid arrowhead: nHL-60 cells; open arrowhead: NET-forming nHL-60. Scale bars: 50 μm.

The nHL-60 cells were co-cultured with BSTOA vegetative cells at various ratios for 1 h, followed by PMA-induced NET formation (Figure 1a). Co-culture with BSTOA at a ratio of 0.1:1 (BSTOA:nHL-60 cells, BSTOA01) increased the NET levels (Figure 1b and d). On the contrary, co-culture at a ratio of 10:1 (BSTOA:nHL-60 cells, BSTOA10) decreased the NET levels (Figure 1c and e). Although it did not reach statistical significance (*p* = 0.057), in the case of BSTOA10, a similar suppressive effect was observed even when CI was used as a NET inducer instead of PMA, whereas in the case of BSTOA01, it did not affect NET formation (Supplementary Figure S2). In addition, scanning electron microscopy showed that NET formation was suppressed when BSTOA was in close proximity to nHL-60 cells, whereas NET formation occurred in nHL-60 cells separated from BSTOA in the same well (Figure 1f). No detectable NETs were observed in the supernatant of nHL-60 cells co-cultured with BSTOA without the addition of the NET inducer (data not shown). Additionally, based on our limited experiments, the type strain *Bacillus subtilis* NBRC13719^T^ tended to exhibit a trend similar to that of BSTOA; however, co-culture ratios were higher than those of BSTOA, ranging from 1 to 25:1 to increase NET levels, and at 100:1 to decrease NET levels (Supplementary Figure S3).

### Co-culture duration does not affect the modulation of NET formation

**Figure 2.**
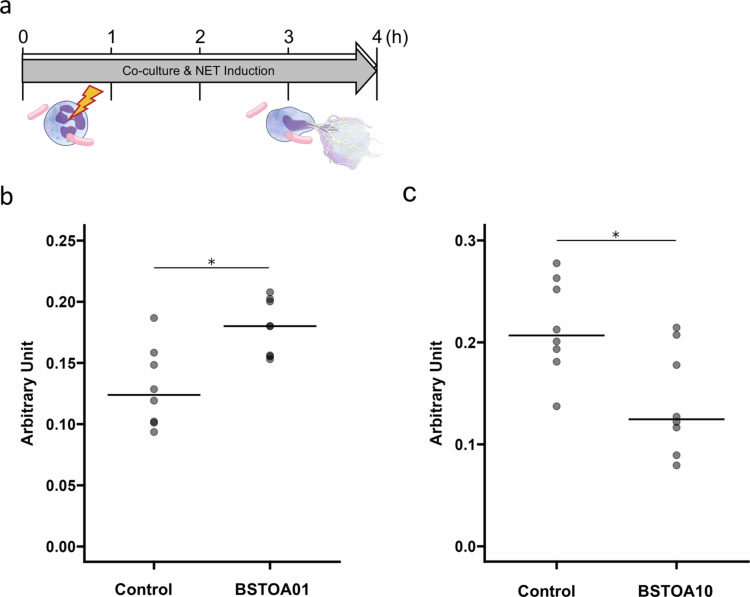
BSTOA modulates NET formation when co-culture starts simultaneously with NET induction. (a) Schematic representation of the experimental design. BSTOA and PMA were added simultaneously to culturing nHL-60 cells from 0 to 4 h of incubation to induce NET formation. Icons are the same as in Figure 1a. (b) BSTOA was added to culturing nHL-60 cells at ratio of 0.0 (Control) or 0.1 (BSTOA01), and (c) 0.0 (Control) or 10.0 (BSTOA10), to quantify the NET levels of supernatant by the ELISA method. Data are shown as raw value points with mean bars (*n* = 8 for each condition). *p < 0.05, determined by the Mann–Whitney *U* test.

To confirm whether a 1-h pre-incubation with BSTOA before the addition of PMA is necessary to modulate NET formation in nHL-60 cells, BSTOA and PMA were simultaneously added to cultured nHL-60 cells, and NET levels were measured (Figure 2a). Even without the pre-co-culture of nHL-60 cells with BSTOA, NET levels increased in the presence of BSTOA01, whereas NET levels decreased in the presence of BSTOA10 (Figure 2b, c). A suppressive effect of NET formation by BSTOA10 still appeared to be observed when BSTOA was added 1 h after PMA induction in our limited experiments (Supplementary Figure S4a, c). In contrast, the promoting effect of NET formation by BSTOA01 was not apparent when BSTOA was added after PMA induction (Supplementary Figure S4b).

### BSTOA10-driven NET formation inhibition is independent of bacterial media component consumption or NET degradation

**Figure 3.**
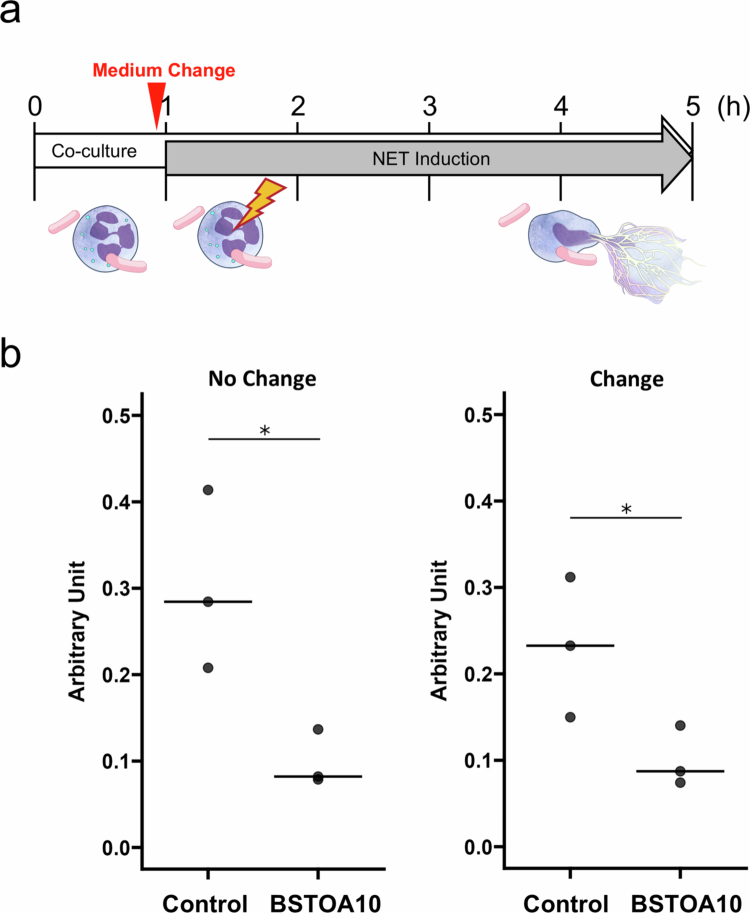
BSTOA10 inhibits NET formation regardless of media change prior to NET induction. (a) Schematic representation of the experimental design. nHL-60 cells were co-cultured with BSTOA for 1 h, followed by NET formation induction using PMA for another 4 h incubation. Icons are the same as in Figure 1a. (b) BSTOA was added to culturing nHL-60 cells at ratios (BSTOA/nHL-60 cells) of 0.0 (Control), or 10.0 (BSTOA10) with (right) or without (left) change of fresh medium before NET induction, to quantify the NET levels of supernatant by the ELISA method. Data are shown as raw value points with mean bars (*n* = 3 for each condition). *p < 0.05, determined by the Mann–Whitney *U* test.

NET formation is known to be inhibited by acidic pH,^36^^,^^37^ glucose depletion,^38^ or hypoxia.^39^ *Bacillus subtilis*, an aerobic bacterium that produces organic acids, may consume glucose and oxygen, resulting in a decrease of pH in the medium. To confirm whether these factors contribute to the suppressive effects of BSTOA10, we replaced the culture medium with fresh medium after co-culturing nHL-60 cells with BSTOA10 and immediately induced NET formation using PMA (Figure 3a). The suppressive effect on NET formation persisted when the culture medium was replaced with fresh medium (Figure 3b, right).

Additionally, many bacteria produce nucleases and/or proteases capable of degrading NET components. To confirm whether BSTOA decreased NET levels through the production of these enzymes, BSTOA10 was added to post-formed NETs in nHL-60 cells and incubated for 4 h (Supplementary Figure S5a). NET levels remained unchanged after BSTOA treatment (Supplementary Figure S5b).

### Exploration of biological processes of nHL-60 cells regarding the modulation of NET formation by BSTOA

**Figure 4.**
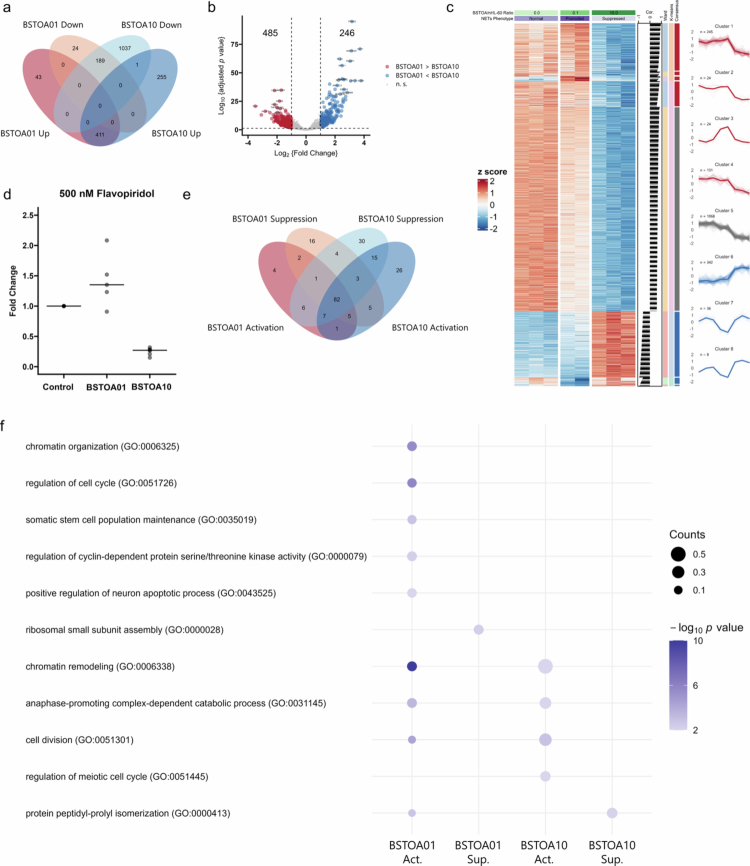
Biological processes of nHL-60 cells regarding the modulation of NET formation by BSTOA. (a) Venn diagram showing the distribution of differentially expressed genes (DEGs) across co-culture conditions compared to those of the control without BSTOA. (b) The volcano plot illustrates the DEGs between BSTOA01 and BSTOA10. Red and blue points indicate DEGs with over two-fold changes in BSTOA01 and BSTOA10, respectively. (c) Heatmap of z-scores with an appended bar plot showing correlations between z-scores and NET modulation phenotypes (Normal = 0, Promoted = 1, and Suppressed = −1), alongside a line plot displaying z-score distributions across eight clusters. (d) Fold-change in NET formation, as shown in Figure 1b and c, with the addition of 500 nM flavopiridol to inhibit *de novo* mRNA expression (*n* = 5). (e) Venn diagram showing the distribution of enriched transcription factors derived from DEGs in each condition. (f) Results of gene ontology enrichment analysis presented as a bubble chart. Bubble color represents significance (– log_10_ *p* value), and bubble size corresponds to the number of assigned genes.

To investigate the changes in gene expression in nHL-60 cells co-cultured with BSTOA, microarray analysis of mRNA was performed on nHL-60 cells co-cultured with BSTOA01 or BSTOA10, and on nHL-60 cells cultured alone (control). Signals were obtained for 7810 probe sets with 4997 functional genes in nHL-60 cells (Supplementary Figure S6). Principal component (PC) analysis was performed using the signals from these 7810 probe sets, revealing that PC1 reflected the presence or absence of BSTOA co-culture, PC2 reflected the difference in the co-culture ratio, and PC3 reflected factors other than the above experimental conditions, with contribution rates of 85.3%, 7.9%, and 2.2%, respectively (Supplementary Figure S6c, d). Compared with the control, the expression of 43 genes were upregulated and 24 genes were downregulated in the case of BSTOA01 (Figure 4a, Supplementary Table S1). In contrast, 255 genes were upregulated and 1027 genes were downregulated in the case of BSTOA10 (Figure 4a, Supplementary Table S1). We also directly compared changes in gene expression between the two co-culture conditions. The expression of 485 genes were upregulated and 246 genes were downregulated in BSTOA01 relative to BSTOA10 (Figure 4b, Supplementary Table S2). Furthermore, the gene expression patterns were classified into eight clusters using hierarchical clustering of z-scores. In the case of BSTOA01, the expression of 24 genes in cluster 3 were upregulated and 36 genes in cluster 7 were downregulated. In the case of BSTOA10, the expression of 342 genes in cluster 6 were upregulated, whereas 245 genes in cluster 1, 24 genes in cluster 2, and 131 genes in cluster 4 were downregulated. Furthermore, eight genes in cluster 8 were downregulated in BSTOA01 and upregulated in BSTOA10 (Figure 4c and Supplementary Table S3).

NET formation occurs independently of *de novo* gene expression and protein translation;^40^ hence, we assessed whether the effects of BSTOA modulation on NET formation required new transcription using the transcription elongation inhibitor, flavopiridol. Both the promoting and suppressive effects of BSTOA were maintained under conditions of inhibited transcriptional elongation (Figure 4d, Supplementary Figure S7b). This result indicated that the modulation of NET formation by BSTOA was independent of *de novo* gene expression, suggesting the need for upstream analysis.

Upstream analyzes were conducted to estimate the changes induced by BSTOA in nHL-60 cells beyond *de novo* gene expression. First, transcription factor enrichment analysis was performed based on the microarray results (Figure 4e, Supplementary Figures S8 and S9, and Supplementary Table S4). For BSTOA01, 4 transcription factors were predicted to be upregulated and 16 transcription factors were predicted to be downregulated (Figure 4e). In contrast, for BSTOA10, 26 transcription factors were predicted to be upregulated, and 30 were predicted to be downregulated (Figure 4e). Next, proteins potentially interacting physically with these transcription factors were identified using a protein-protein interaction database (see Materials and Methods), and gene ontology (GO) enrichment analysis for biological processes was conducted (Figure 4f). GO terms directly related to gene expression or protein translation were excluded (Supplementary Table S5). The analysis suggested that BSTOA01 activated chromatin organization (GO:0006325), cell cycle regulation (GO:0051726), somatic stem cell population maintenance (GO:0035019), cyclin-dependent protein serine/threonine kinase activity regulation (GO:0000079), and neuronal apoptotic processes (GO:0043525). Ribosomal small subunit assembly (GO:0000028) was also predicted to be suppressed. In the case of BSTOA10, the regulation of the meiotic cell cycle (GO:0051445) was expected to be activated. Additionally, chromatin remodeling (GO:0006338), anaphase-promoting complex-dependent catabolic processes (GO:0031145), and cell division (GO:0051301) were activated under both BSTOA01 and BSTOA10 conditions. In contrast, protein peptidyl-prolyl isomerization (GO:0000413) was activated under BSTOA01 conditions, but suppressed under BSTOA10 conditions (Figure 4f).

## Discussion

BSTOA is widely used as an active ingredient in probiotic and symbiotic products in Japan and other countries. These products ameliorate various conditions associated with intestinal dysbiosis, such as infections,^26-29^^,^^41-44^ ulcerative colitis,^30^^,^^31^ and drug-induced gastrointestinal injury.^35^ The pathophysiological associations are bidirectional when considering NETs, suggesting that promoting NET formation enhances pathogen clearance during infections, whereas suppressing it alleviates disease progression in inflammatory disorders.

Herein, we demonstrated for the first time that BSTOA modulates NET formation in neutrophil-like cells in a co-culture ratio-dependent manner *in vitro*. At a low co-culture ratio, specifically BSTOA01, BSTOA promoted NET formation in nHL-60 cells. Under this condition, it is unlikely that every nHL-60 cell can establish direct physical contact with the bacterial cells. In contrast, at a high co-culture ratio, namely BSTOA10, BSTOA suppressed NET formation in nHL-60 cells during both PMA and CI induction (Figure 1, Supplementary Figure S2). Under this condition, most nHL-60 cells should be able to interact physically with bacterial cells. Based on these observations, we hypothesize that unidentified factors secreted by BSTOA promote NET formation, whereas direct contact with the bacterial cells suppresses it.

PMA induces NET formation through the NADPH oxidase (NOX)-dependent pathway, whereas CI operates in a NOX-independent manner.^6^^,^^7^^,^^45^ Several pathogenic organisms, including *Streptococcus aureus*,^39^ *Streptococcus agalactiae*,^6^ *Pseudomonas aeruginosa*,^8-10^*Helicobacter pylori*,^7^ lipopolysaccharide of *Escherichia coli* O26:B6,^10^ human immunodeficiency virus-1,^13^ and opsonized hyphae of *Candida albicans*,^40^ induce NET formation via a NOX-dependent pathway similar to PMA. Therefore, a bidirectional effect of BSTOA on the modulation of NET formation may be observed with these pathogens, as well as with PMA. Conversely, pathogens or compounds such as *Dirofilaria immitis*,^14^ *Candida albicans* hyphae,^6^ and nigericin, which is a potassium ionophore from *Streptomyces hygroscopicus*,^6^ induce NET formation through NOX-independent pathways similar to CI, which is also secreted from the pathogen *Streptomyces chartreusensis*.^6^ BSTOA showed a promoting effect on PMA-induced NET formation at a low ratio (BSTOA01) and a suppressive effect at a high ratio (BSTOA10) (Figure 1). Regarding CI-induced NET formation, it had no effect at the low ratio but showed a similar trend of the suppressive effect (*p* = 0.057) at the high ratio (Supplementary Figure S2). Furthermore, in the NET degradation experiment, the addition of BSTOA did not affect the amounts of NETs (Supplementary Figure S5b). Therefore, BSTOA exerts its effects only during NET formation process; at low ratio, it may promote NET formation via the NOX-dependent pathway, while at high ratio, it may suppress NET formation by acting on both NOX-dependent and independent pathways. Nevertheless, assessing the effects of BSTOA with real pathogenic bacteria remain essential to elucidating the complex, real-world phenomena that reagent-based models cannot fully recapitulate. Furthermore, whether NET formation is likewise regulated in the presence of opportunistic commensal bacteria remains unclear. Establishing advanced multispecies *in vitro* culture systems will be necessary for successful *in vivo* translation.

Although several synthetic drugs influence NET formation, the relationship between these compounds and *B. subtilis* secretions or cellular components remains unclear.^2^ Moreover, some pathogens inhibit NET formation, but they are not taxonomically closely related to *B. subtilis*.^3^ Therefore, our GO analysis revealed novel insights into the regulatory mechanisms of NET formation in neutrophil-like cells co-cultured with *B. subtilis*. Notably, the protein peptidyl-prolyl isomerization (GO:0000413) pathway showed a positive correlation in the case of BSTOA01 and a negative correlation in the case of BSTOA10 on NET formation of nHL-60 cells (Figure 3f). This enzyme positively regulates calcineurin activity in eukaryotic cells. Inhibition of calcineurin activity through cyclophilin or FK506-binding protein, the main peptidyl-prolyl isomerases, suppresses NET formation, irrespective of the NOX dependency of the pathways.^46^ Additionally, genetic overexpression of cyclophilin enhances NET formation, while its deletion reduces it.^47^ These findings suggest that the bidirectional regulation of NET formation promoted and inhibited by BSTOA observed in this study is likely mediated by the regulation of calcineurin via the peptidyl-prolyl isomerase pathway.

Furthermore, the promoting effect of BSTOA01 on NET formation may involve the activation of cell cycle-related pathways such as cyclin-dependent kinase (CDK) regulation (GO:0000079), somatic stem cell population maintenance (GO:0035019), and cell cycle control (GO:0051726) (Figure 3f). Given that neutrophils are terminally differentiated cells, nHL-60 cells were confirmed to be non-proliferative (Supplementary Figure S1a), and these pathways are likely to serve functions beyond cell proliferation. NOX-dependent NET formation requires CDK4/6 activation controlled by p21, and PMA-induced NET formation exhibits cell division-related features within the first hour.^48^ Thus, BSTOA01 may promote NET formation via activation of cell cycle-related signaling pathways. In addition, chromatin remodeling (GO:0006325) was also implicated in the BSTOA01 conditions (Figure 3f). As previously mentioned, neither DNA synthesis nor RNA elongation is associated with NET formation.^40^^,^^48^^,^^49^ Conversely, chromatin decondensation through promoter melting by topoisomerases is essential.^49^ In summary, BSTOA01 could facilitate chromatin remodeling to promote NET formation. However, the details require further investigation.

The suppressive effect of BSTOA10 on NET formation was observed even when BSTOA was added after the addition of PMA (Supplementary Figure S5b). During NOX-independent NET formation, microvesicle shedding, DNA decondensation, nuclear rounding, nuclear membrane rupture, and increased membrane permeability occur in a sequential process for approximately 1 h.^50^^,^^51^ Based on the above reports, BSTOA10 possibly intervenes in cellular responses at or beyond these steps to suppress NET formation.

The data in this study were observed in co-culture system *in vitro* and lacked the tissue compartments present *in vivo*; hence, the relevance of these findings to pathophysiological phenomena *in vivo* must be interpreted cautiously. The normal intestinal barrier consists of a mucus layer, an intestinal epithelial cell layer, and a lamina propria on the luminal side. When infection or non-infectious inflammation occurs in the intestine, neutrophils migrate from the peripheral blood into the lamina propria in response to chemokines produced by intestinal epithelial cells. Ascher *et al*. reported that intestinal bacteria suppress NET formation in a mouse model of mesenteric ischemia-reperfusion injury and that the formation of NETs can be influenced by postnatally developed commensal bacteria, oral antibiotic administration, or re-establishing bacterial communities in mice.^52^ This observation suggests that even in mice with intact intestines with mucus and intestinal epithelial cells, bacteria in the gut lumen can modulate NET formation in the lamina propria in an acquired manner.

Furthermore, neutrophils migrate transepithelially to the luminal side using tight junction proteins and integrins of intestinal epithelial cells as scaffolds, allowing them to interact with bacteria directly.^53^ BSTOA is an aerobic bacterium belonging to the species of *B. subtilis*, primarily active in the oxygen-rich jejunum and ileum when administered orally.^54^ Under healthy conditions, the cooperation between gut microbiota and intestinal epithelial cells helps maintain low oxygen levels on the luminal side, particularly in the colon.^55-57^ However, inflammatory bowel diseases commonly feature dysbiosis with decreased obligate anaerobes and increased facultative anaerobes.^55-60^ This disruption in anaerobiosis may result from reduced host oxygen consumption, increased oxygen diffusion owing to impaired gut barrier, and generated reactive oxygen species, as described in the ‘oxygen hypothesis.’^61^ Accordingly, direct interactions between BSTOA and neutrophils are likely to occur only when neutrophils have migrated into the lumen and local luminal oxygenation is present. Whether BSTOA modulates neutrophil activity under such conditions remains to be determined and warrants future *in vivo* experiments.

This *in vitro* study used nHL-60 cells, which are widely employed as a surrogate for primary cultured neutrophils because they display morphological changes and reactive oxygen-species production comparable to the primary cultured neutrophils.^25^^,^^50^ Nevertheless, the present findings should be complemented and validated with experimental data using primary cultured neutrophils or animal disease models. In addition to the suicidal NETs exclusively focused in this study, future research should investigate the effect of BSTOA on vital NET formation in the presence of platelets,^10^ as well as on NET comprising mitochondrial DNA,^62^^,^^63^ or DNA-containing vesicles.^5^

As mentioned above, BSTOA is used as a cocktail with other probiotic strains such as EFT110 and CBTOA in current clinical use. Investigation of the NET formation modulating effects of these other strains remains an issue and is expected to be addressed in the future.

In conclusion, BSTOA promoted NET formation at low ratios and suppressed it at high ratios in a co-culture system with neutrophil-like cells *in vitro*. To the best of our knowledge, this is the first report on a single bacterial strain that regulates both the promotion and suppression of NET formation, suggesting its potential utility as a preventive or therapeutic agent for NET-related diseases. Although BSTOA-containing probiotics are used for both infectious and non-infectious intestinal diseases, their application could potentially be expanded to therapeutic strategies targeting other NET-associated inflammatory diseases, such as NSAID-induced enteritis.

## Materials and methods

### Bacterial preparation

Probiotic BSTOA was prepared from a factory-manufactured raw material (TOA Biopharma, Tokyo, Japan) by culturing on brain heart infusion (BHI) agar plates (Eiken, Tokyo, Japan) overnight at 37 °C. Probiotic LGG (ATCC53103) and *Bacillus subtilis* NBRC13719^T^ were obtained from respective suppliers and grown on their recommended specific agar plates to harvest colonies.

BSTOA and *B. subtilis* NBRC13719^T^ were pre-cultured in aerobic BHI broth (Eiken) using a shaker at 37 °C for 16 h, followed by another 2 h of main-culture under the same conditions until they reached the mid-log phase. In contrast, LGG was pre-cultured in static Difco^TM^ Lactobacilli MRS broth (Becton Dickinson) at 37 °C for 16 h, followed by another 3 h of main-culture under the same conditions until they reached the mid-log phase. All bacterial cultures were collected by centrifugation (4000 × g, 5 min, room temperature) and washed three times with Gibco^TM^ HBSS containing 25 mM Gibco^TM^ HEPES (both from Thermo Fisher Scientific), hereafter referred to as the assay buffer. Final bacterial densities were measured using a CDA−1000 device (Sysmex, Kobe, Japan) and adjusted to appropriate concentrations for co-culture with mammalian cells.

### Cell culture and co-culture with bacteria

The human promyeloblast cell line HL-60 (ATCC CCL−240^TM^) was cultured in Gibco^TM^ (Thermo Fisher Scientific) supplemented with 20% heat-inactivated HyClone^TM^ FBS (GE Healthcare Life Sciences). Standard non-treated plastic flasks and a humidified incubator (37 °C, 5% CO₂) were used for cell maintenance and differentiation. Cell density was maintained below 1 × 10⁶ cells/mL, and the number of additional in-house passages was limited to 15 times.

To differentiate HL-60 cells into neutrophil-like (nHL-60) cells, the cells were incubated in the maintenance medium supplemented with 1.25% (v/v) DMSO (Fujifilm Wako Pure Chemical, Osaka, Japan) for 5 d. Cell differentiation was confirmed through trypan blue exclusion assays (Thermo Fisher Scientific), May-Grünwald-Giemsa staining (Fujifilm Wako Pure Chemical), and phagocytosis assays using Invitrogen^TM^ pHrodo^TM^ Green *E. coli* BioParticles^TM^ (Thermo Fisher Scientific), following standard protocols. For co-culture experiments, 1 × 10⁵ viable nHL-60 cells were suspended in the assay buffer and plated in Iwaki TC-treated 96-well plates for adherent cell cultures (AGC Techno Glass, Japan). In specific experiments, Flavopiridol (CS-0018, ChemScence, NJ, USA) was added to the assay buffer.

### Quantification and visualization of NETs

NET formation was induced by incubating nHL-60 cells with phorbol 12-myristate 13-acetate (PMA, Fujifilm Wako Pure Chemical) or calcium ionophore A23187 (Abcam) for at least 3 h under CO₂-free conditions. To harvest NET-containing culture supernatants, cells were treated with 0.4 U/mL DNase I (Nippon Gene, Japan), followed by the addition of 2.5 mM EGTA (Fujifilm Wako Pure Chemical) to halt DNase I activity, based on established protocols.^64^^,^^65^ Supernatants were collected via centrifugation (500 × g, 5 min, room temperature) and subjected to ELISA. NET levels were quantified as MPO-DNA complexes using a sandwich ELISA. Anti-myeloperoxidase (MPO) rabbit polyclonal antibody (475915-1MLCN, Merck, 1.17 mg/mL, 1:500 dilution) was used as the capture antibody, while peroxidase-conjugated anti-DNA monoclonal antibody (Clone: MCA-33, bottle 2 of Cell death Detection ELISA kit, 11544675001, Merck, 1:320 dilution) was used as the detection antibody, following previously published methods.^64^^,^^65^ The assay utilized the TMB peroxidase EIA Substrate Kit (Bio-Rad Laboratories) and 1 N H₂SO₄ for colorimetric detection, with absorbance measured at 450 and 650 nm as the reference wavelength. Arbitrary units of NETs were calculated using pseudo-calibration curves generated in each experiment, with R version 4.4.1^66^ and the nplr package version 0.1-7.^67^

For epifluorescence microscopy, NETs were visualized using 100 μg/L Hoechst 34580 (63493, Merck) and 250 nmol/L SYTOX^TM^ Orange (S34861, Thermo Fisher Scientific) staining for 5 min at room temperature. Imaging was performed using a Nikon CFI S Plan Fluor ELWD NAMC 20XC objective lens (Nikon, JP), and digital images were captured with a QImaging RETIGA 2000 R camera (Teledyne Technologies, USA). Image processing was performed using Fiji version 2.9.0.^68^

For scanning electron microscopy, BSTOA and nHL-60 cells were co-cultured on a glass slide coated with a poly-L-lysine solution (P4707, Merck). After NET formation for 4 h, the cells were first fixed with 2.5% (v/v) glutaraldehyde and 2% (w/v) paraformaldehyde, then post-fixed with 1% osmium tetroxide solution (157-01141, Fujifilm Wako Pure Chemical). This was followed by sequential dehydration in graded ethanol, and dried with 1,1,1,3,3,3-hexamethyldisilazane (080-04143, Fujifilm Wako Pure Chemical).^69^ Finally, the samples were coated with a 10 nm layer of platinum–palladium using a magnetron sputter coater MSP-1S (Vacuum Device, Japan) and observed using a tabletop microscopes TM4000PlusII (Hitachi High-Tech, Japan).

### Transcriptome analysis

nHL-60 cells were co-cultured with BSTOA for 1 h, lysed in Invitrogen^TM^ TRIzol^TM^ Reagent (15596026, Thermo Fisher Scientific), and stored at −80 °C until total RNA extraction. RNA extraction and microarray processing were outsourced to Macrogen Japan (Tokyo, JP), following their standard protocols. RNA concentration, purity, and integrity were assessed using a Nanodrop (Thermo Fisher Scientific) and an Agilent 2100 Bioanalyzer (Agilent Technologies, France). RNA samples meeting the following criteria (A260/A280 > 1.5, A260/A230 > 1.0, concentration > 34 ng/μL, total volume > 0.1 μg, rRNA ratio > 1.0, and RIN > 7.0) were used for microarray analysis. The transcriptome was analyzed using the Clariom^TM^ S Assay microarray kit (Thermo Fisher Scientific). Briefly, cDNA synthesis, fragmentation, and biotin-labeling were performed with the GeneChip^TM^ WT (Whole Transcript) Amplification kit and the GeneChip^TM^ WT Terminal Labeling kit, as per the manufacturer's instructions. Labeled DNA (Approximately 5.5 μg) was hybridized to the GeneChip^TM^ Array at 45 °C for 15 h. Hybridized arrays were washed and stained using the GeneChip^TM^ Fluidics Station 450 and scanned on a GCS3000 Scanner. Probe cell intensity data were computed, and CEL files were generated using GeneChip^TM^ Command Console Software.

The data were summarized and normalized using the Signal Space Transformation-Robust Multichip Analysis (SST-RMA) method in the Analysis Power Tools suite, and processed values were exported for further analysis. Batch effects were removed using the sva package (version 3.52.0).^70^ To reduce noise caused by probes with intensity fluctuations near the negative control probes provided by the manufacturer, intensities were classified into two groups using two-component log-normal distribution mixture model implemented in the mixR package (version 0.2.1).^71^ Probes with a 95% probability of being classified into the lower component were considered non-active genes and were filtered out. Principal component analysis (PCA) was performed using the prcomp function in R. Differentially expressed genes (DEGs) were identified using the limma package (version 3.60.6)^72^ with standard settings, and genes with an adjusted *p*-value < 0.05 were considered significant.

For clustering analysis, expression intensities were converted to z-scores. Both hierarchical clustering (*k* = 5) and k-means clustering (*k* = 4) were performed using standard R functions. In hierarchical clustering, Euclidean distance was calculated, and clusters were formed using Ward's method. The optimal number of clusters (*k*) was determined through a comprehensive preliminary analysis using the fviz_nbclust function in the factoextra package (version 1.0.7).^73^

Transcription factor enrichment analysis was conducted on focused gene lists using the TFEA.ChIP (version 1.24.0),^74^ decoupleR (version 2.10.0),^75^ and RegEnrich (version 1.14.0)^76^ packages. Default settings were applied for all methods. When multiple equivalent methods were available, results were merged to ensure robustness. The background gene set and enriched transcription factors were limited to active genes identified earlier. A union transcription factor list combining results from all methods was generated.

To estimate biological processes associated with enriched transcription factors, gene ontology (GO) enrichment analysis was performed using gene symbols of proteins known to physiologically interact with the transcription factors. Protein-protein interaction network data were retrieved from BioGRID,^77^ IntAct,^78^ MINT,^79^ and STRING^80^ databases. GO enrichment analysis was conducted using the topGO package (version 2.56.0),^81^ and GO terms containing keywords related to transcription, including ‘transcription,’ ‘translation,’ ‘expression,’ ‘RNA processing,’ ‘splicing,’ and ‘RNA polymerase,’ were excluded. All settings were default or recommended values.

### Quantitative RT-PCR

nHL-60 cells were collected in tubes, rapidly frozen using liquid nitrogen, and stored at –80 °C until total RNA extraction. Total RNA was extracted using the NucleoSpin® RNA kit (Takara Bio, Shiga, Japan). cDNA synthesis was performed with the PrimeScript^TM^ RT Reagent Kit with gDNA Eraser (Takara Bio). Quantitative PCR was conducted using TB Green® Premix Ex Taq^TM^ II (Takara Bio) on a CFX96 real-time PCR System (Bio-Rad Laboratories). Primer sets for target genes were designed using Primer-BLAST.^82^ The primer sequences used were as follows: CCL20 (forward: 5'-TTTGATGTCAGTGCTGCTA−3', reverse: 5'-TTTTTCTTTGTGTGAAAGATGATA−3') and CCL4L2 (forward: 5'-ACTCTGAGAAAACCTCTTT−3', reverse: 5'-GTCTGAGCCCATTGGTG−3'). 18S rRNA was used as reference gene.^83^

### Statistical analysis

Data are presented as raw value points and mean bars. For comparisons between two groups, we employed the Wilcoxon signed-rank test or Mann–Whitney *U* test using the exactRankTests package (version 0.8−35).^84^ For comparisons among three or more groups, Dunn's test with Bonferroni correction was performed for multiple comparisons using the dunn.test package (version 1.3.6).^85^ The probability value of *p* < 0.05 was considered statistically significant.

## Supplementary Material

Supplementary material **Supplementary Figure S1.** An *in vitro* experimental system was established to investigate the effects of probiotics on NET formation. (a) Cell density during culture with (differentiation + ) or without (differentiation −) 1.25% (v/v) DMSO. Data are shown as raw value points with polynomial approximation curves (*n *> 3 for each day). (b) Parent HL-60 cells (upper panel) and 5-d differentiated nHL-60 cells (lower panel) were stained using May-Grünwald-Giemsa method and observed using standard bright-field microscopy. Black arrowheads indicate lobulated nuclei. Representative results from three independent experiments are shown. Scale bars: 50 µm. (c) 5-d differentiated nHL-60 cells were incubated with Invitrogen^TM^ pHrodo^TM^ Green *E. coli* BioParticles^TM^ for 1 h, and fluorescence was detected using standard flow cytometry (Cell Sorter SH800, Sony). Representative results from three independent experiments are shown. (d-g) Screening conditions for differentiation induction days and concentrations of NET inducers. NET levels were measured as mean fluorescent units (MFU) using a plate reader (Spark®, Tecan). (d, f) HL-60 cells incubated with 1.25% (v/v) DMSO for 4, 5, or 6 d were induced for NET formation using 100 nM PMA (d) or 5 mM CI (f). (e, g) 5-d differentiated nHL-60 cells were stimulated with 0.8–40 nM PMA (e) or 0.8–40 mM CI (g). Representative results from three independent experiments are shown. (h) 5-d differentiated nHL-60 cells were co-cultured with 100-fold LGG for 1 h, followed by NET formation induction with 100 nM PMA. Culture supernatants were collected 4 h after incubation. Data are shown as raw value points with mean bars (*n *= 6 for each condition). *p < 0.05, determined by the Wilcoxon signed-rank test.
**Supplementary Figure S2.** The NET suppressive effect of BSTOA10 was observed when using CI as an inducer. nHL-60 cells were co-cultured with BSTOA at ratios (BSTOA/nHL-60 cells) of 0.0 (Control) or 0.1 (BSTOA01) (a), and 0.0 (Control) or 10 (BSTOA10) (b) for 1 h, followed by NET formation induction using CI for another 4 h of incubation, to quantify the NET levels in the supernatant using ELISA. Data are presented as raw values with mean bars (*n *= 4 per condition). *p*-values determined using the Mann–Whitney *U* test are indicated in each panel.
**Supplementary Figure S3.** Co-culture with *Bacillus subtilis* NBRC13719^T^ for modulation of NET formation in nHL-60 cells. nHL-60 cells were co-cultured with *B. subtilis* NBRC13719^T^ at ratios (NBRC13719/nHL-60 cells) of 0.0–100 for 1 h, followed by NET formation induction using PMA for 4 h, to quantify the NET levels of supernatant using ELISA. Data are shown as raw values with mean bars (*n *= 2 for each condition).
**Supplementary Figure S4.** Suppressive effect of BSTOA10 on NETs was confirmed when BSTOA was added 1 h after PMA-induced NET formation. (a) Schematic representation of the experimental design. BSTOA was added to nHL-60 cells after 1 h PMA addition and incubated for 4 h. Icons are the same as in Figure 1a. (b) BSTOA was added to nHL-60 cells at ratios (BSTOA/nHL-60 cells) of 0.0 (Control) or 0.1 (BSTOA01), and (c) 0.0 (Control) or 10.0 (BSTOA10), to quantify the NET levels of supernatant using ELISA. Data are shown as raw value points with mean bars (*n *= 2 for each condition).
**Supplementary Figure S5.** BSTOA does not degrade NETs even after 4 h of incubation. (a) Schematic representation of the experimental design. BSTOA was added to NET-formed nHL-60 cells and incubated for 4 h. (b) BSTOA was added to NET-formed nHL-60 cells at the ratios (BSTOA/nHL-60 cells) of 0.0 (Control) or 10.0 (BSTOA10), to quantify the NET levels of supernatant using ELISA. Data are shown as raw value points with mean bars (*n *= 4 for each condition). *p*-value determined by the Mann-Whitney *U* test is described in the panel.
**Supplementary Figure S6.** Two-component log-normal distribution mixture model and principal component (PC) analysis were used to analyze probe intensities and active genes. (a) Distributions of preprocessed intensities. Line colors indicate each sample. (b) Example result of fitting to a two-component log-normal distribution mixture model. Colored solid lines indicate the intensity distributions of probes assigned to each component. The dashed line indicates the overall intensity distribution of all probes. Probes assigned to components 1 and 2 are distributed in the gray and white parts of the histogram, respectively. (c, d) Principal component analysis scatter plots. Point shapes and colors indicate biological replicates and co-culture conditions, respectively. (c) Relations between PC1 and PC2. (d) Relations between PC2 and PC3.
**Supplementary Figure S7.** Flavopiridol inhibits *de novo* gene transcription without interfering with NET formation. (a) nHL-60 cells were co-cultured with BSTOA at ratios (BSTOA/nHL-60 cells) of 0.0, 0.1, or 10 for 1 h with 100−500 nM flavopiridol, followed by quantification of *CCL20* (left) and *CCL4L2* (right) expression normalized to 18S rRNA. Data are shown as raw value points with mean bars (*n *> 3 for each condition). (b) nHL-60 cells were stimulated with DMSO (left), 10 (middle), or 100 nM PMA (right) in assay buffer supplemented with 300−500 nM flavopiridol. Data are shown as raw value points with mean bars (*n *= 4 for each condition).
**Supplementary Figure S8.** Number of transcription factors identified through enrichment analysis was determined for each gene list. nHL-60 cells were co-cultured with BSTOA for 1 h, followed by induction of NET formation using PMA for 4 h. BSTOA was added to the nHL-60 cells at ratios (BSTOA/nHL-60 cells) of 0.0 (Control), 0.1 (BSTOA01), or 10.0 (BSTOA10), for transcription factors analysis. Upregulated and downregulated genes in the BSTOA01 and BSTOA10 conditions were determined using differential expression analysis and z-score clustering. Venn diagrams indicate the distribution of transcription factors identified by transcription factor enrichment analysis.
**Supplementary Figure S9.** Transcription factor distributions were analyzed from gene lists using Venn diagrams. nHL-60 cells were co-cultured with BSTOA for 1 h, followed by induction of NET formation using PMA for 4 h. BSTOA was added to the nHL-60 cells at ratios (BSTOA/nHL-60 cells) of 0.0 (Control), 0.1 (BSTOA01) or 10.0 (BSTOA10), for transcription factors analysis. Venn diagrams showing the intersections of genes identified using different methods, focusing on upregulated (a, c) and downregulated (b, d) genes in BSTOA01 (a, b) and BSTOA10 (c, d).

Supplementary material **Supplementary Table S1.** Differentially expressed genes in BSTOA01 and BSTOA10 compared with Control.
**Supplementary Table S2.** Differentially expressed genes in BSTOA01 compared with BSTOA10.
**Supplementary Table S3.** Genes in clusters showing z-score changes between BSTOA01 and BSTOA10.
**Supplementary Table S4.** Enriched transcription factor genes in each cluster.
**Supplementary Table S5.** List of excluded GO terms.

## Data Availability

Supplementary tables and the source data that support the findings of this study are available from the corresponding author, T.H., upon reasonable request. They can be downloaded from the repository site (https://doi.org/10.5281/zenodo.15589039) once access has been granted.
