# Supplementary figures and images for: Probiotic strain Bacillus subtilis TO-A modulates the formation of neutrophil extracellular traps
Source: Gut Microbes Rep. 2025 Oct 29;2(1):2572788. doi: 10.1080/29933935.2025.2572788 (PMC12940209; doi:10.1080/29933935.2025.2572788)

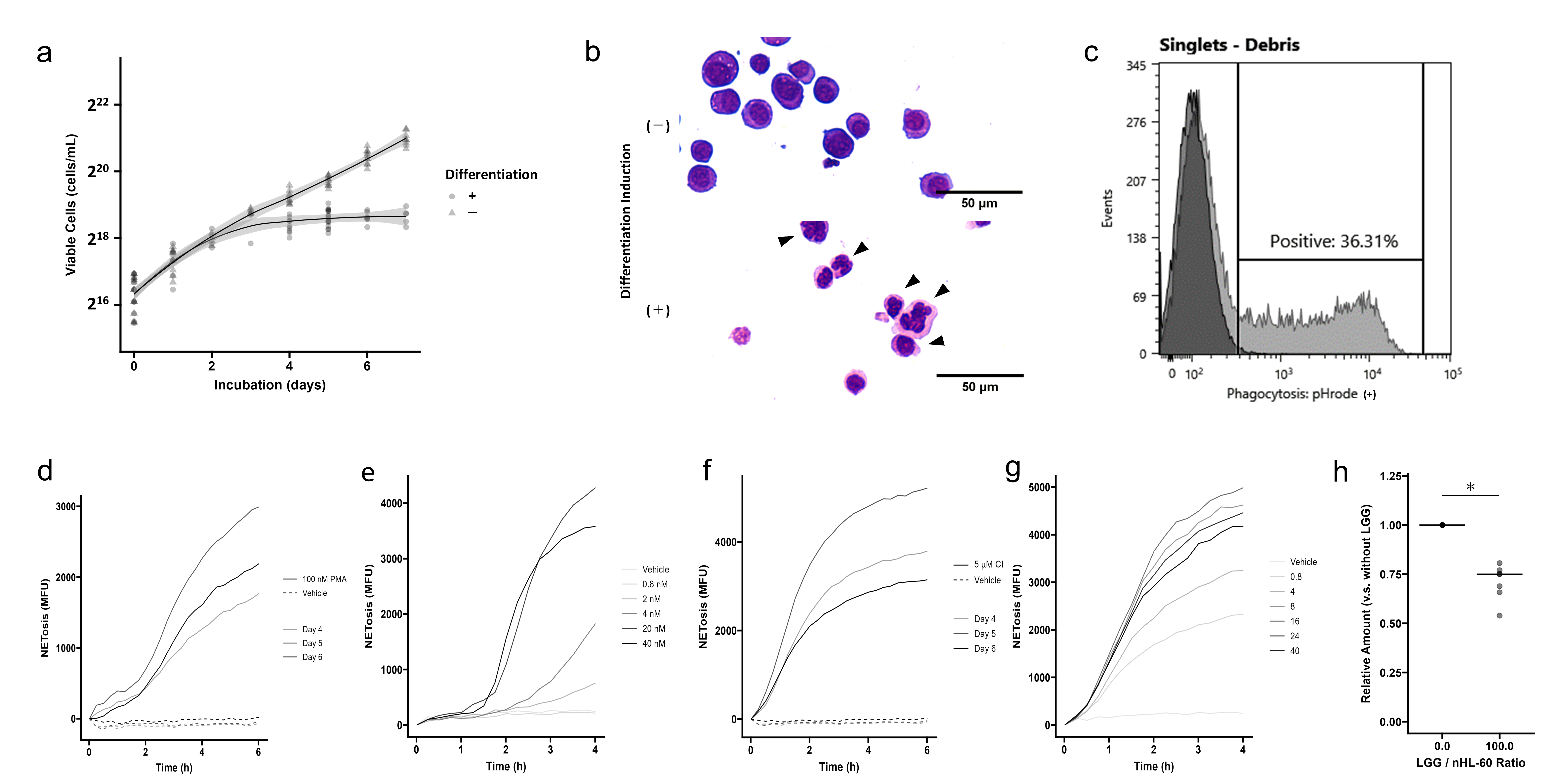

Supplement: Supplementary material — Supplementary Figure S1. An in vitro experimental system was established to investigate the effects of probiotics on NET formation. (a) Cell density during culture with (differentiation + ) or without (differentiation −) 1.25% (v/v) DMSO. Data are shown as raw value points with polynomial approximation curves (n > 3 for each day). (b) Parent HL-60 cells (upper panel) and 5-d differentiated nHL-60 cells (lower panel) were stained using May-Grünwald-Giemsa method and observed using standard bright-field microscopy. Black arrowheads indicate lobulated nuclei. Representative results from three independent experiments are shown. Scale bars: 50 µm. (c) 5-d differentiated nHL-60 cells were incubated with InvitrogenTM pHrodoTM Green E. coli BioParticlesTM for 1 h, and fluorescence was detected using standard flow cytometry (Cell Sorter SH800, Sony). Representative results from three independent experiments are shown. (d-g) Screening conditions for differentiation induction days and concentrations of NET inducers. NET levels were measured as mean fluorescent units (MFU) using a plate reader (Spark®, Tecan). (d, f) HL-60 cells incubated with 1.25% (v/v) DMSO for 4, 5, or 6 d were induced for NET formation using 100 nM PMA (d) or 5 mM CI (f). (e, g) 5-d differentiated nHL-60 cells were stimulated with 0.8–40 nM PMA (e) or 0.8–40 mM CI (g). Representative results from three independent experiments are shown. (h) 5-d differentiated nHL-60 cells were co-cultured with 100-fold LGG for 1 h, followed by NET formation induction with 100 nM PMA. Culture supernatants were collected 4 h after incubation. Data are shown as raw value points with mean bars (n = 6 for each condition). *p < 0.05, determined by the Wilcoxon signed-rank test. Supplementary Figure S2. The NET suppressive effect of BSTOA10 was observed when using CI as an inducer. nHL-60 cells were co-cultured with BSTOA at ratios (BSTOA/nHL-60 cells) of 0.0 (Control) or 0.1 (BSTOA01) (a), and 0.0 (Control) or 10 (BSTOA10) (b) f [file KGMR_A_2572788_SM7230.zip › Supplementary_Figures/FigureS1.tif]

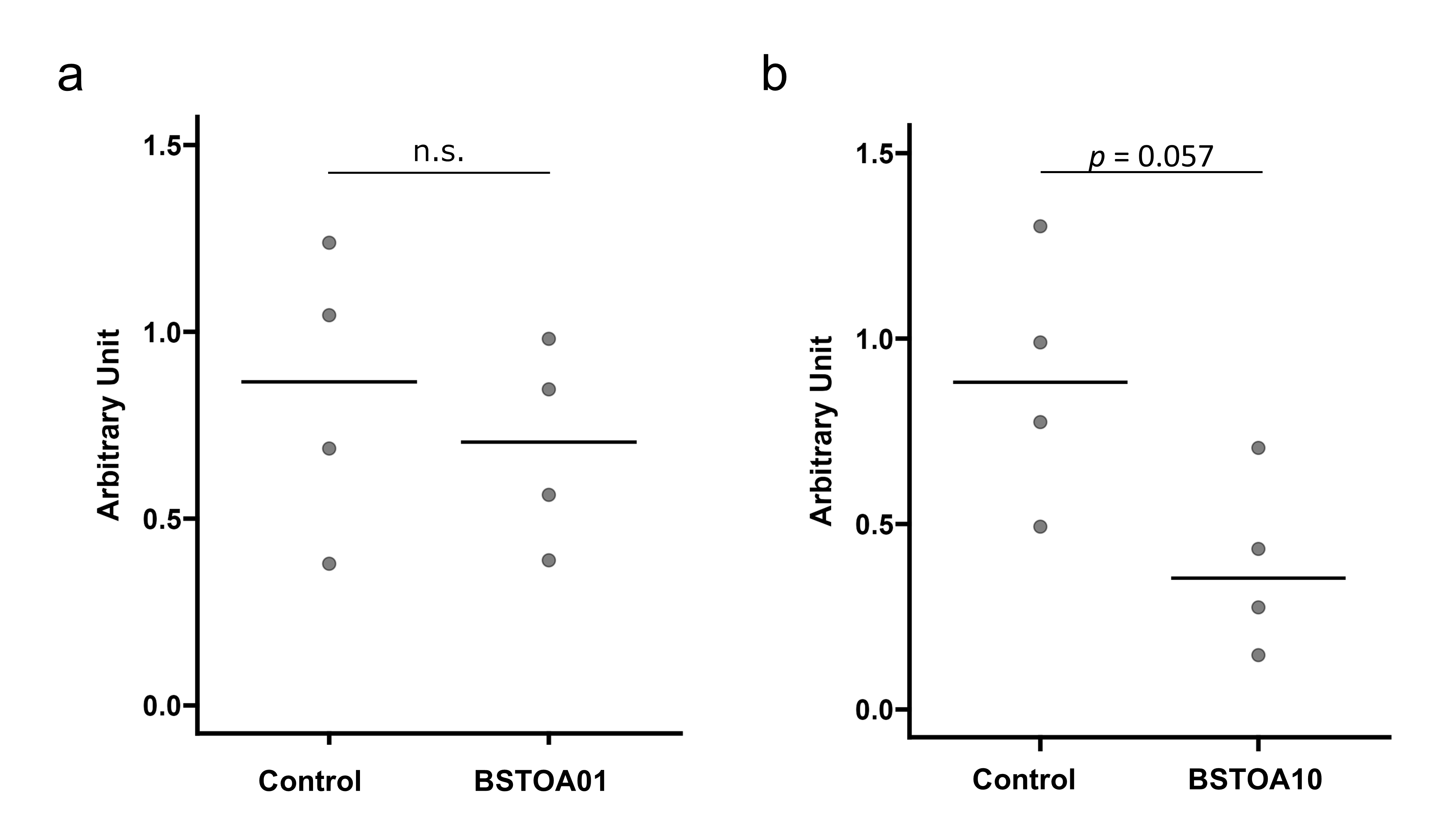

Supplement: Supplementary material — Supplementary Figure S1. An in vitro experimental system was established to investigate the effects of probiotics on NET formation. (a) Cell density during culture with (differentiation + ) or without (differentiation −) 1.25% (v/v) DMSO. Data are shown as raw value points with polynomial approximation curves (n > 3 for each day). (b) Parent HL-60 cells (upper panel) and 5-d differentiated nHL-60 cells (lower panel) were stained using May-Grünwald-Giemsa method and observed using standard bright-field microscopy. Black arrowheads indicate lobulated nuclei. Representative results from three independent experiments are shown. Scale bars: 50 µm. (c) 5-d differentiated nHL-60 cells were incubated with InvitrogenTM pHrodoTM Green E. coli BioParticlesTM for 1 h, and fluorescence was detected using standard flow cytometry (Cell Sorter SH800, Sony). Representative results from three independent experiments are shown. (d-g) Screening conditions for differentiation induction days and concentrations of NET inducers. NET levels were measured as mean fluorescent units (MFU) using a plate reader (Spark®, Tecan). (d, f) HL-60 cells incubated with 1.25% (v/v) DMSO for 4, 5, or 6 d were induced for NET formation using 100 nM PMA (d) or 5 mM CI (f). (e, g) 5-d differentiated nHL-60 cells were stimulated with 0.8–40 nM PMA (e) or 0.8–40 mM CI (g). Representative results from three independent experiments are shown. (h) 5-d differentiated nHL-60 cells were co-cultured with 100-fold LGG for 1 h, followed by NET formation induction with 100 nM PMA. Culture supernatants were collected 4 h after incubation. Data are shown as raw value points with mean bars (n = 6 for each condition). *p < 0.05, determined by the Wilcoxon signed-rank test. Supplementary Figure S2. The NET suppressive effect of BSTOA10 was observed when using CI as an inducer. nHL-60 cells were co-cultured with BSTOA at ratios (BSTOA/nHL-60 cells) of 0.0 (Control) or 0.1 (BSTOA01) (a), and 0.0 (Control) or 10 (BSTOA10) (b) f [file KGMR_A_2572788_SM7230.zip › Supplementary_Figures/FigureS2.tif]

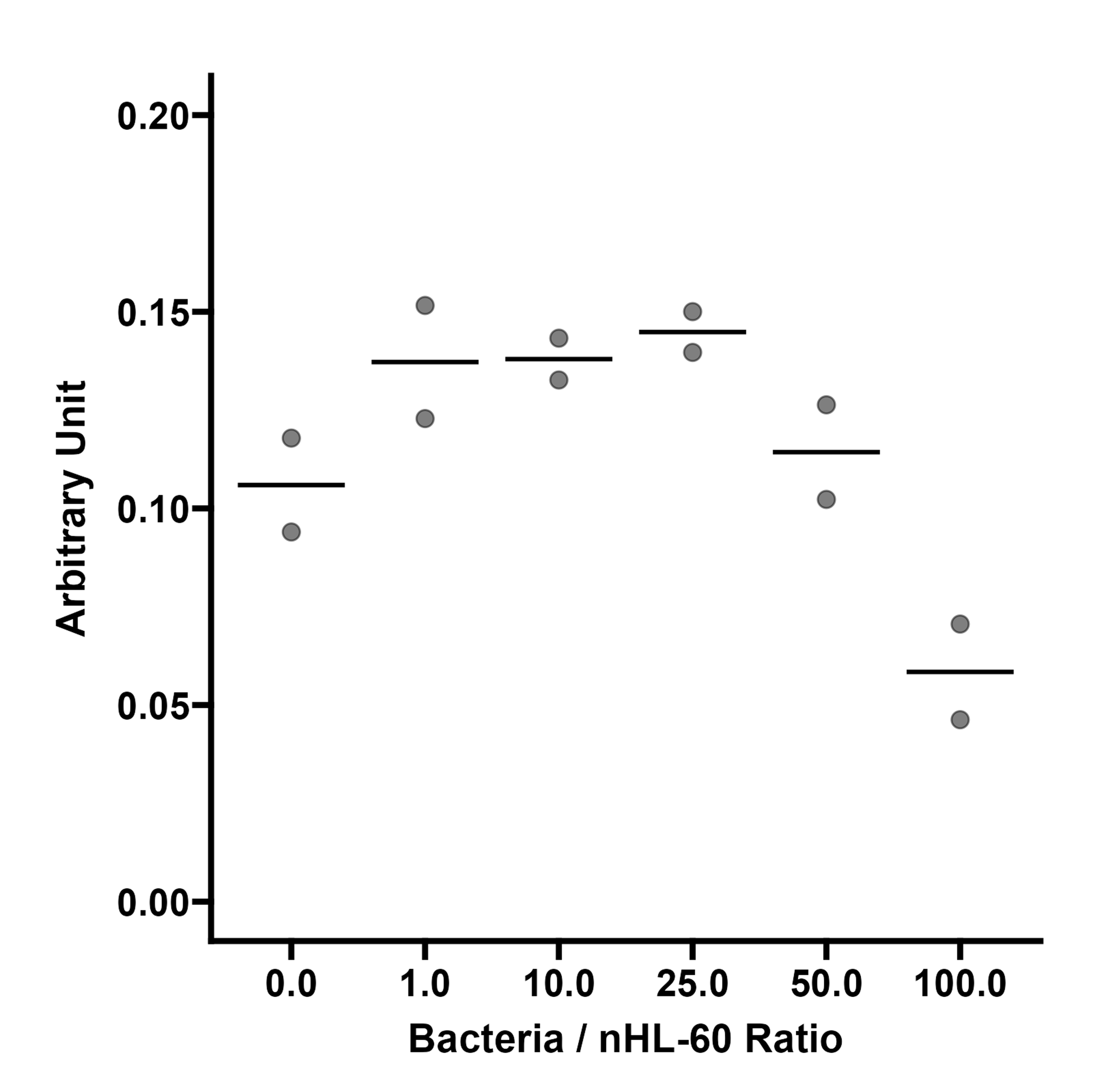

Supplement: Supplementary material — Supplementary Figure S1. An in vitro experimental system was established to investigate the effects of probiotics on NET formation. (a) Cell density during culture with (differentiation + ) or without (differentiation −) 1.25% (v/v) DMSO. Data are shown as raw value points with polynomial approximation curves (n > 3 for each day). (b) Parent HL-60 cells (upper panel) and 5-d differentiated nHL-60 cells (lower panel) were stained using May-Grünwald-Giemsa method and observed using standard bright-field microscopy. Black arrowheads indicate lobulated nuclei. Representative results from three independent experiments are shown. Scale bars: 50 µm. (c) 5-d differentiated nHL-60 cells were incubated with InvitrogenTM pHrodoTM Green E. coli BioParticlesTM for 1 h, and fluorescence was detected using standard flow cytometry (Cell Sorter SH800, Sony). Representative results from three independent experiments are shown. (d-g) Screening conditions for differentiation induction days and concentrations of NET inducers. NET levels were measured as mean fluorescent units (MFU) using a plate reader (Spark®, Tecan). (d, f) HL-60 cells incubated with 1.25% (v/v) DMSO for 4, 5, or 6 d were induced for NET formation using 100 nM PMA (d) or 5 mM CI (f). (e, g) 5-d differentiated nHL-60 cells were stimulated with 0.8–40 nM PMA (e) or 0.8–40 mM CI (g). Representative results from three independent experiments are shown. (h) 5-d differentiated nHL-60 cells were co-cultured with 100-fold LGG for 1 h, followed by NET formation induction with 100 nM PMA. Culture supernatants were collected 4 h after incubation. Data are shown as raw value points with mean bars (n = 6 for each condition). *p < 0.05, determined by the Wilcoxon signed-rank test. Supplementary Figure S2. The NET suppressive effect of BSTOA10 was observed when using CI as an inducer. nHL-60 cells were co-cultured with BSTOA at ratios (BSTOA/nHL-60 cells) of 0.0 (Control) or 0.1 (BSTOA01) (a), and 0.0 (Control) or 10 (BSTOA10) (b) f [file KGMR_A_2572788_SM7230.zip › Supplementary_Figures/FigureS3.tif]

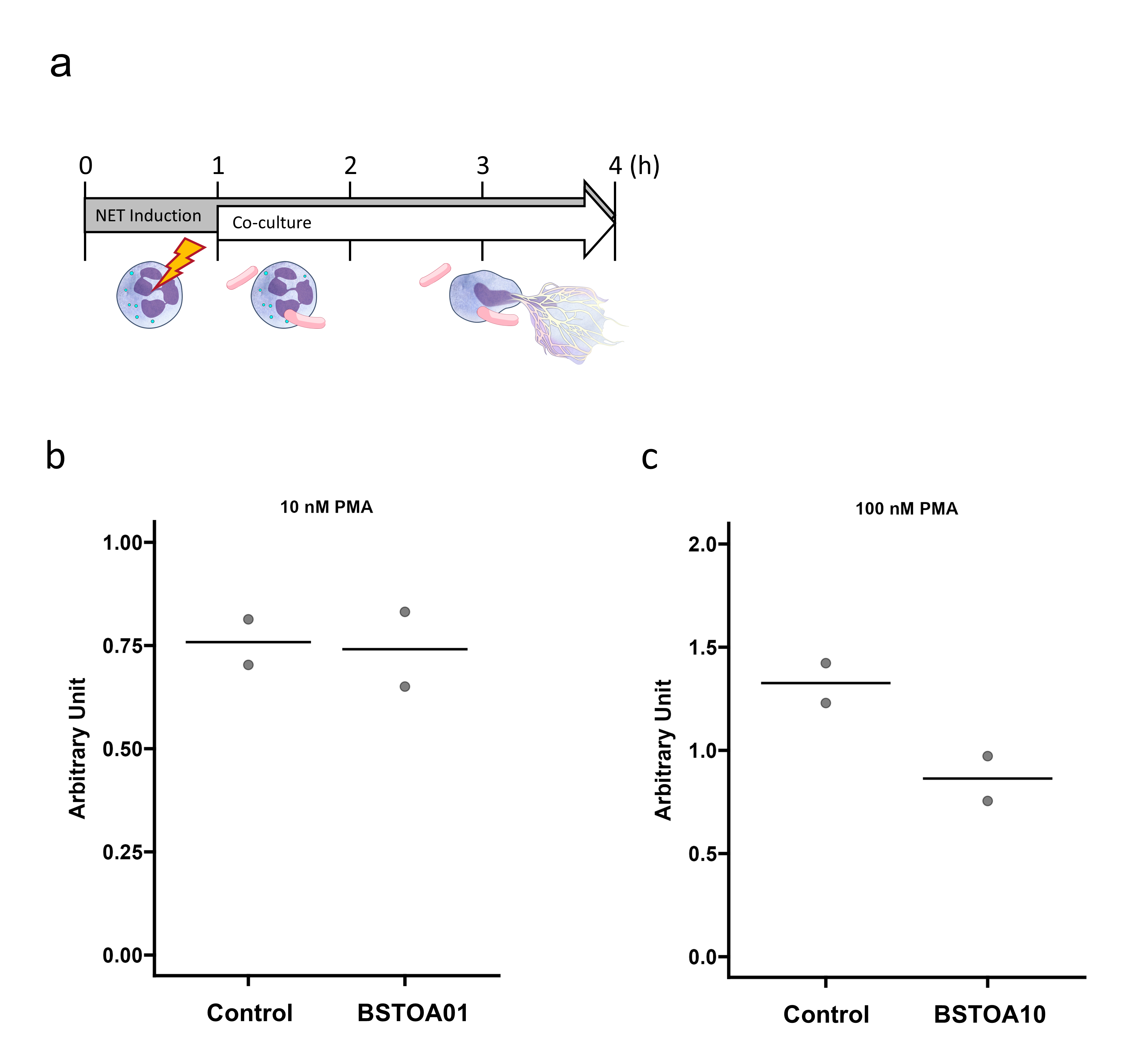

Supplement: Supplementary material — Supplementary Figure S1. An in vitro experimental system was established to investigate the effects of probiotics on NET formation. (a) Cell density during culture with (differentiation + ) or without (differentiation −) 1.25% (v/v) DMSO. Data are shown as raw value points with polynomial approximation curves (n > 3 for each day). (b) Parent HL-60 cells (upper panel) and 5-d differentiated nHL-60 cells (lower panel) were stained using May-Grünwald-Giemsa method and observed using standard bright-field microscopy. Black arrowheads indicate lobulated nuclei. Representative results from three independent experiments are shown. Scale bars: 50 µm. (c) 5-d differentiated nHL-60 cells were incubated with InvitrogenTM pHrodoTM Green E. coli BioParticlesTM for 1 h, and fluorescence was detected using standard flow cytometry (Cell Sorter SH800, Sony). Representative results from three independent experiments are shown. (d-g) Screening conditions for differentiation induction days and concentrations of NET inducers. NET levels were measured as mean fluorescent units (MFU) using a plate reader (Spark®, Tecan). (d, f) HL-60 cells incubated with 1.25% (v/v) DMSO for 4, 5, or 6 d were induced for NET formation using 100 nM PMA (d) or 5 mM CI (f). (e, g) 5-d differentiated nHL-60 cells were stimulated with 0.8–40 nM PMA (e) or 0.8–40 mM CI (g). Representative results from three independent experiments are shown. (h) 5-d differentiated nHL-60 cells were co-cultured with 100-fold LGG for 1 h, followed by NET formation induction with 100 nM PMA. Culture supernatants were collected 4 h after incubation. Data are shown as raw value points with mean bars (n = 6 for each condition). *p < 0.05, determined by the Wilcoxon signed-rank test. Supplementary Figure S2. The NET suppressive effect of BSTOA10 was observed when using CI as an inducer. nHL-60 cells were co-cultured with BSTOA at ratios (BSTOA/nHL-60 cells) of 0.0 (Control) or 0.1 (BSTOA01) (a), and 0.0 (Control) or 10 (BSTOA10) (b) f [file KGMR_A_2572788_SM7230.zip › Supplementary_Figures/FigureS4.tif]

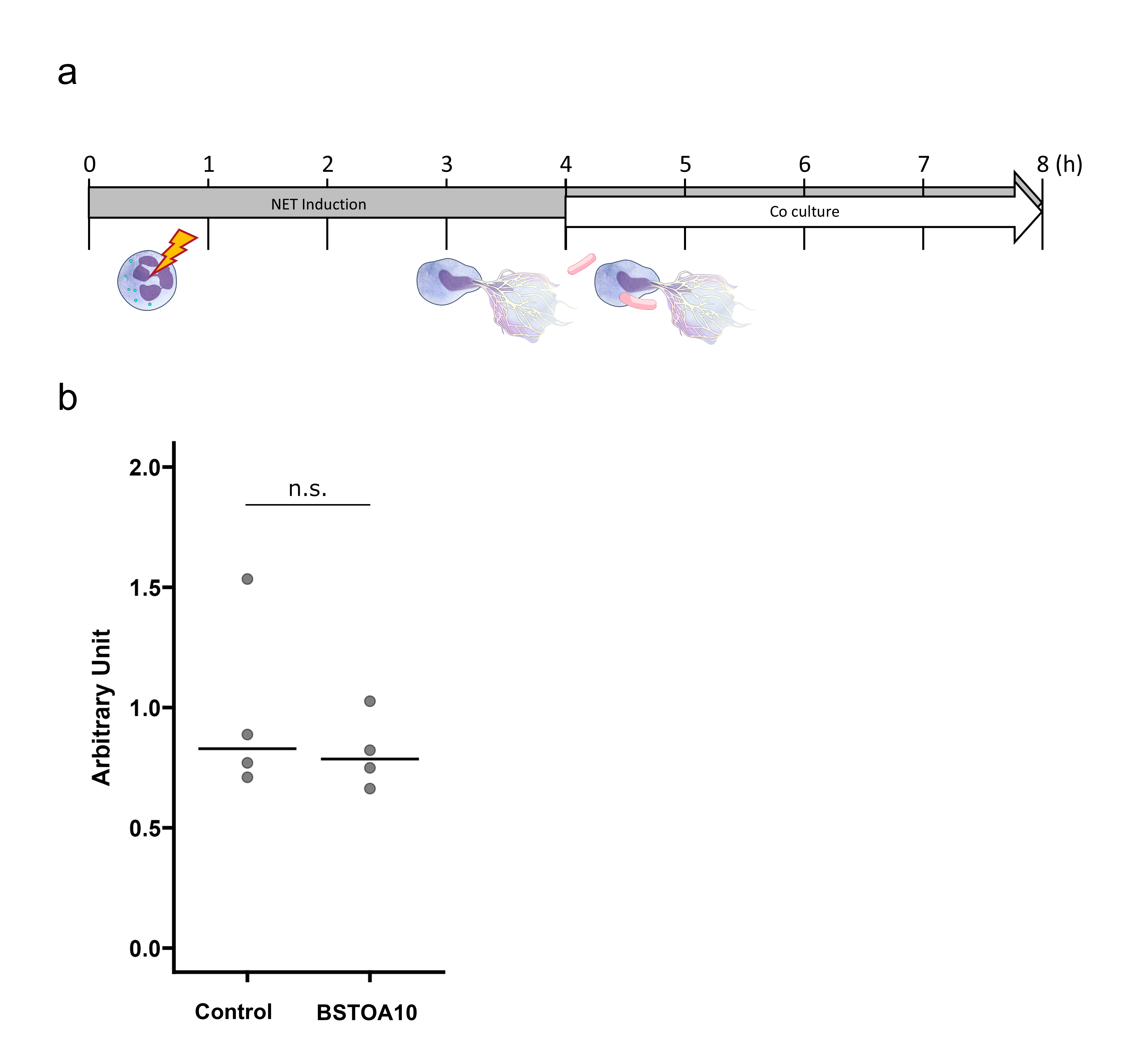

Supplement: Supplementary material — Supplementary Figure S1. An in vitro experimental system was established to investigate the effects of probiotics on NET formation. (a) Cell density during culture with (differentiation + ) or without (differentiation −) 1.25% (v/v) DMSO. Data are shown as raw value points with polynomial approximation curves (n > 3 for each day). (b) Parent HL-60 cells (upper panel) and 5-d differentiated nHL-60 cells (lower panel) were stained using May-Grünwald-Giemsa method and observed using standard bright-field microscopy. Black arrowheads indicate lobulated nuclei. Representative results from three independent experiments are shown. Scale bars: 50 µm. (c) 5-d differentiated nHL-60 cells were incubated with InvitrogenTM pHrodoTM Green E. coli BioParticlesTM for 1 h, and fluorescence was detected using standard flow cytometry (Cell Sorter SH800, Sony). Representative results from three independent experiments are shown. (d-g) Screening conditions for differentiation induction days and concentrations of NET inducers. NET levels were measured as mean fluorescent units (MFU) using a plate reader (Spark®, Tecan). (d, f) HL-60 cells incubated with 1.25% (v/v) DMSO for 4, 5, or 6 d were induced for NET formation using 100 nM PMA (d) or 5 mM CI (f). (e, g) 5-d differentiated nHL-60 cells were stimulated with 0.8–40 nM PMA (e) or 0.8–40 mM CI (g). Representative results from three independent experiments are shown. (h) 5-d differentiated nHL-60 cells were co-cultured with 100-fold LGG for 1 h, followed by NET formation induction with 100 nM PMA. Culture supernatants were collected 4 h after incubation. Data are shown as raw value points with mean bars (n = 6 for each condition). *p < 0.05, determined by the Wilcoxon signed-rank test. Supplementary Figure S2. The NET suppressive effect of BSTOA10 was observed when using CI as an inducer. nHL-60 cells were co-cultured with BSTOA at ratios (BSTOA/nHL-60 cells) of 0.0 (Control) or 0.1 (BSTOA01) (a), and 0.0 (Control) or 10 (BSTOA10) (b) f [file KGMR_A_2572788_SM7230.zip › Supplementary_Figures/FigureS5.tif]

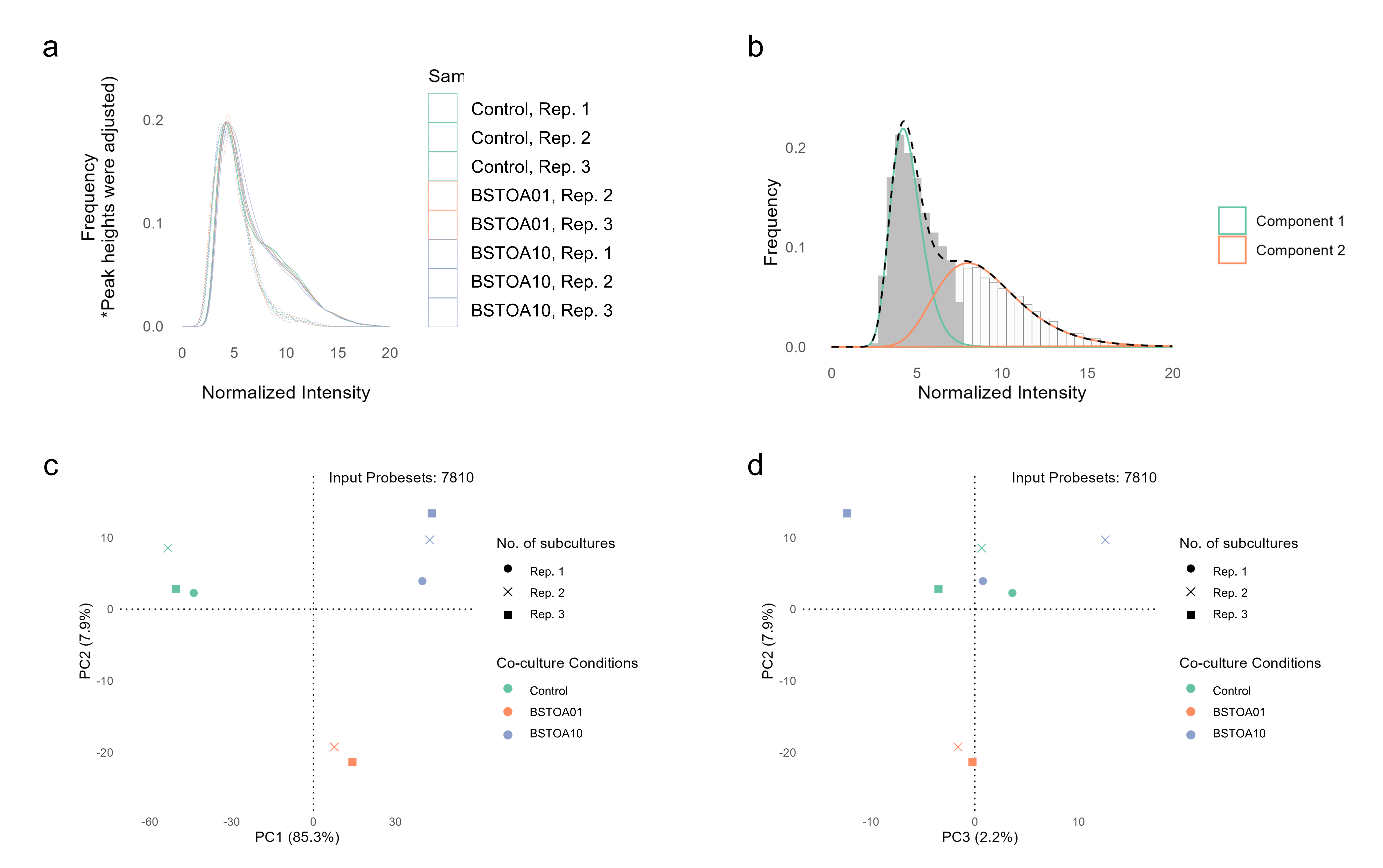

Supplement: Supplementary material — Supplementary Figure S1. An in vitro experimental system was established to investigate the effects of probiotics on NET formation. (a) Cell density during culture with (differentiation + ) or without (differentiation −) 1.25% (v/v) DMSO. Data are shown as raw value points with polynomial approximation curves (n > 3 for each day). (b) Parent HL-60 cells (upper panel) and 5-d differentiated nHL-60 cells (lower panel) were stained using May-Grünwald-Giemsa method and observed using standard bright-field microscopy. Black arrowheads indicate lobulated nuclei. Representative results from three independent experiments are shown. Scale bars: 50 µm. (c) 5-d differentiated nHL-60 cells were incubated with InvitrogenTM pHrodoTM Green E. coli BioParticlesTM for 1 h, and fluorescence was detected using standard flow cytometry (Cell Sorter SH800, Sony). Representative results from three independent experiments are shown. (d-g) Screening conditions for differentiation induction days and concentrations of NET inducers. NET levels were measured as mean fluorescent units (MFU) using a plate reader (Spark®, Tecan). (d, f) HL-60 cells incubated with 1.25% (v/v) DMSO for 4, 5, or 6 d were induced for NET formation using 100 nM PMA (d) or 5 mM CI (f). (e, g) 5-d differentiated nHL-60 cells were stimulated with 0.8–40 nM PMA (e) or 0.8–40 mM CI (g). Representative results from three independent experiments are shown. (h) 5-d differentiated nHL-60 cells were co-cultured with 100-fold LGG for 1 h, followed by NET formation induction with 100 nM PMA. Culture supernatants were collected 4 h after incubation. Data are shown as raw value points with mean bars (n = 6 for each condition). *p < 0.05, determined by the Wilcoxon signed-rank test. Supplementary Figure S2. The NET suppressive effect of BSTOA10 was observed when using CI as an inducer. nHL-60 cells were co-cultured with BSTOA at ratios (BSTOA/nHL-60 cells) of 0.0 (Control) or 0.1 (BSTOA01) (a), and 0.0 (Control) or 10 (BSTOA10) (b) f [file KGMR_A_2572788_SM7230.zip › Supplementary_Figures/FigureS6.tif]

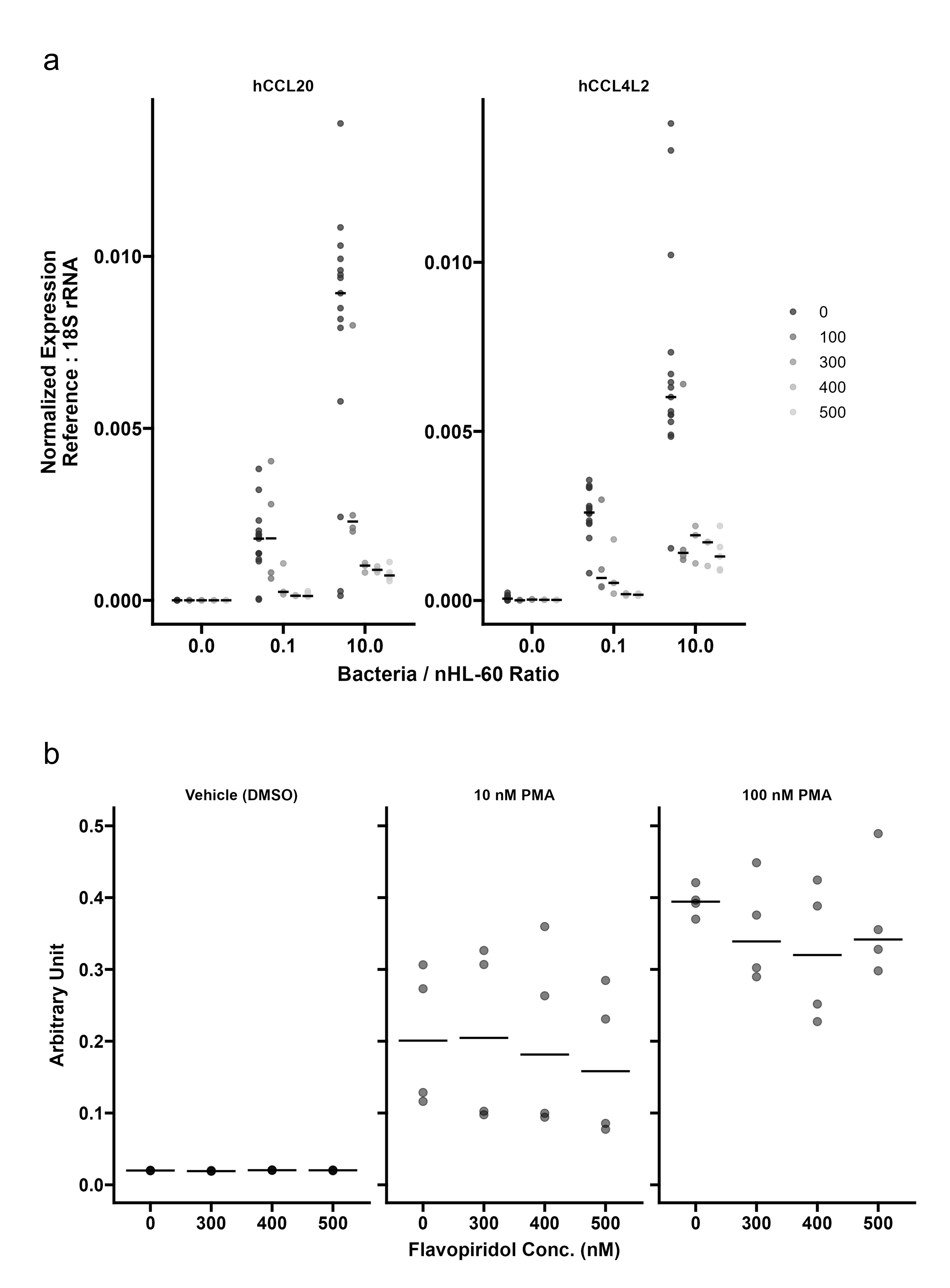

Supplement: Supplementary material — Supplementary Figure S1. An in vitro experimental system was established to investigate the effects of probiotics on NET formation. (a) Cell density during culture with (differentiation + ) or without (differentiation −) 1.25% (v/v) DMSO. Data are shown as raw value points with polynomial approximation curves (n > 3 for each day). (b) Parent HL-60 cells (upper panel) and 5-d differentiated nHL-60 cells (lower panel) were stained using May-Grünwald-Giemsa method and observed using standard bright-field microscopy. Black arrowheads indicate lobulated nuclei. Representative results from three independent experiments are shown. Scale bars: 50 µm. (c) 5-d differentiated nHL-60 cells were incubated with InvitrogenTM pHrodoTM Green E. coli BioParticlesTM for 1 h, and fluorescence was detected using standard flow cytometry (Cell Sorter SH800, Sony). Representative results from three independent experiments are shown. (d-g) Screening conditions for differentiation induction days and concentrations of NET inducers. NET levels were measured as mean fluorescent units (MFU) using a plate reader (Spark®, Tecan). (d, f) HL-60 cells incubated with 1.25% (v/v) DMSO for 4, 5, or 6 d were induced for NET formation using 100 nM PMA (d) or 5 mM CI (f). (e, g) 5-d differentiated nHL-60 cells were stimulated with 0.8–40 nM PMA (e) or 0.8–40 mM CI (g). Representative results from three independent experiments are shown. (h) 5-d differentiated nHL-60 cells were co-cultured with 100-fold LGG for 1 h, followed by NET formation induction with 100 nM PMA. Culture supernatants were collected 4 h after incubation. Data are shown as raw value points with mean bars (n = 6 for each condition). *p < 0.05, determined by the Wilcoxon signed-rank test. Supplementary Figure S2. The NET suppressive effect of BSTOA10 was observed when using CI as an inducer. nHL-60 cells were co-cultured with BSTOA at ratios (BSTOA/nHL-60 cells) of 0.0 (Control) or 0.1 (BSTOA01) (a), and 0.0 (Control) or 10 (BSTOA10) (b) f [file KGMR_A_2572788_SM7230.zip › Supplementary_Figures/FigureS7.tif]

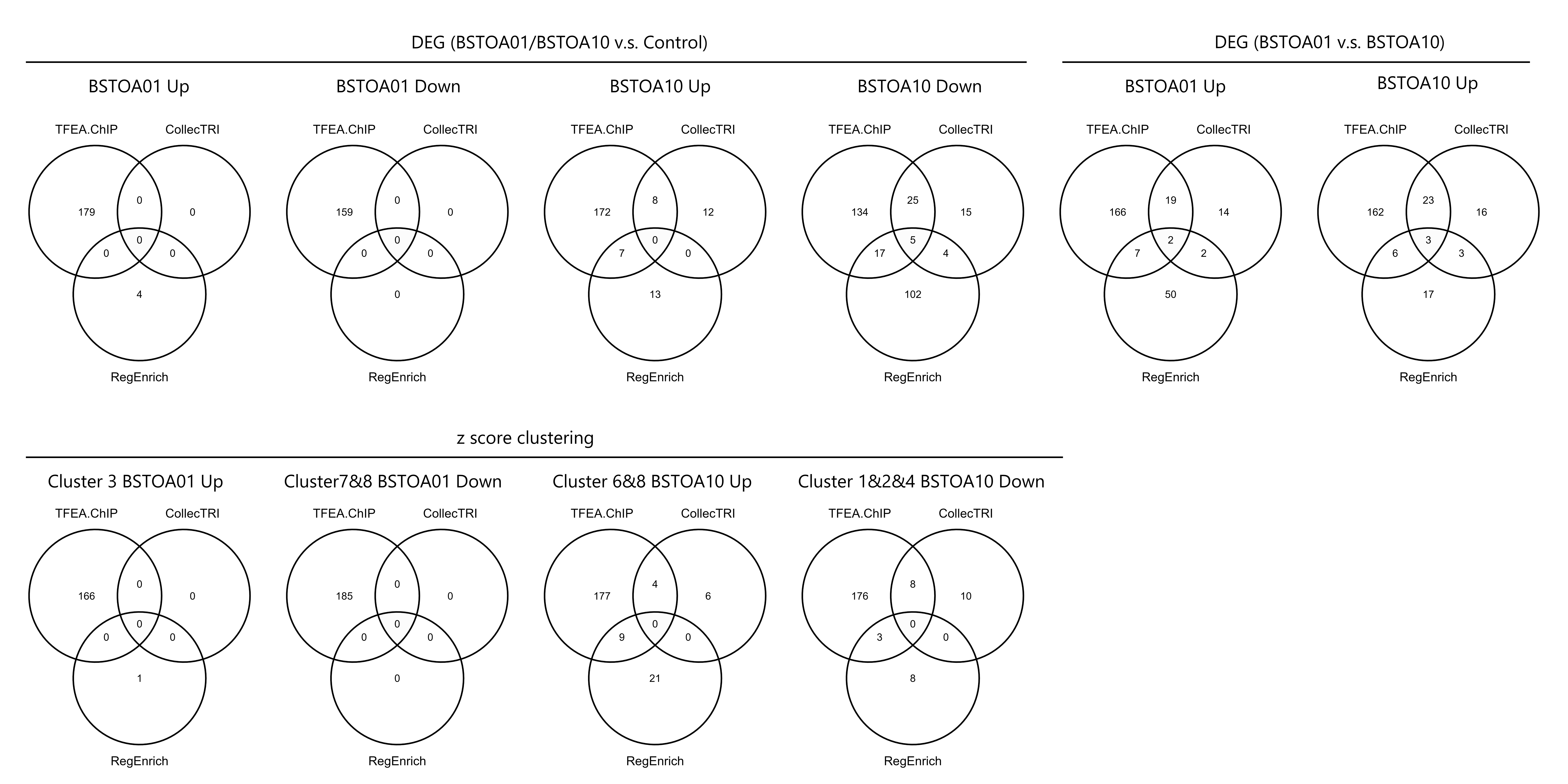

Supplement: Supplementary material — Supplementary Figure S1. An in vitro experimental system was established to investigate the effects of probiotics on NET formation. (a) Cell density during culture with (differentiation + ) or without (differentiation −) 1.25% (v/v) DMSO. Data are shown as raw value points with polynomial approximation curves (n > 3 for each day). (b) Parent HL-60 cells (upper panel) and 5-d differentiated nHL-60 cells (lower panel) were stained using May-Grünwald-Giemsa method and observed using standard bright-field microscopy. Black arrowheads indicate lobulated nuclei. Representative results from three independent experiments are shown. Scale bars: 50 µm. (c) 5-d differentiated nHL-60 cells were incubated with InvitrogenTM pHrodoTM Green E. coli BioParticlesTM for 1 h, and fluorescence was detected using standard flow cytometry (Cell Sorter SH800, Sony). Representative results from three independent experiments are shown. (d-g) Screening conditions for differentiation induction days and concentrations of NET inducers. NET levels were measured as mean fluorescent units (MFU) using a plate reader (Spark®, Tecan). (d, f) HL-60 cells incubated with 1.25% (v/v) DMSO for 4, 5, or 6 d were induced for NET formation using 100 nM PMA (d) or 5 mM CI (f). (e, g) 5-d differentiated nHL-60 cells were stimulated with 0.8–40 nM PMA (e) or 0.8–40 mM CI (g). Representative results from three independent experiments are shown. (h) 5-d differentiated nHL-60 cells were co-cultured with 100-fold LGG for 1 h, followed by NET formation induction with 100 nM PMA. Culture supernatants were collected 4 h after incubation. Data are shown as raw value points with mean bars (n = 6 for each condition). *p < 0.05, determined by the Wilcoxon signed-rank test. Supplementary Figure S2. The NET suppressive effect of BSTOA10 was observed when using CI as an inducer. nHL-60 cells were co-cultured with BSTOA at ratios (BSTOA/nHL-60 cells) of 0.0 (Control) or 0.1 (BSTOA01) (a), and 0.0 (Control) or 10 (BSTOA10) (b) f [file KGMR_A_2572788_SM7230.zip › Supplementary_Figures/FigureS8.tif]

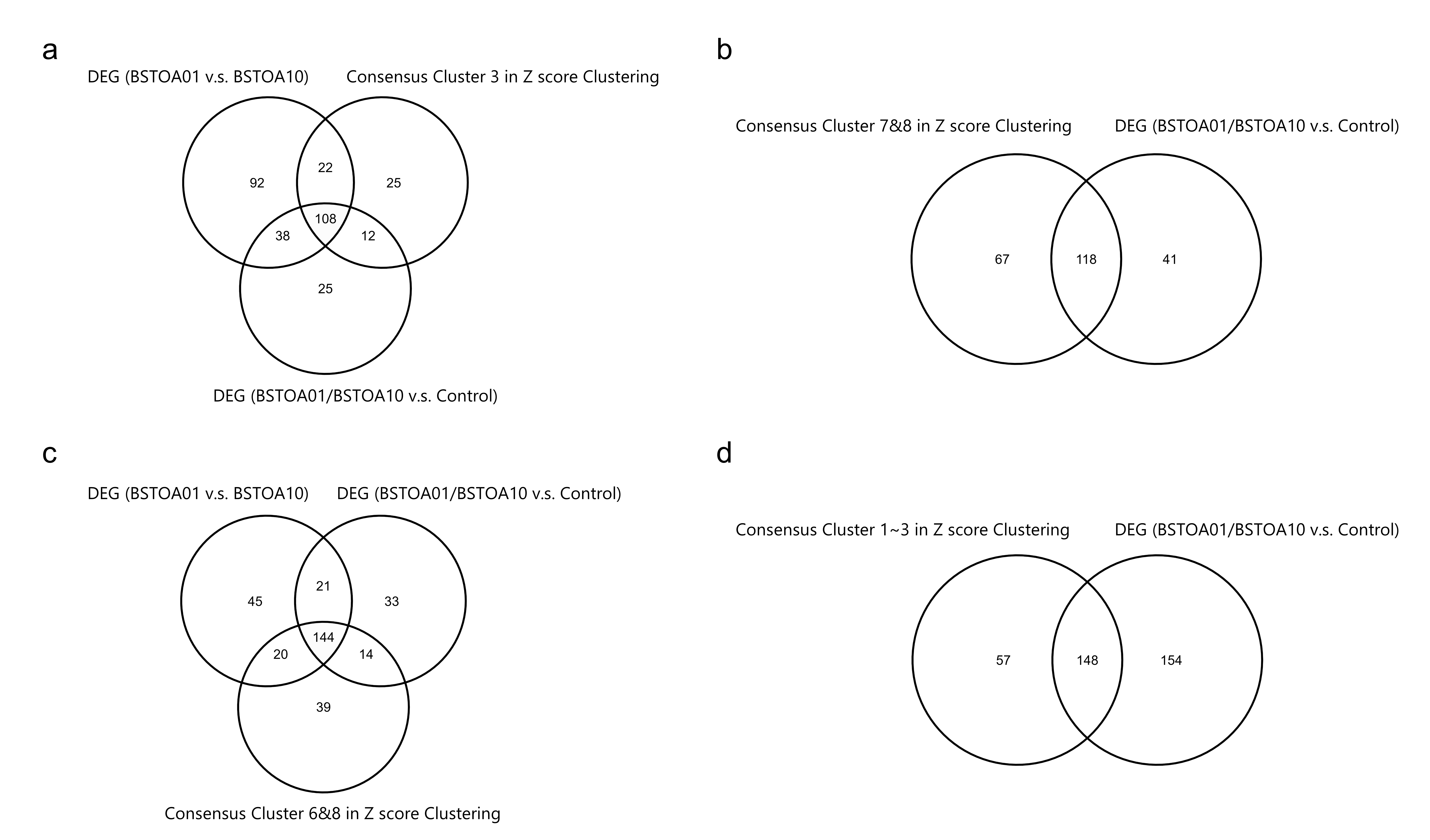

Supplement: Supplementary material — Supplementary Figure S1. An in vitro experimental system was established to investigate the effects of probiotics on NET formation. (a) Cell density during culture with (differentiation + ) or without (differentiation −) 1.25% (v/v) DMSO. Data are shown as raw value points with polynomial approximation curves (n > 3 for each day). (b) Parent HL-60 cells (upper panel) and 5-d differentiated nHL-60 cells (lower panel) were stained using May-Grünwald-Giemsa method and observed using standard bright-field microscopy. Black arrowheads indicate lobulated nuclei. Representative results from three independent experiments are shown. Scale bars: 50 µm. (c) 5-d differentiated nHL-60 cells were incubated with InvitrogenTM pHrodoTM Green E. coli BioParticlesTM for 1 h, and fluorescence was detected using standard flow cytometry (Cell Sorter SH800, Sony). Representative results from three independent experiments are shown. (d-g) Screening conditions for differentiation induction days and concentrations of NET inducers. NET levels were measured as mean fluorescent units (MFU) using a plate reader (Spark®, Tecan). (d, f) HL-60 cells incubated with 1.25% (v/v) DMSO for 4, 5, or 6 d were induced for NET formation using 100 nM PMA (d) or 5 mM CI (f). (e, g) 5-d differentiated nHL-60 cells were stimulated with 0.8–40 nM PMA (e) or 0.8–40 mM CI (g). Representative results from three independent experiments are shown. (h) 5-d differentiated nHL-60 cells were co-cultured with 100-fold LGG for 1 h, followed by NET formation induction with 100 nM PMA. Culture supernatants were collected 4 h after incubation. Data are shown as raw value points with mean bars (n = 6 for each condition). *p < 0.05, determined by the Wilcoxon signed-rank test. Supplementary Figure S2. The NET suppressive effect of BSTOA10 was observed when using CI as an inducer. nHL-60 cells were co-cultured with BSTOA at ratios (BSTOA/nHL-60 cells) of 0.0 (Control) or 0.1 (BSTOA01) (a), and 0.0 (Control) or 10 (BSTOA10) (b) f [file KGMR_A_2572788_SM7230.zip › Supplementary_Figures/FigureS9.tif]
