# Supplementary material for: Probiotic strain Bacillus subtilis TO-A modulates the formation of neutrophil extracellular traps
Source: Gut Microbes Rep. 2025 Oct 29;2(1):2572788. doi: 10.1080/29933935.2025.2572788 (PMC12940209; doi:10.1080/29933935.2025.2572788)
Supplement: Supplementary material — Supplementary Table S1. Differentially expressed genes in BSTOA01 and BSTOA10 compared with Control. Supplementary Table S2. Differentially expressed genes in BSTOA01 compared with BSTOA10. Supplementary Table S3. Genes in clusters showing z-score changes between BSTOA01 and BSTOA10. Supplementary Table S4. Enriched transcription factor genes in each cluster. Supplementary Table S5. List of excluded GO terms. [file KGMR_A_2572788_SM7233.docx]

**Supplementary Table S1.** Differentially expressed genes in BSTOA01 and BSTOA10 compared with Control.

| BSTOA01_Up | BSTOA01_Down | BSTOA10_Down | BSTOA10_Up | BSTOA01_Up &  BSTOA01_Down | BSTOA01_Up &  BSTOA10_Down | BSTOA01_Up &  BSTOA10_Up | BSTOA01_Down &  BSTOA10_Down | BSTOA01_Down &  BSTOA10_Up | BSTOA10_Down &  BSTOA10_Up |
| --- | --- | --- | --- | --- | --- | --- | --- | --- | --- |
| IVNS1ABP, GPR65, CLEC4E, HSPA5, PPP4R2, ADGRG3, ARRDC4, PSEN1, FBRS, ZDHHC18, DNAJB11, CD58, TLR2, HSPA1B, LACC1, GABARAPL1, C3, CHORDC1, MLLT6, SLC43A2, PDE3B, TMEM165, MAP3K11, SBNO2, SIRT1, GPATCH2L, ADGRE2, STK40, RASA2, CLEC6A, MID1IP1, HSP90B1, ZNF140, GALC, SNN, FMNL3, LONRF1, APBB3, EYA3, LIG4, RUFY1, FAM20B, GPR75 | CDC42EP3, CNPY3, VAPA, RP11-380G5.2, RABGGTB, CYTIP, SNRNP48, ZSWIM1, TNFRSF12A, CYBRD1, KLHL21, PIGU, CCNJ, GPCPD1, MXD4, PIM1, TSNAX, ZRSR2, RP5-1091N2.9, RBM45, CLEC2B, MRPL36, SCAF11, PPM1B | TXNIP, MSRB1, PXK, CCNG2, MEGF9, KIAA1551, CASP2, RNF24, TMEM55A, SSH2, SNX27, UBR2, ZYG11B, FBXW2, LDB1, MCU, DGKD, TLR8, NFATC3, GIT2, STK38, PIK3CG, ENTPD1, IL17RA, RNF38, GCA, ANKRD22, NLRP12, RBM38, NT5C2, PPP2R5A, ZFP36L2, ENTPD7, PARP8, HHEX, DCAF10, NKTR, GAPT, TRAFD1, VNN2, KIAA0513, CA5B, FYB, ORC5, TGFBR2, ERCC5, SP1, CAMKK2, GLRX, CCPG1, PGD, CEBPE, ATM, ZNF490, OGFRL1, DIRC2, CNOT8, STAG3L3, PHC3, SLC8B1, CTNND1, FAM214B, CCDC125, LYST, RPS6KA1, FLI1, WASL, RSBN1L, NUDT4, KIAA0930, TSC1, ARHGAP1, TRIM33, RICTOR, THRA, GPATCH8, PRAME, FGD3, PIKFYVE, SIRPB1, SUPT20H, FOXJ3, NUDT4P1, STAG3L1, MXI1, ZNF283, SLC22A15, RAB3D, MYO1F, SMAD4, DUSP11, AMICA1, NLRC4, RPE, SLC19A1, GTF2H2C_2, RAB18, CTDSP2, ANGEL2, GRK6, SFXN5, SETDB1, HJURP, ITGAL, DENND6A, HSPBAP1, STAG3L2, SORT1, ARID4A, WIPF1, TAF8, ZNF224, UBXN2B, TAS2R31, GTF2I, DTX4, MSL1, MTMR10, AKTIP, WDR33, CREBRF, WIPI1, MFSD14B, MAP3K1, SYNRG, ZNF107, NSF, STEAP4, SLC25A40, CBWD5, AFF1, GATAD2B, PLK1, SH3BP2, FAM160A2, FHL3, C1orf162, OSBPL11, CIR1, ZDHHC7, MAP1S, TGFBR1, AGO4, SMC3, RAB11FIP1, ATG14, HVCN1, TM9SF2, MDM4, MAPK14, ZNF585B, HSH2D, ABHD2, WIPI2, APAF1, LY75-CD302, KIAA0319L, MED23, GGNBP2, BRD8, DCP1B, IFI16, SEMA4A, ZNF461, WBP1L, PIK3CB, S100PBP, AIF1, SAMD9, FAM175B, GLCCI1, ZNF592, KIAA1033, SESN3, NSUN5, ARHGAP9, DPF2, ATMIN, SLC35A1, ADRBK1, MKRN1, PIAS1, GYG1, TBC1D14, SETD1B, TNPO3, CLK2, LRRC37A, AP3S2, PHC2, CDA, ZZZ3, CDK13, FIG4, TADA2B, MBD4, ZXDC, DAGLB, DOPEY2, CIRBP, RNF111, ZNF106, LPCAT2, PTPN22, UBN1, HIPK2, AASDHPPT, FAM45A, CLEC12A, ZNF852, CBWD2, TRIM14, VPS29, IKZF1, AKNA, ADSS, TNRC6A, EIF4EBP2, ATP11A, MTO1, ZNF652, GUSBP2, ORAI2, ZADH2, LRRC37A3, CHMP3, SPAST, OGFR, ZNF740, PTAR1, WHSC1L1, ZNF611, LBR, STK4, CHD8, ADAR, CCDC66, VAPB, IREB2, MGME1, GDAP2, PHF21A, PIK3R1, RNF220, ASXL2, XRCC5, MTIF3, PRKAB1, TMOD2, MRPS18B, TRAPPC12, LRRC37B, DOK3, MTMR4, ZNF451, LCORL, ZBTB7B, MFSD14C, MGAT4A, CEP295, TM6SF1, CDC40, ZNF35, ULK1, RIC1, AGPS, CRBN, TMEM154, C10orf131, CRLF3, ZNF141, NAA60, SLC20A1, LRRK2, MAX, CD300LF, CD47, CGGBP1, CCNDBP1, UBE4B, FTO, GLTSCR1L, TRAPPC11, PRH1, GOLGA5, OXA1L, ZNF280D, KIAA0430, STIM2, IRAK4, PARP4, RNF114, LASP1, ITFG2, VCPIP1, GPR34, QTRTD1, SKP2, CEP192, NDUFV3, SIGLEC14, POMK, ZNF718, CLTCL1, TBC1D2B, ZMYM6NB, DYNC1LI2, LRRC37A2, RNF138, SEL1L, MIDN, ZNF148, PEX11B, DPP8, CTDP1, BAG4, POLI, VDR, DCUN1D2, MBP, FAM134A, SPTLC2, SEPSECS, KIAA1468, BROX, CLINT1, SLC40A1, CUTC, KPNA6, DMTF1, BRCC3, DCAF5, KIAA1586, FBXL20, NRDE2, KDM1B, SUCNR1, ZNF845, CBL, ATXN2, RRAGD, SLC25A38, PML, ZNF445, SIRPD, RPRD2, FKBP15, SYVN1, TMEM260, PPIP5K2, NHLRC3, ATF7, ANLN, RBM41, ARHGAP11B, SS18, THEMIS2, SMG7, PKN2, PIK3C3, APPBP2, EXOC1, DHX8, BRWD1, ZNF767P, MFAP3, LOC643802, ZMYM4, ZNF761, RCOR3, TAS2R30, SMAD5, TMX3, PCMTD1, ZNF253, NUPL2, ELF2, GMIP, IFNAR2, RAF1, ASH1L, SNX13, FAM101B, TNRC6B, POLK, EP400, NCOA3, SNX20, NCK1, PHIP, KCTD5, P2RY8, ATP2A3, RCBTB2, MAPKAPK3, VPS39, SHPRH, C15orf38-AP3S2, PAN3, ASF1A, AGAP9, MCMBP, TUBA1A, EAPP, CBWD6, ARHGEF18, WAS, CMTM7, CYSLTR1, FARSB, CDC16, NPEPPS, MGA, NAPB, ZNF302, LATS1, KLHL18, NDE1, TM7SF3, SACM1L, MSL3, MPEG1, PANK2, SMAD2, TMEM11, NEK6, ZMYND8, TMLHE, SUOX, SCYL3, SYK, DCLRE1C, BORA, CARD8, NDST2, TMEM87A, NSD1, MANBA, CUL4A, TNFAIP8, GALNT7, SAP130, AMPD3, SNX2, CRTAP, STX7, GNPAT, ACVR1B, C10orf54, BRAP, NUDT3, PLAG1, SMIM12, C2CD5, SLC9A6, TIA1, TREML2, MANSC1, DGCR2, SP110, CTBS, CKAP2, NHLRC2, AGAP6, FAM160B1, EHMT1, COX15, CEP63, SPTBN1, AAR2, BIVM-ERCC5, COPS7B, CAMK2G, RMI1, MAU2, TAS2R14, GCLC, ADD3, R3HDM1, CCDC88C, RAP2B, ZNF850, APBB1IP, CD101, KAT7, IPO9, DNAJC16, ZNF567, ZNF226, DGCR14, FBXO38, NFRKB, FAM214A, ZC2HC1A, CCDC6, NR2C1, ACAP2, CLEC2D, FAM73A, MTM1, MCM9, ASH2L, RTF1, RASGRP4, RAB11FIP4, FOXM1, SLX4IP, EMSY, ZNF701, RUNX2, NOA1, CASC3, SLC15A4, ARHGAP30, POGK, C5orf22, SLC4A1AP, SLC36A1, AP3M1, FAM105A, MTIF2, COL4A3BP, GMPR2, HEATR5A, ANKZF1, ZFP62, APPL1, ACBD5, PRR4, SF1, CXorf38, MRPL44, CBWD1, P2RY2, RNF7, ATXN7, CEP104, SRSF4, PPP1R9B, AVL9, RERE, TPP2, RUFY2, GPD2, DCTN5, NPL, CNPPD1, DBP, C7orf49, GTPBP10, MAT2B, SOS1, ZNF613, LRRC47, COG3, ZNF41, FAM53B, FAM117A, FBXL4, MADD, FAM63A, SOCS5, KPNA3, ASNSD1, RNF103-CHMP3, RBM12B, HMGCL, VPS8, E2F8, BCL2L13, GORAB, BLCAP, FOXK2, GUF1, FBXO8, PMS2, MKNK1, KDM4A, ATAD5, FAM208A, TRIM21, LAMTOR1, STX6, SHISA5, APC, LRRC58, MSL2, PHF20, CORO2A, TP53INP1, CTAGE5, TTK, SETDB2, GPR21, ZC3H14, CRLS1, ZNF708, TTI1, ZNF780B, CWF19L1, NDUFA10, THUMPD1, TMEM57, RNF14, REST, TAF5, RBM33, ABHD18, KIAA1429, LAPTM4A, MINA, RCHY1, DAPK2, SLC25A20, HNRNPA1L2, ZNF417, TBP, AGAP5, TXNL4B, XPC, DEF6, TCHP, TRMT10B, POC5, ZER1, GDE1, ZMYM5, PLEKHO1, FAM179B, CYB5R4, CABIN1, DPY19L4, PDPR, P2RY10, HACL1, CTCF, GATS, RALGAPB, RFX7, ZBTB44, DOPEY1, TOP3B, ANKRD49, BLOC1S5, HERC2P3, NMT1, AGAP4, SLC25A46, LINS1, ELMO2, SETD2, CHRFAM7A, GLT8D1, NUDCD3, NARF, ANAPC16, RIPK3, ZNF101, ZNF17, HMG20A, LDAH, CPT2, PPP1R21, ARMC1, ZDHHC3, DSTYK, FAM199X, CAPNS2, MIA3, PDHB, CWF19L2, GSE1, ZNF587, NFIC, RYK, UPF2, NMNAT1, ATG16L2, PIAS2, ZNF791, GP1BB, FAM122A, ZNF518A, TBCC, VTA1, RAD52, VPS33A, CHAMP1, PRRC1, INTS12, CXXC1, ABCB10, BPNT1, VPS33B, KLHL20, LARP4B, MBD6, VAT1, GPSM2, CCDC186, ICE2, CEP44, TGDS, ACAD10, EIF4ENIF1, GBF1, GOLPH3L, STX10, ORAI3, SUDS3, ERP27, CCDC122, NLRC5, TMEM243, NAA16, SASH3, ZNF91, NIF3L1, MGAT1, RMND1, C19orf47, SDAD1, VPS26B, KAT6B, STAM, CHTF8, ANKMY1, STAMBPL1, NSL1, ZBTB40, ZNF700, CTR9, C8orf44, SSH1, WDR77, ALG11, OARD1, PCMTD2, MCPH1, WDFY2, SCRN3, MKRN2, ANKMY2, DFFA, NBEAL2, YPEL3, LRRC28, ZNF384, LIX1L, JAK2, SASS6, SAMD9L, TBC1D13, NUP85, PPWD1, NUP93, ZNF397, PHKG2, IRF2, MTR, CLCC1, RNF169, THUMPD3, IFFO1, MRRF, PSMB8-AS1, BLOC1S2, HPS4, ZNF235, ZNF208, CRNKL1, OTUD6B, GOLGA8K, RXRB, QRSL1, VIPAS39, RAB11FIP2, GLE1, SLC33A1, MTFR1L, NAA30, ASUN, FBXO5, FAM117B, NXPE3, XPO7, DHODH, ANAPC7, MRPL35, ZNF721, KMT2A, FAN1, RNF146, MAP2K5, CRTC3, ZNF585A, ACIN1, CNOT6, TAS2R20, MSANTD4, NOL9, METTL15, PAPD5, VPS54, GNG2, BPGM, FPGT-TNNI3K, SRPK2, ZNF350, NPHP3-ACAD11, METAP1, MAPKAP1, RBBP5, C16orf58, SNX5, RUBCN, NISCH, GTF2A1, GOLGA1, GATAD1, PRDM10, OSER1, MRPL34, PALB2, KBTBD11, TDP2, COMMD9, GRAMD4, PURA, CEACAM3, NUP210, RIPK1, BCL11A, TRMT1L, METTL3, KIF15, C9orf91, SPNS3, GGPS1, SLF2, SLC22A16, MAVS, SREK1, ITPKB, VTI1B, ZNF431, RCBTB1, CKLF, SLC30A6, PAPOLG, TNFSF13B, INCENP, PGRMC2, PIK3R6, METTL22, GTF2E2, AKAP10, ETV5, ZNF182, ZNF609, TMEM63A, ARHGEF1, TACO1, ZNF382, ZNF28, KDSR, EXOC6, GANC, ZNF230, ZDHHC16, ANKS1A, SLC15A2, TRIM4, MTFMT, BORCS8, WDR37, ZBED5, MTMR9, RAB43, TOLLIP, ANKRD13C, DHX35, TCF20, PVRL1, MBTD1, ZDHHC17, C11orf54, HELQ, ZNF658B, PHAX, NEMF, SENP7, C12orf65, LOC646938, KIAA0232, FTSJ2, ARHGAP12, GOPC, SGSH, U2AF2, NELFA, DDX56, PUS7L, WDSUB1, ATP13A1, TMEM214, IPO8, LRRC41, TMEM41A, HECTD3, NDUFAF5, ZNF8, CREBZF, FAF2, CASP8AP2, THAP6, MANEA, ZNF260, C17orf80, AGGF1, TRIP11, KRBOX4, ERLIN2, SLC48A1, TNKS, ARV1, KCTD2, TMTC4, KANSL1L, PRRC2B, SUV39H1, GTF3C2, FAM135A, VPS53, SLC22A4, NCK2, PTTG2, PCYOX1, KEAP1, RPAP2, FAM193B, KANSL3, ZNF75A, ZNF512, TMEM19, ZNF138, ZNF507, ATXN7L3B, PARP16, RNPEPL1, MTG2, ZNF254, PRDM4, TRIM59, KLHDC10, ZNF439, CDC6, ERCC6L2, MPHOSPH10, DSN1, ASB16-AS1, BTBD3, INAFM2, ALS2, NCOA5, P2RX1, B3GNT2, AGL, DGKG, PIGBOS1, USP21, METTL17, ZNF468, MRPL49, ZNF564, FUT10, PYGO2, MEN1, MED18, SDHAF2, COX11, BIVM, MRPS16, DYNLT1, DHRSX, TMEM216, FEZ2, TRIP4, TMEM168, IFT80, PDCD7, PHOSPHO1, COG2, ICMT, NPHP3, RGS14, ZNF3, PHTF1, DNAJC17, PRMT3, XKR8, TPCN2, PSD4, PIGM, NCOA6, KIN, PRMT2, PLAGL1, TMEM167A, ZNF790, STAT2, COX18, DNMBP, FAM206A, FYCO1, ELMOD3, TYW5, NR2C2, DTX2, TIAL1, ZNF440, DCAF17, DTX3L, PRKACB, SMUG1, UHRF1BP1, PPP1R8, COG1, ACOX1, ZNF432, EPT1, TPTE, OGG1, JADE1, GTF2E1, ING4, DESI2 | CSF1, TRIM25, MAP4K4, YPEL5, PROK2, STK38L, SLC3A2, FNIP2, PRNP, PAXBP1, SEC24A, STOM, H3F3B, PPTC7, SLC2A14, TMEM14EP, SNX9, ZNFX1, CYCS, RAB7B, RCAN1, CYTH3, DUSP6, AHR, BHLHE40, ATP2C1, TNFSF14, PNRC2, CRKL, LYRM1, ANKRD28, PTGER4, HNRNPC, RLF, OTUD4, NUS1, IMP3, TIPARP, RANBP9, PHLPP1, ZBTB43, SQLE, ANXA5, GNA13, ATXN1, PTTG1IP, DERL1, MTHFD2, PGAP1, PTPN12, TOMM34, TNFRSF10B, DAPP1, LINC01578, CDK17, GRPEL1, QKI, SEC22B, NSMAF, TBK1, SAP30BP, BCL2L11, PIK3AP1, TCEB1, S100A10, SLC7A1, MPP1, ATP1A1, RGPD5, RAB9A, THAP9-AS1, SLC1A5, LAMTOR3, ENSA, UBAC2, ANKRD10, HAUS2, WSB1, PPP2CA, CRELD2, CTNNB1, RPF2, LMNA, ATF4, UBAP1, STX4, LOC81691, SLA, C16orf72, EIF1B, SLC2A1, C15orf39, CHMP4B, TFRC, SERPINA1, PTPMT1, SLC43A3, C5AR2, GLA, CCL1, CKS2, PRKAG2, ERN1, POMP, ECE1, DNAJB9, NEU1, SUPT6H, PTAFR, LPL, SMAD3, FBXO11, NDUFAF6, SLC25A33, TWISTNB, AKIRIN1, NBPF12, HK3, SPRED2, TERF2IP, RRAGA, SNUPN, LONRF3, DIABLO, GTPBP4, TPRA1, BASP1, PPP1R11, IL13RA1, ADORA2A, HMGCR, BMP6, GLUL, DOCK10, BCORL1, TMEM184C, CCDC9, VAMP1, IL3RA, DDX26B, SLPI, NOCT, CKAP2L, BNIP3, DNAJC1, PSMA1, UHRF1BP1L, LYSMD3, MIS18A, ZFP36L1, HCST, TAF13, NPC1, BCL2L1, ELF1, UBE2D1, RANBP2, ADNP2, REPIN1, ANKRD42, GSTO1, TOP1, PLPP6, HIST2H3A, NFAT5, BCAS2, AEN, PMPCA, TRIB3, HSPA13, AGAP3, P2RX4, TNFRSF9, RNF168, ZNF706, TMPO, CYP51A1, RAB12, PTS, RHEB, PDK1, NR1H2, SLC35F2, GNA15, PRDX1, CCDC88A, EIF4A3, S100P, PRPF39, CLN8, TGFBRAP1, TLE3, WBP4, TAF4B, H3F3C, PRMT1, FPGS, ZBTB1, FBXO30, SLC12A7, ARFGAP3, B4GALT1, BEX1, RGS10, SRA1, KCNK5, PI4K2B, ESYT2, MSMO1, YIPF6, CDK16, MTHFD1L, ELMSAN1, KIF1B, CDK7, FBXW11, CRLF2, NKIRAS1, WSB2, AAED1, MED30, GXYLT2, MED29, SMOX, DDIT3, INIP, SEC61B, CEBPB, ATP6V1G3, ATXN2L, SLC16A1, LUZP1, MRPS24, INHBA, SESTD1, POLA1, RGPD4, WDR91, NBPF8, PDP1, NOP16, KLF2, IARS, STX1A, RIBC1, RDH13, WWC1, RGPD3, PPP6R3, EID3, RBBP8, SLC26A2, ZNF331, GPR18, RCC2 | N. D. | N. D. | CCL3, PTGS2, CCL3L3, EGR3, G0S2, SOD2, NR4A1, DUSP2, NFKBIA, NFKBID, PLAUR, TNFAIP6, TNFAIP3, OLR1, ARL5B, EGR1, NFKBIZ, F3, PPP1R15A, ZC3H12A, GADD45B, MAP3K8, NABP1, ICAM1, CA2, DDIT4, PDE4B, MFSD2A, CDKN1A, RASGEF1B, REL, BTG2, CCL5, KDM6B, HCAR3, SPP1, JUNB, PPP1R15B, CCL20, HIVEP1, NFE2L2, IER5, GBP2, BIRC3, CCRL2, ACSL1, NFIL3, GK, ARRDC3, MCL1, ETS2, GPR84, CXCL3, TNF, TNFAIP2, KCNJ2, C15orf48, PTGER2, SAT1, IL1R2, ATP2B1, RABGEF1, CCL18, NBN, ZBTB10, TIFA, NCOA7, CCL2, PIM2, DCUN1D3, SPRY1, DUSP16, SIAH2, IRAK2, HCAR2, PHLDA1, KIAA0226L, MAFB, SAMSN1, PNPLA8, TREM1, TRIB1, OSM, MARCKS, HIVEP2, ZFP36, RGS2, SLC1A3, MIOS, GCH1, ABCA1, CSRNP1, EBLN2, PFKFB3, AQP9, RNF144B, NAMPT, NFKB2, SERPINE1, PLEK, BTG3, HIF1A, SLC11A2, FTH1, PTGES, TSC22D2, ANKRD36, NCR3LG1, CD55, LDLR, PLK3, FOSL2, FOSL1, DUSP1, NBPF19, PMAIP1, IER2, IL2RG, NOTCH2NL, TANK, MCTP1, GPR132, AREG, MXD1, BCL2A1, IFNGR2, DSE, GK5, SRGN, NAB1, DDX3X, ITPRIP, CD69, DNAJA1, ZBTB21, SKIL, LCP2, RNF19B, N4BP1, PIM3, TPM4, UGCG, C5AR1, CD44, SDC4, NFKBIE, IGSF6, SLC39A8, BCL3, ETF1, NFKB1, CXCL2, ADM, PLEKHF2, SH3BP5, SPAG9, CHD2, CHST11, BID, THBS1, RILPL2, TNIP1, KLF10, CCNL1, FCAR, ATF3, NOTCH2, MTF1, IL10RA, EHD4, RGS1, FILIP1L, RHOH, ZNF267, EHD1, C3AR1, RAB8B, CLIC4, TMEM185B, CD82, RELB, MAPK6, SP140, HBEGF, MMP8, FOS, ANKRD36B, TAGAP, USP12, TRAF1, POR, IL18, TRIP10, KLHL15, NBPF14, TP53BP2, TIMP1, EPM2AIP1, PTX3, LAMB3, SLC16A14, WTAP, PLAGL2, JUN, CELF1, SGMS2, GTPBP1, EAF1, DRAM1, SRC, ADAMTSL4-AS1, FUNDC2, DENND5A, TRGJ1, YRDC, BATF3, IL1RAP, RAPGEF2, SGK1, GJB2, RIF1, FFAR2, GABARAPL2, EGR2, SLAMF7, HIP1, CHD1, RAB21, RALGDS, TBC1D7, NBPF10, DOT1L, DYNLT3, RGPD2, TMEM41B, CPEB4, FAM107B, MAP2K3, FAM53C, RIPK2, AGO2, SERPINB2, LIMK2, LIF, SATB1, PPARG, OXSR1, CCL24, TRAF3, FAM46C, HSPA4L, IL1RN, KBTBD2, SLC16A3, PHF20L1, TOM1, NECAP1, NINJ1, KMO, MSC, TGIF2, NBPF26, GPR35, PRDM1, ENPP2, EDN1, BZW1, NR4A3, C4orf46, SMPD2, MDFIC, IL1A, SUSD6, PITPNB, SPECC1L-ADORA2A, NIPA1, ADAM17, CCNT1, UBE2E1, LPCAT1, PI3, DUSP5, CD48, TMEM52B, SNAPC1, ISG20L2, SLC2A3, CXCL16, NBPF20, ELOVL7, XBP1, SRI, CLCF1, NAF1, PPP1R10, SBDS, TRA2B, SPTY2D1, ABCF1, VOPP1, METRNL, MIER1, FLOT1, PLIN2, CLDND1, INSIG1, CCDC82, STARD3NL, BIRC2, RGPD6, ETV3, ABL2, ZFX, TFEC, SERPINB9, MYD88, PHACTR1, PNPLA1, LYRM4, JMJD6, FOSB, TTL, CCL4L2, IL1B, CXorf65, GABPB1, UBE2E2, HRH4, UAP1, DDX60L, ATP13A3, ACOT9, SH2B3, LRRC8B, SDE2, RFX5, NLRP3, SUMO4, RGPD1, NBPF9, CD83, NDUFV2, LAMP3, IER3, TGIF1, URGCP, PITPNA, RGPD8, SYAP1, RGCC, ANKLE2, VEGFA, LAX1, USP13, HMGCS1, CAMK1G, IL12B, ATP1B3, RYBP, FSCN1, FAM177A1, VPS37B, IL1R1, LIPN, FZD1, RNF19A, ST7L, TMEM120A, PIGA, KIR2DL4, IFIH1, CXCL8, FAS, LIMS3L, DDX21, DUSP10, CHD4, RAB20, CREB3, POGZ, MAFF, JUND, USP36, JOSD1, CD38, SLC7A5, HIST1H4A, TMEM88, SYNJ1, PLAU, HNRNPCL1, TNFSF9, NR4A2, TRIM39, B3GNT5, LPXN, ZFY, RIOK1, BTN2A2, SELK, CCNE2, ADAT1, IL23A, DUSP3, TSPYL2, NUP188, PPIF, CREM, GPR183, FLT1, ASB6 | ARHGAP25, HIPK1, CASP8, FAM46A, RGS18, TUBA4A, ZNF217, SIRPB2, MYB, CAT, LOC400927, MIS18BP1, CLPX, TLR1, CELF2, CCM2, LMO2, ADRB2, USP3, SERTAD2, PADI4, ZNF770, B3GNT8, SLC7A6OS, PHF3, F8A2, NFE2, SELPLG, HEATR6, MAN2A2, FCGR3B, TNFRSF10C, PRAM1, INPP5D, SLC37A3, PPCS, ARL17B, FAM217B, NUP50, ACAD8, ZNF776, 45717, GTF2H2, ASB8, C16orf54, PPP1R3B, LRMP, LYPLAL1, POLG2, ZNF780A, MLXIP, F8A3, RBM26, C16orf70, PLGLB1, ZBTB34, PHF12, ZC3HAV1, KLF3, COA5, CYTH4, RNF34, SLC35E2B, LPAR6, NT5C3A, MRFAP1L1, GTF2IRD2, RNF125, NDST1, CXCR2, LSM6, SLC35E2, UHRF2, ZNF766, CNR2, WDR5B, ELP2, CHD9, SLC9A3R1, ACPP, RNPC3, IDH1, TNFSF10, C6orf136, DDX28, ZNF419, HDAC4, PINK1, KCNE3, TSC22D3, MYC, IKZF5, CEBPA, MRPS30, MFSD8, RUNDC1, C9orf85, CDKN2AIP, UIMC1, MPPE1, MBIP, KLHL24, ITGA4, RSBN1, IL16, RSC1A1, FAM65B, TOB1, SLC25A36, FES, ACSS3, ZNF383, CNEP1R1, FADD, HRH2, PGPEP1, CIDEB, CEPT1, MCAT, FPGT, FAM185A, TRIM23, DUS2, ZNF398, TLR6, C1QTNF3-AMACR, TRIM27, SIGLEC9, CHPT1, FGFR1OP, OR52K2, AP4B1, C7orf25, TTC9C, C3orf38, PDF, ASB7, GLRX2, FRAT1, MKKS, EVI5, FOLR3, PCM1, ATG16L1, ABHD17B, ZNF227, CCDC84, ANKRD55, DTWD1, ZFP30, ISL2, PDIK1L, TM2D2, NAIP, WDR53, BTN3A2, CMTM4, FAM134C, ZNF223, ZBTB18, CD46, NME6, FANCF, ZNF746, ZBTB41, C20orf197, GIN1, EXOSC3, CMTM1, VPS11, HINFP, ZNF557, ABTB1, AMACR, ADCY7, CARD6, MTERF1, GFI1, CBR4, FEM1C, FKBP9, SMIM4, POLA2, BBS10, DCAF12, RAB33B, PARD6B, OR52K1, PIGG | N. D. | SCAP |

**Supplementary Table S2.** Differentially expressed genes in BSTOA01 compared with BSTOA10.

| BSTOA01 > BSTOA10 | BSTOA01 < BSTOA10 |
| --- | --- |
| GPR155, TUBA4A, ATAD5, SYK, TTI1, AVL9, CHPT1, SIRPB1, KDM1B, MAX, PML, PHIP, TSPAN2, C10orf54, ZNF780A, VPRBP, CCPG1, NME6, BPGM, ATM, ABTB1, NR6A1, UPF2, RAB18, POLI, NAIP, RASGRP4, SREK1, VCPIP1, MTMR10, CLIP1, ITFG2, CLEC6A, MAU2, ENPP4, MGAT4A, DGCR8, MGA, P2RY8, P2RY8, ZNF611, ZNF740, GPSM2, KPNA6, FOLR3, TMEM154, TCF20, TCF20, SRSF4, ZNF107, CIR1, MAK16, UBE4B, GLTSCR1L, BCL2L13, ZNF398, DCTN5, IL17RA, SLC8B1, FAF2, ARHGAP9, RAB11FIP4, TMLHE, HIPK2, ORAI2, SMG7, KAT7, ZDHHC3, ACBD5, PTPN9, C16orf58, MFSD8, ZER1, TBCK, ZNF407, GOLGA5, SENP2, HSPH1, SLC25A38, FAM63A, NFRKB, SLC25A40, ELMOD3, TAF8, GPR21, SMG6, FLI1, ITGAL, SLC9A3R1, DIRC2, CORO2A, MANSC1, RNF24, CAMKK2, SLC15A2, IRAK4, TNRC6B, RGS18, LOC400927, FBXO8, PCMTD2, KIAA0930, KAT6A, SLC35A5, ULK1, SPTLC2, PXK, FAM193B, ARHGAP18, PARP8, MYLIP, TBC1D2B, GTF2H2, WDR33, GLRX, NCOA5, FAM134B, ZNF17, ZMYM4, NR2C2, CLOCK, ZNF75D, DHRSX, RPS6KA1, RNF38, FBXW7, FAM160B1, ATF7, ABHD17B, LRMP, SP2, COL4A3BP, CIDEB, CARD16, LONRF1, MTFR1L, FAM122A, SSH2, HVCN1, DAPK2, DOPEY2, OGFR, PHAX, THRA, MSL1, PIK3CG, GPR34, RBM41, FAM117A, CEP295, ZNF382, MANSC1, MFSD6, NBN, ZFYVE16, ZNF592, ATG16L2, NDST1, MBD4, VCPKMT, QTRTD1, ZNF283, CDK13, NXPE3, ARL17B, ICE2, PDIK1L, PADI4, TOB1, FAM53B, TRMT10B, IKZF1, ENTPD7, PRKAB1, RPS6KA1, SPAST, RPS6KA1, DESI2, HRH2, STK38, FOXP1, ARL17B, ASXL2, ITPKB, ARHGAP25, EIF4ENIF1, FBXW2, MBIP, 45724, POMK, FAM199X, CWF19L2, DCUN1D2, GCLC, FBXO38, RICTOR, BAG4, RAF1, RFX7, ZNF106, EPB41, MAP2K5, RBM26, NKTR, POLK, TM6SF1, PGD, SUSD1, LRRC28, PHC2, GIT2, IDH1, DTX4, PARP1, NCOA3, PLEKHO1, PRDM10, RAB3D, TMEM167A, SNX20, SUDS3, CEACAM3, SETD1B, FUBP3, FAM105A, ZNF780A, ATG14, CHD8, BLCAP, GORAB, TAB3, IVNS1ABP, SEMA4A, TBC1D14, ZNF770, FGD3, CEP44, MOB3C, POGK, WIPI1, ZNF780A, DNAJC16, ESCO1, ZNF445, PHTF1, CCDC125, RPS6KA1, APPBP2, PPP1R21, ADGRE1, SLC35A1, PRMT2, GBA2, TACC1, ZMYM6NB, RASSF2, MDM4, TADA2B, LINS1, EP400, FAM134A, ZNF609, IREB2, HSP90B1, ZNF845, TMEM214, ZZZ3, FTO, CWF19L1, RIPK3, RCOR3, MPEG1, HSH2D, PIKFYVE, B3GNT2, NAP1L1, TRAPPC11, WASL, FBXL4, ARHGEF1, GATAD2B, LTF, RNF169, DGCR2, PHF3, ARAP2, MCU, TLR8, LDB1, TAS2R30, TSC1, BRD8, ASH1L, NBEAL2, ACAD10, TAS2R14, ZNF302, PAG1, SNX27, MED23, SKP2, LDAH, ANKRD13C, KIAA1551, ZNF767P, KIAA0232, CPT2, NCK1, FOXJ3, CTSH, SEC16A, UBR2, APC, RAB43, ORC5, E2F8, HEATR5A, SELPLG, ALG11, DGKD, SCYL3, ZNF721, ACSS3, SEPSECS, SYNRG, BTBD3, GPATCH8, CLEC4D, BORA, MSL3, ATMIN, MAP3K1, CRLF3, ZNF91, DUSP11, AKTIP, RPS6KA1, ARMT1, LRRK2, MGAT1, GCNT1, HDAC4, NCOA6, DCAF5, TAS2R31, FAM134C, RIC1, CEBPA, ZNF852, MYB, ZMYND8, GAPT, ZC2HC1A, PEX11B, KLHL20, ZFP30, ARHGAP1, RCBTB2, ACPP, RSBN1, FCGR3B, RNF34, RNF111, RMI1, SLC25A20, S100PBP, ANGEL2, TLR1, FOS, ZNF567, U2AF2, NSF, CDKN2AIP, KDM4A, CASP2, GALNT7, PANK2, C20orf197, FHL3, TRAFD1, ATXN2, ZNF35, SLC19A1, INAFM2, CA5B, ZBTB7B, SASS6, HMGCL, IL16, RSBN1L, GTF2E1, HSPBAP1, 45717, ZNF227, WBP1L, ZNF780B, ZNF780B, NRDE2, VPS11, ZNF490, CCDC66, SAP130, TAS2R20, PADI4, NPHP3, OSBPL11, MTMR4, OR52K1, TRIP11, TRIM33, GLCCI1, CEBPE, SASH3, NFATC3, STEAP4, BRCC3, CELF2, ARRDC4, AP4B1, JAK2, NLRC4, PTAR1, ZNF223, ZNF224, MTERF1, CCNG2, CTNND1, DGKD, SIGLEC9, MAP1S, INPP5D, RBM38, MSRB1, ZNF780A, RTF1, NLRP12, CAPNS2, KIAA0430, INPP5D, NDST2, DCP1B, TGDS, KIAA0226L, PRR4, HINFP, GIN1, MEGF9, DENND6A, ASB7, MTM1, C10orf131, GPR65, NSD1, TREML2, DCAF10, HHEX, ZYG11B, CCM2, OR52K2, ATP11A, CNEP1R1, CARD6, ASB8, HSP90B1, TNFRSF10C, DGKD, INPP5D, FAM217B, HSPA1B, TMEM55A, TLR6, FAM214B, MLXIP, TNFSF10, RAB33B, PADI4, TXNIP, C16orf54, TP53INP1, CNR2, SESN3, C16orf70, ACAP2, NFE2, TLR1 | EDN1, CSF1, FLT1, TRIM25, ANKRD28, LIF, PMAIP1, SERPINB9, HBEGF, ATF3, SGMS2, HCAR2, SDC4, TNFSF14, SERPINE1, SLC3A2, ABL2, SPRY1, TIPARP, PGAP1, INHBA, BHLHE40, PLAU, THAP9-AS1, ZBTB43, SPP1, RCAN1, CD69, GLA, CREM, JUND, ARRDC3, PIGA, CYTH3, KLHL21, JUN, LAMB3, HSPA4L, USP36, FNIP2, HCAR3, ELOVL7, PRNP, NR4A2, GBP2, JOSD1, ZFY, IL12B, TMEM14EP, ZFX, RNF122, HMGCS1, CCL1, RGCC, IL3RA, PTGER2, GABPB1, CAMK1G, BMP6, PPIF, SNAPC1, NAF1, SLC7A5, CCL24, IL3RA, METRNL, LIPN, PNPLA8, SMAD3, DDIT4, MAP2K3, LDLR, HMGCR, SQLE, CXCL16, PPARG, SEC24A, PPTC7, SRGN, STK38L, SLC2A1, TGIF1, MAP4K4, CKS2, RAB7B, USP12, PROK2, DOT1L, B3GNT5, FFAR2, PRKAG2, PHLDA1, ESYT2, ZNFX1, USP12, MDFIC, SLC1A5, RLF, IL1R1, NOP16, OSM, SLC39A8, MSMO1, PLIN2, GPCPD1, TOMM34, CD38, SMOX, BTG3, SLC7A1, PAXBP1, LPL, BNIP3, GPR183, RHOH, CYCS, MAPK6, C15orf39, AHR, C16orf72, C5AR2, TIPARP, ISG20L2, BCL2L11, SBDS, LMNA, DUSP16, FUNDC2, RPF2, S100A10, IMP3, OTUD4, LYRM1, IER5, NCOA7, DOCK10, ATP2C1, EAF1, TWISTNB, ZBTB21, REPIN1, TRIB3, KMO, PTGES, RNF19A, UBAC2, GPR18, FOSB, PHF13, NFIL3, SNX9, ATP1A1, VPS37B, F3, SH3BP5, SLC26A2, VPS13C, GSTO1, AEN, IRAK2, ANKRD42, MCTP1, HMOX2, DDX60L, CLN8, GTPBP4, CDKN1A, GTPBP1, TPRA1, LPXN, DNAJC1, ITPRIP, WWC1, SLA, DUSP16, PFKFB3, LYRM4, SP140, H3F3C, SERPINB2, LAX1, UAP1, PLK3, DDIT3, RALGDS, ITGA5, RAB9A, CCRL2, PRPF39, LONRF3, ATXN1, IARS, BCL2L1, TSPYL2, ZNF331, TMEM52B, ZBTB10, GJB2, TSNAX, ECE1, STOM, DUSP3, DUSP5, KLF10, MTRNR2L5, DUSP6, TCEB1, NOP14, POR, ENPP2, SPAG9, CCNE2, SIAH2, CSRNP1, ATP2B1, SNUPN, NINJ1, ADAM17, TAF4B, NCR3LG1, MMP8, H3F3B, MTHFD1L, GNA15, PFKP, PTGER4, CA2, MED29, PDK1, NFKB2, GPCPD1, CRKL, CHD2, MTRNR2L6, NSMAF, TFRC, KLF2, PIM3, KIR2DL4, HIST2H3A, WDR91, HSPA13, NOCT, YRDC, CLCF1, GABARAPL2 |

**Supplementary Table S3.** Genes in clusters showing z-score changes between BSTOA01 and BSTOA10.

| Cluster 1 | Cluster 2 | Cluster 3 | Cluster 4 | Cluster 6 | Cluster 7 | Cluster 8 |
| --- | --- | --- | --- | --- | --- | --- |
| NAP1L1, CNN2, MYLIP, KIF13A, CASP3, VAV1, KDM3B, TREML2, STAM2, SNX11, PAG1, CASD1, ARID3A, TNFRSF10A, HSP90B1, RASSF2, KDM2A, GYS1, ARMT1, CYBB, ADGRE1, RAB31, TAX1BP1, ZNF644, PPP3CA, F11R, ARNTL, CCDC47, PCNX, PARP1, PLEKHM3, CMTM3, STXBP5, PTPN6, RC3H2, CLOCK, AP2A2, ESCO1, MORC3, C7orf60, ZBTB7B, HEXIM1, LOC388242, BTBD10, FCHSD2, ARAP2, TACC1, EP300, KIAA0232, CLEC4D, SP2, SPOPL, IGF2BP3, TXNDC11, DNAJB1, BTBD9, DYSF, GSK3A, CTSH, CEP170, ZNF609, MFSD6, KIAA0922, SENP2, SMURF2, FGFR1OP2, ENPP4, MMGT1, EPB41, UBE3B, GBA2, TNKS2, PTPN9, EED, TRAPPC3, KPNB1, KDM7A, SNX6, CLIP1, EVI5, TAB3, ABI1, STIM1, PLEKHO1, ARPC5, ZFYVE16, SUSD1, RTN4, INAFM2, ZNF407, PELI1, UBR3, TSPAN2, UBR5, ITCH, FAM103A1, SLC35A5, OSBPL9, CAB39, CAP1, COPZ1, RAB1B, PUDP, DNMT3A, TRIOBP, MFN2, ELK4, NCOA5, DCTD, SLC44A2, DNAJC3, CNTNAP3, ARHGAP18, 45724, POLR3C, ITGAM, DGCR8, STAT3, CD53, ARPC4, ANKRD27, GCNT1, CNOT11, FOXP1, MAP3K7, ENG, U2AF2, OTULIN, RASAL3, WAPL, CLEC4A, ARFGEF2, CEACAM3, HERC3, C7orf43, USO1, KRTAP10-11, METTL23, FAM49B, CCND3, CCND2, UTP11L, LOC101060389, NADK, C6orf191andARHGAP18, AGPAT2, KDM2B, PHYKPL, TMEM222, POM121C, STK24, AP1S2, MPEG1, PARP11, SEC16A, C7orf73, COMMD1, FBXW7, SPCS3, CAPNS2, TIGAR, BAZ2B, BCLAF1, GPR108, TROVE2, SNX14, GLYR1, SZT2, TRMT6, SMCR8, SYNE1, ARL15, CNTNAP3P2, FAF2, MYO9B, SLC23A2, RXRA, ZNF75D, MED13L, PLCL2, NPHP3, MAN1A2, C7orf55-LUC7L2, ADD1, DDB1, CBFA2T2, DYNC1H1, PPP6C, MTHFS, CHM, VPRBP, CARD16, MERTK, VAMP3, TBCEL, CHST12, INO80D, VCPKMT, NBR1, RAB3GAP2, RAC1, PICALM, ERGIC2, GON4L, MAST3, MCEMP1, ALOX5AP, DGKA, ST6GALNAC2, NR6A1, SMG6, STAU2, ATL2, GNG2, LZIC, TANC2, MGAT4B, SLMAP, PRKACA, PCYT1A, AP3B1, TBC1D3, IST1, UBIAD1, DOCK2, C6orf120, VNN1, KLHL2, EFR3A, HGS, SFSWAP, AP3D1, QPCT, SBF2, PDCL, MYADM, ZDHHC4, AOAH, GPR155, S100A11, FMR1, TGS1, SMAP1, TRPS1 | NCOA2, PPP1R12A, FUBP3, NCOA3, KIAA2026, ZMYND11, NIPBL, OSGEP, MTCH1, KAT6B, NRIP1, TBC1D20, PRRC2A, MED20, KIAA1109, RNASEK, BTBD3, BANP, CHP1, DGKD, C20orf24, ATP5J2, INPP5K, PPP6R1 | HSP90B1, LRP10, HSPA1B, HSPH1, GPR65, LTF, RBMS1, PJA2, FAM134B, ENTPD4, MTMR6, SETX, ZYX, PKNOX1, MOB3C, AZIN1, PDLIM5, MLF2, RASA2, VASP, LONRF1, HSPA5, RLIM, CHORDC1 | FAM105A, LEMD3, KAT6A, GALNT7, PRKAA1, TMEM164, SEC24B, MMADHC, ARFGEF1, LRCH1, DOCK11, NT5DC3, MYH9, PSD4, PNPLA6, LCOR, MLKL, PHF8, ITPKB, PHACTR4, FAM199X, HSPE1-MOB4, MTM1, SREK1, UBAP2L, LDAH, SRSF4, RBX1, CDC123, IGF2R, ANKRD17, RFX7, HNRNPUL1, TRIM24, NBEAL2, RBM22, ARMC8, MRPL49, DDX42, STEAP4, PHTF1, LYPLA2, WDFY3, RAD51B, PREX1, LOC646938, RNF5, TRIM13, KMT5B, C16orf62, EPS15, ZCCHC2, CSK, DOCK2, TMEM9B, ARHGAP26, CCDC92, LRRK2, PADI2, FAM193B, CTSS, ELMOD3, FLOT2, CCDC97, MAK16, IFITM1, PIP4K2A, GEN1, LSM14A, FRS2, DHRSX, RPA1, DNAJB12, TMEM59, LSG1, MGAT5, PYGL, IGF1R, SEC14L1, HDHD2, PPHLN1, LILRB2, PDCD10, IDE, KANSL1, ZMYM2, LTA4H, RANBP10, NSDHL, CEP135, PPP6R1, AHCTF1, TTLL3, C10orf88, HSDL2, C1orf112, ANKHD1, ICE1, ARHGAP19-SLIT1, PRKAG1, SARAF, SASS6, TSPAN32, MSH3, CR1, OSBPL2, URI1, KATNBL1, MANEA, TMED10, XPO4, SLC35E1, KANSL3, PADI4, TESK1, ZMPSTE24, CYB5R1, ADGRE3, DDX6, NAA35, GALK2, ATP8B4, KIAA0391, SLC39A4, NR3C1, BTD, CHST15, OCIAD2, RPS26, IFITM3 | THOC1, THAP9-AS1, MSMO1, CHD7, TOMM7, GLS, SMOX, KLF6, ARL6IP1, MT1A, HMGA1, GPR18, EIF4E, COMMD6, GAS7, RPS27A, NDUFA13, GADD45A, HSPA9, TNFSF14, RPL7, BHLHE40, RHOU, GPCPD1, CSTA, SMAD3, ANKRD28, ZBTB43, FUT11, FXYD5, RRNAD1, HMGCR, SRGN, PGAP1, GLA, TMEM63C, BCL2L11, HMOX2, PFKP, ZFAND5, ANP32E, TIPARP, SOD1, OGT, WDR91, CYC1, COX6B1, HIST1H2BD, TSR1, DPY19L1, DDIT3, MTRNR2L5, GSTO1, FBXO34, PSAT1, MSH6, UQCRQ, SLC25A37, PPP2R2D, TARS, NOP14, ANKRD42, ERLEC1, RPF2, TMA7, RNMT, GAB3, VPS13C, GNG5, CABLES1, GNL3, ACBD3, VIM, NKRF, IL3RA, PRKAG2, SLC3A2, TXNDC5, FDPS, LRRC59, BNIP3, CKAP2L, AHR, RALGAPA2, RLF, DNAH1, HIST2H2AC, AEN, HIST1H2AE, NOP16, CERK, FAM133B, CNOT2, PDE12, CLN8, ESYT2, RANBP1, UTP18, LMNA, JAZF1, LRRC8C, CLK3, FUBP1, ERN1, TRIM25, ZNF706, OTUD4, MARVELD3, UHRF1BP1L, FBXW11, CDK9, MT1G, DCUN1D5, RPL14, GPAT3, INHBA, PGK1, NSMAF, SLA, SLC2A1, HIST2H3D, DNAJC1, RPL7A, TRIB3, CFAP20, PSME2, USP6, CSF1, CKS2, MTHFD1L, RPS6KA3, TOMM34, CYTH3, CYCS, GARS, DBI, PLEKHM2, IARS, CSTB, HPRT1, PRPF39, KCTD20, YTHDC1, ATP1A1, UBAC2, ZEB2, PPTC7, S100A10, MTRNR2L3, TNPO2, PROK2, EDEM1, BACH1, TMEM39A, SURF4, TCTN3, TMEM106C, CCDC88A, NPC1, ZNF121, CARS, ELF1, PAXBP1, COX7B, C15orf39, SLC4A7, ACSL5, NEU1, WDR26, H3F3C, FAM19A3, TNNT2, SLC1A5, DAPK3, TCEB1, PFDN5, C1QTNF9B, LRRC42, SLC9A8, TPRA1, PVR, PDCD5, NOC2L, YBX3, GNA15, ZC3H4, PPCDC, PTP4A2, C16orf72, ASNS, CYP51A1, NUP98, NOCT, POMP, HIST2H3A, NR1H2, GTPBP4, LATS2, SQLE, CXorf40B, TMEM14EP, FPGS, ASB3, KLRG2, C19orf25, TPD52, C5AR2, MED29, BMP6, GLRX3, FNIP2, HIST1H2BG, KLF2, USPL1, POGZ, KCNK5, HSPE1, PSMD11, IRAK1, RCAN1, C12orf57, LONRF3, EML2-AS1, MAP4K4, HIST2H2AA3, DDX18, CYTH1, RAB7B, TAF4B, HIGD1A, CDK1, ATXN1, PAIP2, AAED1, EBP, ATF4, PSME1, ZBTB7A, IL1RAP, DUSP6, CRKL, SLC16A1, SLC7A1, ARHGEF7, JUND, SYS1, TTC31, ZFY, ZFX, SEC24A, STXBP1, SLC26A2, WWC1, ZFC3H1, USP11, CSNK1A1, PDK1, ZNFX1, SEC61G, PHLPP1, ERF, H3F3B, FBXO30, PSMA1, ARL8B, TMEM248, IMP3, BRDT, PRAMEF5, PRNP, DKC1, TOP1, FAM188A, HIST2H2AA4, ENSA, WBP4, SEC61B, ECE1, NOLC1, HCCS, GABPB1, CDK16, AP1B1, SFR1, SGMS2, EDN1, RAB9A, SLC43A3, POLR2K, S100P, TGFBRAP1, RQCD1, JTB, HAUS2, HIST1H1E, ATP2C1, RELA, RANBP9, FAM122C, CCL1, ABL2, SNX9, ATP11B, PPRC1, TFG, RGCC, KIAA0907, KPNA4, ANKRD36C, TOR1B, MAFK, RCC2, PRPF38A, MARS, RHEB, CSTF1, LYRM1, SNUPN, STK38L, PLPP6, POLA1, PSMC4, HNRNPM, FAM107B, FNBP1, CLIC1, HSPA14, HSPA4L, ESYT1, DERL1, GNA13, HCST, TFRC | KLHL21, ITGA5, RNF122, MTRNR2L6, PGS1, SLC38A2, RPS20, RPL39, GPCPD1, RPL7A, C16orf91, RPS14, EEF1D, MTRNR2L2, TSNAX, MTRNR2L9, MRPL12, HIST1H2AI, RPLP0, ZNF33A, H2AFX, TOMM22, SNORD102, TIMM10B, PHF6, H2AFZ, HIST1H1B, BRIX1, RPL35, RPS21, RPS12, IRF2BP2, ZNF12, RPS29, EIF3D, RPL23 | ANXA1, PHF13, AKIRIN2, HIST1H3B, STK17B, NIFK, HIST1H2AB, ND6 |

**Supplementary Table S4.** Enriched transcription factor genes in each cluster.

| BSTOA01_up | BSTOA01_down | BSTOA10_down | BSTOA10_up | BSTOA01_up & BSTOA01_down | BSTOA01_up & BSTOA10_down | BSTOA01_up & BSTOA10_up | BSTOA01_down & BSTOA10_down | BSTOA01_down BSTOA10_up | BSTOA10_down BSTOA10_up | BSTOA01_up & BSTOA01_down & BSTOA10_down | BSTOA01_up & BSTOA01_down & BSTOA10_up | BSTOA01_up & BSTOA10_down & BSTOA10_up | BSTOA01_down & BSTOA10_down & BSTOA10_up | BSTOA01_up & BSTOA01_down & BSTOA10_down & BSTOA10_up |
| --- | --- | --- | --- | --- | --- | --- | --- | --- | --- | --- | --- | --- | --- | --- |
| CHD8, XBP1, ZNF33A, FOXO3 | CDK7, CDK8, NCOA1, IRF1, PRKDC, GTF2B, PIAS1, KAT2B, ASH2L, HDAC3, RBAK, ZNF257, RNF2, CDC5L, ZNF350, JMJD6 | HDAC1, KDM1A, LMO2, TCF3, MYBL2, ZNF224, RB1, FOXK2, ZHX1, STAT5B, WDR5, SREBF2, NFE2, BRD7, ZEB1, DPF2, ARID3A, MGA, MCRS1, USF2, NONO, IKZF1, ZMYND11, RCOR1, UBTF, ATF1, SMAD5, NFYA, ZBTB7B, NCOA3 | ATF4, MED12, NFATC2, KDM4A, TET2, BATF3, KLF10, TLE3, CEBPZ, CEBPG, RBM39, BHLHE40, ZZZ3, ICE1, TOP1, ARRB1, ZNF207, ZNF318, STAT2, KLF2, TRIM25, RLF, ZBTB43, ZNFX1, TCEB1, TRIB3 | STAG1, FOXM1 | MYB, BCL6, NFYB, SMAD4, BCL11A, ATF7 | ELF4 | VDR, CHD4, GTF2F1, JMJD1C | ARNTL, ZBTB11, JARID2, PPARG, SATB1 | SMARCA2, BRCA1, FOSL2, SP2, PHF8, OGT, NFRKB, NFKB1, ZFX, ZNF41, ZFY, SUZ12, BDP1, SS18, PRDM1 | SMC3 | CEBPA, E2F4, KDM5A, SMARCC1, MAZ | EP300, HCFC1, JUNB, NR3C1, NFIC, ATF2, BACH1 | NR2C2, RARA, ZNF143 | CTCF, RAD21, POLR2A, SPI1, MYC, RELA, SUPT5H, BRD4, RUNX1, CEBPB, HSF1, NELFA, CREB1, SIN3A, NRF1, KMT2A, TAF1, MED1, TBP, MAX, CHD1, MAFK, JUND, MXI1, EZH2, REST, SMC1A, YY1, SMARCA4, AFF4, SRF, STAT1, JUN, NIPBL, ELL2, EGR1, AHR, ZBTB33, OGG1, SMAD2, GABPA, FOS, HMGB2, BRD2, RFX5, NFE2L2, SMAD3, BRD3, CHD2, STAT3, ELF1, SETDB1, TCF12, KLF5, HDAC2, HIF1A, RXRA, SP1, ARNT, MEF2A, TAF7, STAT5A, ELK1, PML, ATF3, HMGB1, CBX3, SAP30, TBL1XR1, ZNF75A, WDHD1, MEN1, RBBP5, CHD7, FOSL1, DDX21, CTNNB1, HDAC6, PBX3, CDK9, ZMYND8, BCL3 |

**Supplementary Table S5.** List of excluded GO terms.

| GOID | GOTerm |
| --- | --- |
| GO:0000120 | RNA polymerase I transcription regulator complex |
| GO:0000122 | negative regulation of transcription by RNA polymerase II |
| GO:0000126 | transcription factor TFIIIB complex |
| GO:0000127 | transcription factor TFIIIC complex |
| GO:0000244 | spliceosomal tri-snRNP complex assembly |
| GO:0000245 | spliceosomal complex assembly |
| GO:0000343 | plastid-encoded plastid RNA polymerase complex A |
| GO:0000344 | plastid-encoded plastid RNA polymerase complex B |
| GO:0000345 | cytosolic DNA-directed RNA polymerase complex |
| GO:0000346 | transcription export complex |
| GO:0000349 | generation of catalytic spliceosome for first transesterification step |
| GO:0000350 | generation of catalytic spliceosome for second transesterification step |
| GO:0000352 | trans assembly of SL-containing precatalytic spliceosome |
| GO:0000354 | cis assembly of pre-catalytic spliceosome |
| GO:0000365 | mRNA trans splicing, via spliceosome |
| GO:0000366 | intergenic mRNA trans splicing |
| GO:0000372 | Group I intron splicing |
| GO:0000373 | Group II intron splicing |
| GO:0000374 | Group III intron splicing |
| GO:0000375 | RNA splicing, via transesterification reactions |
| GO:0000376 | RNA splicing, via transesterification reactions with guanosine as nucleophile |
| GO:0000377 | RNA splicing, via transesterification reactions with bulged adenosine as nucleophile |
| GO:0000379 | tRNA-type intron splice site recognition and cleavage |
| GO:0000380 | alternative mRNA splicing, via spliceosome |
| GO:0000381 | regulation of alternative mRNA splicing, via spliceosome |
| GO:0000384 | first spliceosomal transesterification activity |
| GO:0000386 | second spliceosomal transesterification activity |
| GO:0000387 | spliceosomal snRNP assembly |
| GO:0000388 | spliceosome conformational change to release U4 (or U4atac) and U1 (or U11) |
| GO:0000389 | mRNA 3'-splice site recognition |
| GO:0000390 | spliceosomal complex disassembly |
| GO:0000393 | spliceosomal conformational changes to generate catalytic conformation |
| GO:0000394 | RNA splicing, via endonucleolytic cleavage and ligation |
| GO:0000395 | mRNA 5'-splice site recognition |
| GO:0000398 | mRNA splicing, via spliceosome |
| GO:0000409 | regulation of transcription by galactose |
| GO:0000410 | carbon catabolite repression of transcription by galactose |
| GO:0000411 | positive regulation of transcription by galactose |
| GO:0000418 | RNA polymerase IV complex |
| GO:0000419 | RNA polymerase V complex |
| GO:0000427 | plastid-encoded plastid RNA polymerase complex |
| GO:0000428 | DNA-directed RNA polymerase complex |
| GO:0000429 | carbon catabolite regulation of transcription from RNA polymerase II promoter |
| GO:0000430 | regulation of transcription from RNA polymerase II promoter by glucose |
| GO:0000431 | regulation of transcription from RNA polymerase II promoter by galactose |
| GO:0000432 | positive regulation of transcription from RNA polymerase II promoter by glucose |
| GO:0000433 | carbon catabolite repression of transcription from RNA polymerase II promoter by glucose |
| GO:0000434 | carbon catabolite repression of transcription from RNA polymerase II promoter by galactose |
| GO:0000435 | positive regulation of transcription from RNA polymerase II promoter by galactose |
| GO:0000436 | carbon catabolite activation of transcription from RNA polymerase II promoter |
| GO:0000437 | carbon catabolite repression of transcription from RNA polymerase II promoter |
| GO:0000439 | transcription factor TFIIH core complex |
| GO:0000445 | THO complex part of transcription export complex |
| GO:0000500 | RNA polymerase I upstream activating factor complex |
| GO:0000512 | lncRNA-mediated post-transcriptional gene silencing |
| GO:0000716 | transcription-coupled nucleotide-excision repair, DNA damage recognition |
| GO:0000900 | mRNA regulatory element binding translation repressor activity |
| GO:0000901 | translation repressor activity, non-nucleic acid binding |
| GO:0000963 | mitochondrial RNA processing |
| GO:0000972 | transcription-dependent tethering of RNA polymerase II gene DNA at nuclear periphery |
| GO:0000973 | post-transcriptional tethering of RNA polymerase II gene DNA at nuclear periphery |
| GO:0000976 | transcription cis-regulatory region binding |
| GO:0000977 | RNA polymerase II transcription regulatory region sequence-specific DNA binding |
| GO:0000978 | RNA polymerase II cis-regulatory region sequence-specific DNA binding |
| GO:0000979 | RNA polymerase II core promoter sequence-specific DNA binding |
| GO:0000981 | DNA-binding transcription factor activity, RNA polymerase II-specific |
| GO:0000992 | RNA polymerase III cis-regulatory region sequence-specific DNA binding |
| GO:0000993 | RNA polymerase II complex binding |
| GO:0000994 | RNA polymerase III core binding |
| GO:0000995 | RNA polymerase III general transcription initiation factor activity |
| GO:0001000 | bacterial-type RNA polymerase core enzyme binding |
| GO:0001001 | mitochondrial single-subunit type RNA polymerase binding |
| GO:0001002 | RNA polymerase III type 1 promoter sequence-specific DNA binding |
| GO:0001003 | RNA polymerase III type 2 promoter sequence-specific DNA binding |
| GO:0001006 | RNA polymerase III type 3 promoter sequence-specific DNA binding |
| GO:0001010 | RNA polymerase II sequence-specific DNA-binding transcription factor recruiting activity |
| GO:0001014 | snoRNA transcription by RNA polymerase III |
| GO:0001015 | snoRNA transcription by RNA polymerase II |
| GO:0001016 | RNA polymerase III transcription regulatory region sequence-specific DNA binding |
| GO:0001019 | plastid promoter transcription regulatory region sequence-specific DNA binding |
| GO:0001025 | RNA polymerase III general transcription initiation factor binding |
| GO:0001039 | RNA polymerase III hybrid type promoter sequence-specific DNA binding |
| GO:0001042 | RNA polymerase I core binding |
| GO:0001048 | RNA polymerase IV core binding |
| GO:0001049 | RNA polymerase V core binding |
| GO:0001050 | single-subunit type RNA polymerase binding |
| GO:0001051 | plastid single-subunit type RNA polymerase binding |
| GO:0001052 | plastid PEP RNA polymerase core enzyme binding |
| GO:0001054 | RNA polymerase I activity |
| GO:0001055 | RNA polymerase II activity |
| GO:0001056 | RNA polymerase III activity |
| GO:0001057 | RNA polymerase IV activity |
| GO:0001058 | RNA polymerase V activity |
| GO:0001059 | transcription by RNA polymerase IV |
| GO:0001060 | transcription by RNA polymerase V |
| GO:0001064 | single subunit type RNA polymerase activity |
| GO:0001065 | mitochondrial single subunit type RNA polymerase activity |
| GO:0001066 | plastid single subunit type RNA polymerase activity |
| GO:0001067 | transcription regulatory region nucleic acid binding |
| GO:0001068 | transcription regulatory region RNA binding |
| GO:0001070 | RNA-binding transcription regulator activity |
| GO:0001072 | transcription antitermination factor activity, RNA binding |
| GO:0001073 | transcription antitermination factor activity, DNA binding |
| GO:0001079 | nitrogen catabolite regulation of transcription from RNA polymerase II promoter |
| GO:0001080 | nitrogen catabolite activation of transcription from RNA polymerase II promoter |
| GO:0001081 | nitrogen catabolite repression of transcription from RNA polymerase II promoter |
| GO:0001091 | RNA polymerase II general transcription initiation factor binding |
| GO:0001092 | TFIIA-class transcription factor complex binding |
| GO:0001093 | TFIIB-class transcription factor binding |
| GO:0001094 | TFIID-class transcription factor complex binding |
| GO:0001095 | TFIIE-class transcription factor complex binding |
| GO:0001096 | TFIIF-class transcription factor complex binding |
| GO:0001097 | TFIIH-class transcription factor complex binding |
| GO:0001098 | basal transcription machinery binding |
| GO:0001099 | basal RNA polymerase II transcription machinery binding |
| GO:0001108 | bacterial-type RNA polymerase holo enzyme binding |
| GO:0001109 | promoter clearance during DNA-templated transcription |
| GO:0001110 | RNA polymerase III promoter clearance |
| GO:0001111 | RNA polymerase II promoter clearance |
| GO:0001112 | DNA-templated transcription open complex formation |
| GO:0001113 | transcription open complex formation at RNA polymerase II promoter |
| GO:0001118 | transcription ternary complex disassembly |
| GO:0001139 | RNA polymerase II complex recruiting activity |
| GO:0001147 | transcription termination site sequence-specific DNA binding |
| GO:0001154 | TFIIIB-class transcription factor complex binding |
| GO:0001155 | TFIIIA-class transcription factor binding |
| GO:0001156 | TFIIIC-class transcription factor complex binding |
| GO:0001161 | intronic transcription regulatory region sequence-specific DNA binding |
| GO:0001162 | RNA polymerase II intronic transcription regulatory region sequence-specific DNA binding |
| GO:0001163 | RNA polymerase I transcription regulatory region sequence-specific DNA binding |
| GO:0001164 | RNA polymerase I core promoter sequence-specific DNA binding |
| GO:0001165 | RNA polymerase I cis-regulatory region sequence-specific DNA binding |
| GO:0001171 | reverse transcription |
| GO:0001172 | RNA-templated transcription |
| GO:0001173 | DNA-templated transcriptional start site selection |
| GO:0001174 | transcriptional start site selection at RNA polymerase II promoter |
| GO:0001175 | transcriptional start site selection at RNA polymerase III promoter |
| GO:0001177 | regulation of transcription open complex formation at RNA polymerase II promoter |
| GO:0001178 | regulation of transcriptional start site selection at RNA polymerase II promoter |
| GO:0001179 | RNA polymerase I general transcription initiation factor binding |
| GO:0001181 | RNA polymerase I general transcription initiation factor activity |
| GO:0001182 | RNA polymerase I promoter clearance |
| GO:0001188 | RNA polymerase I preinitiation complex assembly |
| GO:0001192 | maintenance of transcriptional fidelity during transcription elongation |
| GO:0001193 | maintenance of transcriptional fidelity during transcription elongation by RNA polymerase II |
| GO:0001195 | maintenance of transcriptional fidelity during transcription elongation by RNA polymerase III |
| GO:0001216 | DNA-binding transcription activator activity |
| GO:0001217 | DNA-binding transcription repressor activity |
| GO:0001221 | transcription coregulator binding |
| GO:0001222 | transcription corepressor binding |
| GO:0001223 | transcription coactivator binding |
| GO:0001227 | DNA-binding transcription repressor activity, RNA polymerase II-specific |
| GO:0001228 | DNA-binding transcription activator activity, RNA polymerase II-specific |
| GO:0001677 | formation of translation initiation ternary complex |
| GO:0001731 | formation of translation preinitiation complex |
| GO:0001732 | formation of cytoplasmic translation initiation complex |
| GO:0002107 | generation of mature 3'-end of 5S rRNA generated by RNA polymerase III |
| GO:0002110 | cotranscriptional mitochondrial rRNA nucleotide insertion |
| GO:0002181 | cytoplasmic translation |
| GO:0002182 | cytoplasmic translational elongation |
| GO:0002183 | cytoplasmic translational initiation |
| GO:0002184 | cytoplasmic translational termination |
| GO:0002188 | translation reinitiation |
| GO:0002190 | cap-independent translational initiation |
| GO:0002191 | cap-dependent translational initiation |
| GO:0002192 | IRES-dependent translational initiation of linear mRNA |
| GO:0002330 | pre-B cell receptor expression |
| GO:0002563 | somatic diversification of immune receptors via alternate splicing |
| GO:0002564 | alternate splicing of immunoglobulin genes |
| GO:0003700 | DNA-binding transcription factor activity |
| GO:0003711 | transcription elongation factor activity |
| GO:0003712 | transcription coregulator activity |
| GO:0003713 | transcription coactivator activity |
| GO:0003714 | transcription corepressor activity |
| GO:0003743 | translation initiation factor activity |
| GO:0003746 | translation elongation factor activity |
| GO:0003747 | translation release factor activity |
| GO:0003899 | DNA-directed 5'-3' RNA polymerase activity |
| GO:0003968 | RNA-dependent RNA polymerase activity |
| GO:0004694 | eukaryotic translation initiation factor 2alpha kinase activity |
| GO:0005665 | RNA polymerase II, core complex |
| GO:0005666 | RNA polymerase III complex |
| GO:0005667 | transcription regulator complex |
| GO:0005668 | RNA polymerase transcription factor SL1 complex |
| GO:0005669 | transcription factor TFIID complex |
| GO:0005672 | transcription factor TFIIA complex |
| GO:0005673 | transcription factor TFIIE complex |
| GO:0005674 | transcription factor TFIIF complex |
| GO:0005675 | transcription factor TFIIH holo complex |
| GO:0005681 | spliceosomal complex |
| GO:0005684 | U2-type spliceosomal complex |
| GO:0005689 | U12-type spliceosomal complex |
| GO:0005736 | RNA polymerase I complex |
| GO:0005850 | eukaryotic translation initiation factor 2 complex |
| GO:0005851 | eukaryotic translation initiation factor 2B complex |
| GO:0005852 | eukaryotic translation initiation factor 3 complex |
| GO:0005853 | eukaryotic translation elongation factor 1 complex |
| GO:0006283 | transcription-coupled nucleotide-excision repair |
| GO:0006351 | DNA-templated transcription |
| GO:0006352 | DNA-templated transcription initiation |
| GO:0006353 | DNA-templated transcription termination |
| GO:0006354 | DNA-templated transcription elongation |
| GO:0006355 | regulation of DNA-templated transcription |
| GO:0006356 | regulation of transcription by RNA polymerase I |
| GO:0006357 | regulation of transcription by RNA polymerase II |
| GO:0006359 | regulation of transcription by RNA polymerase III |
| GO:0006360 | transcription by RNA polymerase I |
| GO:0006361 | transcription initiation at RNA polymerase I promoter |
| GO:0006362 | transcription elongation by RNA polymerase I |
| GO:0006363 | termination of RNA polymerase I transcription |
| GO:0006364 | rRNA processing |
| GO:0006366 | transcription by RNA polymerase II |
| GO:0006367 | transcription initiation at RNA polymerase II promoter |
| GO:0006368 | transcription elongation by RNA polymerase II |
| GO:0006369 | termination of RNA polymerase II transcription |
| GO:0006376 | mRNA splice site recognition |
| GO:0006383 | transcription by RNA polymerase III |
| GO:0006384 | transcription initiation at RNA polymerase III promoter |
| GO:0006385 | transcription elongation by RNA polymerase III |
| GO:0006386 | termination of RNA polymerase III transcription |
| GO:0006388 | tRNA splicing, via endonucleolytic cleavage and ligation |
| GO:0006390 | mitochondrial transcription |
| GO:0006391 | transcription initiation at mitochondrial promoter |
| GO:0006392 | transcription elongation by mitochondrial RNA polymerase |
| GO:0006393 | termination of mitochondrial transcription |
| GO:0006396 | RNA processing |
| GO:0006397 | mRNA processing |
| GO:0006412 | translation |
| GO:0006413 | translational initiation |
| GO:0006414 | translational elongation |
| GO:0006415 | translational termination |
| GO:0006417 | regulation of translation |
| GO:0006418 | tRNA aminoacylation for protein translation |
| GO:0006446 | regulation of translational initiation |
| GO:0006447 | regulation of translational initiation by iron |
| GO:0006448 | regulation of translational elongation |
| GO:0006449 | regulation of translational termination |
| GO:0006450 | regulation of translational fidelity |
| GO:0006451 | translational readthrough |
| GO:0006452 | translational frameshifting |
| GO:0006613 | cotranslational protein targeting to membrane |
| GO:0006614 | SRP-dependent cotranslational protein targeting to membrane |
| GO:0006615 | SRP-dependent cotranslational protein targeting to membrane, docking |
| GO:0006616 | SRP-dependent cotranslational protein targeting to membrane, translocation |
| GO:0006617 | SRP-dependent cotranslational protein targeting to membrane, signal sequence recognition |
| GO:0006618 | SRP-dependent cotranslational protein targeting to membrane, signal sequence processing |
| GO:0006620 | post-translational protein targeting to endoplasmic reticulum membrane |
| GO:0006978 | DNA damage response, signal transduction by p53 class mediator resulting in transcription of p21 class mediator |
| GO:0007221 | positive regulation of transcription of Notch receptor target |
| GO:0007319 | negative regulation of oskar mRNA translation |
| GO:0007532 | regulation of mating-type specific transcription, DNA-templated |
| GO:0008023 | transcription elongation factor complex |
| GO:0008024 | cyclin/CDK positive transcription elongation factor complex |
| GO:0008033 | tRNA processing |
| GO:0008079 | translation termination factor activity |
| GO:0008134 | transcription factor binding |
| GO:0008135 | translation factor activity, RNA binding |
| GO:0008353 | RNA polymerase II CTD heptapeptide repeat kinase activity |
| GO:0008380 | RNA splicing |
| GO:0008420 | RNA polymerase II CTD heptapeptide repeat phosphatase activity |
| GO:0008494 | translation activator activity |
| GO:0009299 | mRNA transcription |
| GO:0009300 | antisense RNA transcription |
| GO:0009301 | snRNA transcription |
| GO:0009302 | sno(s)RNA transcription |
| GO:0009303 | rRNA transcription |
| GO:0009304 | tRNA transcription |
| GO:0009386 | translational attenuation |
| GO:0010239 | chloroplast mRNA processing |
| GO:0010267 | ta-siRNA processing |
| GO:0010467 | gene expression |
| GO:0010468 | regulation of gene expression |
| GO:0010495 | siRNA-mediated long-distance post-transcriptional gene silencing |
| GO:0010599 | lsiRNA processing |
| GO:0010603 | regulation of cytoplasmic mRNA processing body assembly |
| GO:0010606 | positive regulation of cytoplasmic mRNA processing body assembly |
| GO:0010607 | negative regulation of cytoplasmic mRNA processing body assembly |
| GO:0010608 | post-transcriptional regulation of gene expression |
| GO:0010609 | mRNA localization resulting in post-transcriptional regulation of gene expression |
| GO:0010620 | negative regulation of transcription by transcription factor catabolism |
| GO:0010621 | negative regulation of transcription by transcription factor localization |
| GO:0010628 | positive regulation of gene expression |
| GO:0010629 | negative regulation of gene expression |
| GO:0010630 | regulation of transcription, start site selection |
| GO:0010688 | negative regulation of ribosomal protein gene transcription by RNA polymerase II |
| GO:0010689 | negative regulation of ribosomal protein gene transcription from RNA polymerase II promoter in response to chemical stimulus |
| GO:0010690 | negative regulation of ribosomal protein gene transcription from RNA polymerase II promoter in response to stress |
| GO:0010691 | negative regulation of ribosomal protein gene transcription from RNA polymerase II promoter in response to nutrient levels |
| GO:0010892 | positive regulation of mitochondrial translation in response to stress |
| GO:0010944 | negative regulation of transcription by competitive promoter binding |
| GO:0010998 | regulation of translational initiation by eIF2 alpha phosphorylation |
| GO:0016149 | translation release factor activity, codon specific |
| GO:0016150 | translation release factor activity, codon nonspecific |
| GO:0016180 | snRNA processing |
| GO:0016251 | RNA polymerase II general transcription initiation factor activity |
| GO:0016281 | eukaryotic translation initiation factor 4F complex |
| GO:0016441 | post-transcriptional gene silencing |
| GO:0016456 | X chromosome located dosage compensation complex, transcription activating |
| GO:0016479 | negative regulation of transcription by RNA polymerase I |
| GO:0016480 | negative regulation of transcription by RNA polymerase III |
| GO:0016539 | intein-mediated protein splicing |
| GO:0016591 | RNA polymerase II, holoenzyme |
| GO:0017053 | transcription repressor complex |
| GO:0017055 | negative regulation of RNA polymerase II transcription preinitiation complex assembly |
| GO:0017148 | negative regulation of translation |
| GO:0018444 | translation release factor complex |
| GO:0019036 | viral transcriptional complex |
| GO:0019057 | symbiont-mediated perturbation of host translation |
| GO:0019080 | viral gene expression |
| GO:0019081 | viral translation |
| GO:0019083 | viral transcription |
| GO:0019084 | middle viral transcription |
| GO:0019085 | early viral transcription |
| GO:0019086 | late viral transcription |
| GO:0019801 | cyclization of asparagine involved in intein-mediated protein splicing |
| GO:0019802 | cyclization of glutamine involved in intein-mediated protein splicing |
| GO:0023019 | signal transduction involved in regulation of gene expression |
| GO:0030371 | translation repressor activity |
| GO:0030422 | siRNA processing |
| GO:0030627 | pre-mRNA 5'-splice site binding |
| GO:0030628 | pre-mRNA 3'-splice site binding |
| GO:0030846 | termination of RNA polymerase II transcription, poly(A)-coupled |
| GO:0030847 | termination of RNA polymerase II transcription, exosome-dependent |
| GO:0030880 | RNA polymerase complex |
| GO:0030907 | MBF transcription complex |
| GO:0030908 | protein splicing |
| GO:0030909 | non-intein-mediated protein splicing |
| GO:0031053 | primary miRNA processing |
| GO:0031054 | pre-miRNA processing |
| GO:0031070 | intronic snoRNA processing |
| GO:0031203 | post-translational protein targeting to membrane, docking |
| GO:0031204 | post-translational protein targeting to membrane, translocation |
| GO:0031369 | translation initiation factor binding |
| GO:0031379 | RNA-directed RNA polymerase complex |
| GO:0031380 | nuclear RNA-directed RNA polymerase complex |
| GO:0031381 | viral RNA-directed RNA polymerase complex |
| GO:0031425 | chloroplast RNA processing |
| GO:0031426 | polycistronic mRNA processing |
| GO:0031554 | regulation of termination of DNA-templated transcription |
| GO:0031555 | transcriptional attenuation |
| GO:0031556 | transcriptional attenuation by ribosome |
| GO:0031564 | transcription antitermination |
| GO:0032055 | negative regulation of translation in response to stress |
| GO:0032056 | positive regulation of translation in response to stress |
| GO:0032057 | negative regulation of translational initiation in response to stress |
| GO:0032058 | positive regulation of translational initiation in response to stress |
| GO:0032061 | negative regulation of translation in response to osmotic stress |
| GO:0032062 | positive regulation of translation in response to osmotic stress |
| GO:0032063 | negative regulation of translational initiation in response to osmotic stress |
| GO:0032064 | positive regulation of translational initiation in response to osmotic stress |
| GO:0032088 | negative regulation of NF-kappaB transcription factor activity |
| GO:0032543 | mitochondrial translation |
| GO:0032544 | plastid translation |
| GO:0032784 | regulation of DNA-templated transcription elongation |
| GO:0032785 | negative regulation of DNA-templated transcription, elongation |
| GO:0032786 | positive regulation of DNA-templated transcription, elongation |
| GO:0032792 | negative regulation of CREB transcription factor activity |
| GO:0032793 | positive regulation of CREB transcription factor activity |
| GO:0032897 | negative regulation of viral transcription |
| GO:0032922 | circadian regulation of gene expression |
| GO:0032938 | negative regulation of translation in response to oxidative stress |
| GO:0032939 | positive regulation of translation in response to oxidative stress |
| GO:0032968 | positive regulation of transcription elongation by RNA polymerase II |
| GO:0033119 | negative regulation of RNA splicing |
| GO:0033120 | positive regulation of RNA splicing |
| GO:0033276 | transcription factor TFTC complex |
| GO:0033309 | SBF transcription complex |
| GO:0033562 | co-transcriptional gene silencing by RNA interference machinery |
| GO:0034062 | 5'-3' RNA polymerase activity |
| GO:0034243 | regulation of transcription elongation by RNA polymerase II |
| GO:0034244 | negative regulation of transcription elongation by RNA polymerase II |
| GO:0034245 | mitochondrial DNA-directed RNA polymerase complex |
| GO:0034246 | mitochondrial transcription factor activity |
| GO:0034247 | snoRNA splicing |
| GO:0034402 | recruitment of 3'-end processing factors to RNA polymerase II holoenzyme complex |
| GO:0034403 | alignment of 3' and 5' splice sites of mRNA |
| GO:0034420 | co-translational protein acetylation |
| GO:0034421 | post-translational protein acetylation |
| GO:0034470 | ncRNA processing |
| GO:0034587 | piRNA processing |
| GO:0034732 | transcription factor TFIIIB-alpha complex |
| GO:0034733 | transcription factor TFIIIB-beta complex |
| GO:0034734 | transcription factor TFIIIC1 complex |
| GO:0034735 | transcription factor TFIIIC2 complex |
| GO:0034963 | box C/D RNA processing |
| GO:0034964 | box H/ACA RNA processing |
| GO:0034965 | intronic box C/D RNA processing |
| GO:0034966 | intronic box H/ACA snoRNA processing |
| GO:0035194 | regulatory ncRNA-mediated post-transcriptional gene silencing |
| GO:0035195 | miRNA-mediated post-transcriptional gene silencing |
| GO:0035196 | miRNA processing |
| GO:0035278 | miRNA-mediated gene silencing by inhibition of translation |
| GO:0035976 | transcription factor AP-1 complex |
| GO:0036031 | recruitment of mRNA capping enzyme to RNA polymerase II holoenzyme complex |
| GO:0036490 | regulation of translation in response to endoplasmic reticulum stress |
| GO:0036491 | regulation of translation initiation in response to endoplasmic reticulum stress |
| GO:0036493 | positive regulation of translation in response to endoplasmic reticulum stress |
| GO:0036494 | positive regulation of translation initiation in response to endoplasmic reticulum stress |
| GO:0036495 | negative regulation of translation initiation in response to endoplasmic reticulum stress |
| GO:0036496 | regulation of translational initiation by eIF2 alpha dephosphorylation |
| GO:0039523 | symbiont-mediated suppression of host mRNA transcription via inhibition of RNA polymerase II activity |
| GO:0039524 | symbiont-mediated suppression of host mRNA processing |
| GO:0039602 | symbiont-mediated suppression of host transcription initiation from RNA polymerase II promoter |
| GO:0039604 | symbiont-mediated suppression of host translation |
| GO:0039606 | symbiont-mediated suppression of host translation initiation |
| GO:0039653 | symbiont-mediated suppression of host transcription |
| GO:0039656 | symbiont-mediated perturbation of host gene expression |
| GO:0039657 | symbiont-mediated suppression of host gene expression |
| GO:0039688 | viral double stranded DNA replication via reverse transcription |
| GO:0039695 | DNA-templated viral transcription |
| GO:0039696 | RNA-templated viral transcription |
| GO:0039697 | negative stranded viral RNA transcription |
| GO:0039704 | viral translational shunt |
| GO:0039705 | viral translational readthrough |
| GO:0040029 | epigenetic regulation of gene expression |
| GO:0040033 | sRNA-mediated post-transcriptional gene silencing |
| GO:0042772 | DNA damage response, signal transduction resulting in transcription |
| GO:0042781 | 3'-tRNA processing endoribonuclease activity |
| GO:0042789 | mRNA transcription by RNA polymerase II |
| GO:0042790 | nucleolar large rRNA transcription by RNA polymerase I |
| GO:0042791 | 5S class rRNA transcription by RNA polymerase III |
| GO:0042793 | plastid transcription |
| GO:0042794 | plastid rRNA transcription |
| GO:0042795 | snRNA transcription by RNA polymerase II |
| GO:0042796 | snRNA transcription by RNA polymerase III |
| GO:0042797 | tRNA transcription by RNA polymerase III |
| GO:0042994 | cytoplasmic sequestering of transcription factor |
| GO:0043045 | post-fertilization epigenetic regulation of gene expression |
| GO:0043143 | regulation of translation by machinery localization |
| GO:0043144 | sno(s)RNA processing |
| GO:0043175 | RNA polymerase core enzyme binding |
| GO:0043425 | bHLH transcription factor binding |
| GO:0043433 | negative regulation of DNA-binding transcription factor activity |
| GO:0043484 | regulation of RNA splicing |
| GO:0043555 | regulation of translation in response to stress |
| GO:0043556 | regulation of translation in response to oxidative stress |
| GO:0043557 | regulation of translation in response to osmotic stress |
| GO:0043558 | regulation of translational initiation in response to stress |
| GO:0043561 | regulation of translational initiation in response to osmotic stress |
| GO:0043686 | co-translational protein modification |
| GO:0043687 | post-translational protein modification |
| GO:0043921 | modulation by host of viral transcription |
| GO:0043922 | negative regulation by host of viral transcription |
| GO:0043923 | positive regulation by host of viral transcription |
| GO:0044027 | negative regulation of gene expression via CpG island methylation |
| GO:0044029 | positive regulation of gene expression via CpG island demethylation |
| GO:0044207 | translation initiation ternary complex |
| GO:0044377 | RNA polymerase II cis-regulatory region sequence-specific DNA binding, bending |
| GO:0044530 | supraspliceosomal complex |
| GO:0045013 | carbon catabolite repression of transcription |
| GO:0045014 | carbon catabolite repression of transcription by glucose |
| GO:0045182 | translation regulator activity |
| GO:0045183 | translation factor activity, non-nucleic acid binding |
| GO:0045291 | mRNA trans splicing, SL addition |
| GO:0045292 | mRNA cis splicing, via spliceosome |
| GO:0045727 | positive regulation of translation |
| GO:0045814 | negative regulation of gene expression, epigenetic |
| GO:0045815 | transcription initiation-coupled chromatin remodeling |
| GO:0045892 | negative regulation of DNA-templated transcription |
| GO:0045893 | positive regulation of DNA-templated transcription |
| GO:0045894 | negative regulation of mating-type specific transcription, DNA-templated |
| GO:0045895 | positive regulation of mating-type specific transcription, DNA-templated |
| GO:0045898 | regulation of RNA polymerase II transcription preinitiation complex assembly |
| GO:0045899 | positive regulation of RNA polymerase II transcription preinitiation complex assembly |
| GO:0045900 | negative regulation of translational elongation |
| GO:0045901 | positive regulation of translational elongation |
| GO:0045902 | negative regulation of translational fidelity |
| GO:0045903 | positive regulation of translational fidelity |
| GO:0045904 | negative regulation of translational termination |
| GO:0045905 | positive regulation of translational termination |
| GO:0045943 | positive regulation of transcription by RNA polymerase I |
| GO:0045944 | positive regulation of transcription by RNA polymerase II |
| GO:0045945 | positive regulation of transcription by RNA polymerase III |
| GO:0045947 | negative regulation of translational initiation |
| GO:0045948 | positive regulation of translational initiation |
| GO:0045974 | regulation of translation, ncRNA-mediated |
| GO:0045975 | positive regulation of translation, ncRNA-mediated |
| GO:0045990 | carbon catabolite regulation of transcription |
| GO:0045991 | carbon catabolite activation of transcription |
| GO:0045993 | negative regulation of translational initiation by iron |
| GO:0045994 | positive regulation of translational initiation by iron |
| GO:0046011 | regulation of oskar mRNA translation |
| GO:0046012 | positive regulation of oskar mRNA translation |
| GO:0046015 | regulation of transcription by glucose |
| GO:0046016 | positive regulation of transcription by glucose |
| GO:0046773 | symbiont-mediated suppression of host translation termination |
| GO:0046780 | symbiont-mediated suppression of host mRNA splicing |
| GO:0046782 | regulation of viral transcription |
| GO:0048024 | regulation of mRNA splicing, via spliceosome |
| GO:0048025 | negative regulation of mRNA splicing, via spliceosome |
| GO:0048026 | positive regulation of mRNA splicing, via spliceosome |
| GO:0050434 | positive regulation of viral transcription |
| GO:0050684 | regulation of mRNA processing |
| GO:0050685 | positive regulation of mRNA processing |
| GO:0050686 | negative regulation of mRNA processing |
| GO:0051083 | 'de novo' cotranslational protein folding |
| GO:0051084 | 'de novo' post-translational protein folding |
| GO:0051090 | regulation of DNA-binding transcription factor activity |
| GO:0051091 | positive regulation of DNA-binding transcription factor activity |
| GO:0051092 | positive regulation of NF-kappaB transcription factor activity |
| GO:0051123 | RNA polymerase II preinitiation complex assembly |
| GO:0052026 | symbiont-mediated perturbation of host transcription |
| GO:0052472 | modulation by host of symbiont transcription |
| GO:0055029 | nuclear DNA-directed RNA polymerase complex |
| GO:0060147 | regulation of post-transcriptional gene silencing |
| GO:0060148 | positive regulation of post-transcriptional gene silencing |
| GO:0060149 | negative regulation of post-transcriptional gene silencing |
| GO:0060194 | regulation of antisense RNA transcription |
| GO:0060195 | negative regulation of antisense RNA transcription |
| GO:0060196 | positive regulation of antisense RNA transcription |
| GO:0060260 | regulation of transcription initiation by RNA polymerase II |
| GO:0060261 | positive regulation of transcription initiation by RNA polymerase II |
| GO:0060566 | positive regulation of termination of DNA-templated transcription |
| GO:0060567 | negative regulation of termination of DNA-templated transcription |
| GO:0060633 | negative regulation of transcription initiation by RNA polymerase II |
| GO:0060815 | regulation of translation involved in anterior/posterior axis specification |
| GO:0060962 | regulation of ribosomal protein gene transcription by RNA polymerase II |
| GO:0060963 | positive regulation of ribosomal protein gene transcription by RNA polymerase II |
| GO:0061413 | regulation of transcription from RNA polymerase II promoter by a nonfermentable carbon source |
| GO:0061414 | positive regulation of transcription from RNA polymerase II promoter by a nonfermentable carbon source |
| GO:0061415 | negative regulation of transcription from RNA polymerase II promoter by a nonfermentable carbon source |
| GO:0061425 | positive regulation of ethanol catabolic process by positive regulation of transcription from RNA polymerase II promoter |
| GO:0061429 | positive regulation of transcription from RNA polymerase II promoter by oleic acid |
| GO:0061435 | positive regulation of transcription from a mobile element promoter |
| GO:0061586 | positive regulation of transcription by transcription factor localization |
| GO:0061614 | miRNA transcription |
| GO:0061629 | RNA polymerase II-specific DNA-binding transcription factor binding |
| GO:0061770 | translation elongation factor binding |
| GO:0061986 | negative regulation of transcription by glucose |
| GO:0061987 | negative regulation of transcription from RNA polymerase II promoter by glucose |
| GO:0062058 | transcription factor TFIIH holo complex binding |
| GO:0062125 | regulation of mitochondrial gene expression |
| GO:0070054 | mRNA splicing, via endonucleolytic cleavage and ligation |
| GO:0070063 | RNA polymerase binding |
| GO:0070124 | mitochondrial translational initiation |
| GO:0070125 | mitochondrial translational elongation |
| GO:0070126 | mitochondrial translational termination |
| GO:0070127 | tRNA aminoacylation for mitochondrial protein translation |
| GO:0070129 | regulation of mitochondrial translation |
| GO:0070130 | negative regulation of mitochondrial translation |
| GO:0070131 | positive regulation of mitochondrial translation |
| GO:0070132 | regulation of mitochondrial translational initiation |
| GO:0070133 | negative regulation of mitochondrial translational initiation |
| GO:0070134 | positive regulation of mitochondrial translational initiation |
| GO:0070196 | eukaryotic translation initiation factor 3 complex assembly |
| GO:0070217 | transcription factor TFIIIB complex assembly |
| GO:0070264 | transcription factor TFIIIE complex |
| GO:0070390 | transcription export complex 2 |
| GO:0070549 | siRNA-mediated gene silencing by inhibition of translation |
| GO:0070556 | TAF4B-containing transcription factor TFIID complex |
| GO:0070860 | RNA polymerase I core factor complex |
| GO:0070897 | transcription preinitiation complex assembly |
| GO:0070898 | RNA polymerase III preinitiation complex assembly |
| GO:0070918 | regulatory ncRNA processing |
| GO:0070920 | regulation of regulatory ncRNA processing |
| GO:0070921 | regulation of siRNA processing |
| GO:0070929 | trans-translation |
| GO:0070930 | trans-translation-dependent protein tagging |
| GO:0070985 | transcription factor TFIIK complex |
| GO:0070992 | translation initiation complex |
| GO:0070993 | translation preinitiation complex |
| GO:0071004 | U2-type prespliceosome |
| GO:0071005 | U2-type precatalytic spliceosome |
| GO:0071006 | U2-type catalytic step 1 spliceosome |
| GO:0071007 | U2-type catalytic step 2 spliceosome |
| GO:0071008 | U2-type post-mRNA release spliceosomal complex |
| GO:0071010 | prespliceosome |
| GO:0071011 | precatalytic spliceosome |
| GO:0071012 | catalytic step 1 spliceosome |
| GO:0071013 | catalytic step 2 spliceosome |
| GO:0071014 | post-mRNA release spliceosomal complex |
| GO:0071015 | U12-type prespliceosome |
| GO:0071016 | U12-type precatalytic spliceosome |
| GO:0071017 | U12-type catalytic step 1 spliceosome |
| GO:0071018 | U12-type catalytic step 2 spliceosome |
| GO:0071019 | U12-type post-mRNA release spliceosomal complex |
| GO:0071020 | post-spliceosomal complex |
| GO:0071021 | U2-type post-spliceosomal complex |
| GO:0071022 | U12-type post-spliceosomal complex |
| GO:0071023 | trans spliceosomal complex |
| GO:0071030 | nuclear mRNA surveillance of spliceosomal pre-mRNA splicing |
| GO:0071262 | regulation of translational initiation in response to starvation |
| GO:0071263 | negative regulation of translational initiation in response to starvation |
| GO:0071264 | positive regulation of translational initiation in response to starvation |
| GO:0071540 | eukaryotic translation initiation factor 3 complex, eIF3e |
| GO:0071541 | eukaryotic translation initiation factor 3 complex, eIF3m |
| GO:0071891 | N-terminal peptidyl-proline dimethylation involved in translation |
| GO:0072669 | tRNA-splicing ligase complex |
| GO:0072742 | SAGA complex localization to transcription regulatory region |
| GO:0075522 | IRES-dependent viral translational initiation |
| GO:0075523 | viral translational frameshifting |
| GO:0075525 | viral translational termination-reinitiation |
| GO:0080149 | sucrose induced translational repression |
| GO:0090079 | translation regulator activity, nucleic acid binding |
| GO:0090262 | regulation of transcription-coupled nucleotide-excision repair |
| GO:0090293 | nitrogen catabolite regulation of transcription |
| GO:0090294 | nitrogen catabolite activation of transcription |
| GO:0090295 | nitrogen catabolite repression of transcription |
| GO:0090570 | RNA polymerase I transcription repressor complex |
| GO:0090571 | RNA polymerase II transcription repressor complex |
| GO:0090572 | RNA polymerase III transcription repressor complex |
| GO:0090573 | RNA polymerase IV transcription repressor complex |
| GO:0090574 | RNA polymerase V transcription repressor complex |
| GO:0090575 | RNA polymerase II transcription regulator complex |
| GO:0090576 | RNA polymerase III transcription regulator complex |
| GO:0090577 | RNA polymerase IV transcription regulator complex |
| GO:0090578 | RNA polymerase V transcription regulator complex |
| GO:0090615 | mitochondrial mRNA processing |
| GO:0090646 | mitochondrial tRNA processing |
| GO:0097010 | eukaryotic translation initiation factor 4F complex assembly |
| GO:0097167 | circadian regulation of translation |
| GO:0097221 | M/G1 phase-specific MADS box-forkhead transcription factor complex |
| GO:0097393 | telomeric repeat-containing RNA transcription |
| GO:0097523 | transcription ternary complex |
| GO:0097525 | spliceosomal snRNP complex |
| GO:0097526 | spliceosomal tri-snRNP complex |
| GO:0097550 | transcription preinitiation complex |
| GO:0097622 | cytoplasmic translational elongation through polyproline stretches |
| GO:0097747 | RNA polymerase activity |
| GO:0097748 | 3'-5' RNA polymerase activity |
| GO:0098531 | ligand-activated transcription factor activity |
| GO:0098781 | ncRNA transcription |
| GO:0098790 | ncRNA transcription associated with protein coding gene TSS/TES |
| GO:0099122 | RNA polymerase II C-terminal domain binding |
| GO:0099547 | regulation of translation at synapse, modulating synaptic transmission |
| GO:0099577 | regulation of translation at presynapse, modulating synaptic transmission |
| GO:0099578 | regulation of translation at postsynapse, modulating synaptic transmission |
| GO:0106074 | aminoacyl-tRNA metabolism involved in translational fidelity |
| GO:0106250 | DNA-binding transcription repressor activity, RNA polymerase III-specific |
| GO:0106391 | bI4 intron splicing complex |
| GO:0106392 | bI3 intron splicing complex |
| GO:0106400 | double-strand break repair via transcription-associated homologous recombination |
| GO:0110017 | cap-independent translational initiation of linear mRNA |
| GO:0110018 | cap-independent translational initiation of circular RNA |
| GO:0110019 | IRES-dependent translational initiation of circular RNA |
| GO:0110103 | RNA polymerase II termination complex |
| GO:0120185 | MBF transcription complex assembly |
| GO:0120191 | negative regulation of termination of RNA polymerase II transcription |
| GO:0120235 | regulation of post-translational protein targeting to membrane, translocation |
| GO:0120236 | negative regulation of post-translational protein targeting to membrane, translocation |
| GO:0140018 | regulation of cytoplasmic translational fidelity |
| GO:0140040 | mitochondrial polycistronic RNA processing |
| GO:0140053 | mitochondrial gene expression |
| GO:0140110 | transcription regulator activity |
| GO:0140223 | general transcription initiation factor activity |
| GO:0140236 | translation at presynapse |
| GO:0140237 | translation at presynapse, modulating chemical synaptic transmission |
| GO:0140241 | translation at synapse |
| GO:0140242 | translation at postsynapse |
| GO:0140243 | regulation of translation at synapse |
| GO:0140244 | regulation of translation at presynapse |
| GO:0140245 | regulation of translation at postsynapse |
| GO:0140296 | general transcription initiation factor binding |
| GO:0140297 | DNA-binding transcription factor binding |
| GO:0140416 | transcription regulator inhibitor activity |
| GO:0140537 | transcription regulator activator activity |
| GO:0140541 | piRNA transcription |
| GO:0140542 | regulation of piRNA transcription |
| GO:0140543 | positive regulation of piRNA transcription |
| GO:0140646 | negative regulation of pre-B cell receptor expression |
| GO:0140673 | transcription elongation-coupled chromatin remodeling |
| GO:0140742 | lncRNA transcription |
| GO:0140743 | regulation of lncRNA transcription |
| GO:0140744 | negative regulation of lncRNA transcription |
| GO:0140745 | siRNA transcription |
| GO:0140747 | regulation of ncRNA transcription |
| GO:0140764 | small RNA binding translational repressor activity |
| GO:0140766 | siRNA-mediated post-transcriptional gene silencing |
| GO:0140833 | RNA polymerase II CTD heptapeptide repeat Y1 kinase activity |
| GO:0140834 | RNA polymerase II CTD heptapeptide repeat S2 kinase activity |
| GO:0140835 | RNA polymerase II CTD heptapeptide repeat T4 kinase activity |
| GO:0140836 | RNA polymerase II CTD heptapeptide repeat S5 kinase activity |
| GO:0140837 | RNA polymerase II CTD heptapeptide repeat S7 kinase activity |
| GO:0140838 | RNA polymerase II CTD heptapeptide repeat peptidyl-prolyl isomerase activity |
| GO:0140839 | RNA polymerase II CTD heptapeptide repeat P3 isomerase activity |
| GO:0140840 | RNA polymerase II CTD heptapeptide repeat P6 isomerase activity |
| GO:0140841 | RNA polymerase II C-terminal domain O-GlcNAc transferase activity |
| GO:0140842 | RNA polymerase II C-terminal domain S5 O-GlcNAc transferase activity |
| GO:0140843 | RNA polymerase II C-terminal domain S7 O-GlcNAc transferase activity |
| GO:0140845 | regulation of promoter clearance from RNA polymerase II promoter |
| GO:0140846 | positive regulation of promoter clearance from RNA polymerase II promoter |
| GO:0140847 | negative regulation of promoter clearance from RNA polymerase II promoter |
| GO:0140870 | RNA polymerase inhibitor activity |
| GO:0140871 | repressor of RNA polymerase inhibitor activity |
| GO:0140891 | tRNA-derived regulatory ncRNA processing |
| GO:0140899 | plastid gene expression |
| GO:0140965 | secondary piRNA processing |
| GO:0140990 | primary piRNA processing |
| GO:0140994 | RNA polymerase II CTD heptapeptide repeat modifying activity |
| GO:0141096 | ligand-activated transcription repressor activity |
| GO:0141097 | ligand-activated transcription activator activity |
| GO:0141137 | positive regulation of gene expression, epigenetic |
| GO:0160021 | maternal-to-zygotic transition of gene expression |
| GO:0160091 | spliceosome-depend formation of circular RNA |
| GO:0180004 | RNA polymerase II CTD heptapeptide repeat Y1 phosphatase activity |
| GO:0180005 | RNA polymerase II CTD heptapeptide repeat T4 phosphatase activity |
| GO:0180006 | RNA polymerase II CTD heptapeptide repeat S2 phosphatase activity |
| GO:0180007 | RNA polymerase II CTD heptapeptide repeat S5 phosphatase activity |
| GO:0180008 | RNA polymerase II CTD heptapeptide repeat S7 phosphatase activity |
| GO:0180010 | co-transcriptional mRNA 3'-end processing, cleavage and polyadenylation pathway |
| GO:0180012 | co-transcriptional RNA 3'-end processing, cleavage and polyadenylation pathway |
| GO:1900247 | regulation of cytoplasmic translational elongation |
| GO:1900248 | negative regulation of cytoplasmic translational elongation |
| GO:1900249 | positive regulation of cytoplasmic translational elongation |
| GO:1900259 | regulation of RNA-dependent RNA polymerase activity |
| GO:1900260 | negative regulation of RNA-dependent RNA polymerase activity |
| GO:1900261 | positive regulation of RNA-dependent RNA polymerase activity |
| GO:1900268 | regulation of reverse transcription |
| GO:1900269 | negative regulation of reverse transcription |
| GO:1900270 | positive regulation of reverse transcription |
| GO:1900368 | regulation of post-transcriptional gene silencing by regulatory ncRNA |
| GO:1900369 | negative regulation of post-transcriptional gene silencing by regulatory ncRNA |
| GO:1900370 | positive regulation of post-transcriptional gene silencing by RNA |
| GO:1900461 | positive regulation of pseudohyphal growth by positive regulation of transcription from RNA polymerase II promoter |
| GO:1901148 | gene expression involved in extracellular matrix organization |
| GO:1901190 | regulation of formation of translation initiation ternary complex |
| GO:1901191 | negative regulation of formation of translation initiation ternary complex |
| GO:1901192 | positive regulation of formation of translation initiation ternary complex |
| GO:1901193 | regulation of formation of translation preinitiation complex |
| GO:1901194 | negative regulation of formation of translation preinitiation complex |
| GO:1901195 | positive regulation of formation of translation preinitiation complex |
| GO:1901259 | chloroplast rRNA processing |
| GO:1901836 | regulation of transcription of nucleolar large rRNA by RNA polymerase I |
| GO:1901837 | negative regulation of transcription of nucleolar large rRNA by RNA polymerase I |
| GO:1901838 | positive regulation of transcription of nucleolar large rRNA by RNA polymerase I |
| GO:1901839 | regulation of RNA polymerase I regulatory region sequence-specific DNA binding |
| GO:1901840 | negative regulation of RNA polymerase I regulatory region sequence-specific DNA binding |
| GO:1901873 | regulation of post-translational protein modification |
| GO:1901874 | negative regulation of post-translational protein modification |
| GO:1901875 | positive regulation of post-translational protein modification |
| GO:1902010 | negative regulation of translation in response to endoplasmic reticulum stress |
| GO:1902162 | regulation of DNA damage response, signal transduction by p53 class mediator resulting in transcription of p21 class mediator |
| GO:1902163 | negative regulation of DNA damage response, signal transduction by p53 class mediator resulting in transcription of p21 class mediator |
| GO:1902164 | positive regulation of DNA damage response, signal transduction by p53 class mediator resulting in transcription of p21 class mediator |
| GO:1902796 | regulation of snoRNA processing |
| GO:1902797 | negative regulation of snoRNA processing |
| GO:1902798 | positive regulation of snoRNA processing |
| GO:1902893 | regulation of miRNA transcription |
| GO:1902894 | negative regulation of miRNA transcription |
| GO:1902895 | positive regulation of miRNA transcription |
| GO:1903025 | regulation of RNA polymerase II regulatory region sequence-specific DNA binding |
| GO:1903026 | negative regulation of RNA polymerase II regulatory region sequence-specific DNA binding |
| GO:1903108 | regulation of mitochondrial transcription |
| GO:1903109 | positive regulation of mitochondrial transcription |
| GO:1903231 | mRNA base-pairing translational repressor activity |
| GO:1903241 | U2-type prespliceosome assembly |
| GO:1903270 | regulation of cytoplasmic translational elongation through polyproline stretches |
| GO:1903271 | negative regulation of cytoplasmic translational elongation through polyproline stretches |
| GO:1903272 | positive regulation of cytoplasmic translational elongation through polyproline stretches |
| GO:1903357 | regulation of transcription initiation by RNA polymerase I |
| GO:1903502 | translation repressor complex |
| GO:1903622 | regulation of RNA polymerase III activity |
| GO:1903623 | negative regulation of RNA polymerase III activity |
| GO:1903674 | regulation of cap-dependent translational initiation |
| GO:1903675 | negative regulation of cap-dependent translational initiation |
| GO:1903676 | positive regulation of cap-dependent translational initiation |
| GO:1903677 | regulation of cap-independent translational initiation |
| GO:1903678 | negative regulation of cap-independent translational initiation |
| GO:1903679 | positive regulation of cap-independent translational initiation |
| GO:1903704 | negative regulation of siRNA processing |
| GO:1903705 | positive regulation of siRNA processing |
| GO:1903798 | regulation of miRNA processing |
| GO:1903799 | negative regulation of miRNA processing |
| GO:1903800 | positive regulation of miRNA processing |
| GO:1904279 | regulation of transcription by RNA polymerase V |
| GO:1904280 | negative regulation of transcription by RNA polymerase V |
| GO:1904281 | positive regulation of transcription by RNA polymerase V |
| GO:1904388 | negative regulation of ncRNA transcription associated with protein coding gene TSS/TES |
| GO:1904594 | regulation of termination of RNA polymerase II transcription |
| GO:1904595 | positive regulation of termination of RNA polymerase II transcription |
| GO:1904688 | regulation of cytoplasmic translational initiation |
| GO:1904689 | negative regulation of cytoplasmic translational initiation |
| GO:1904690 | positive regulation of cytoplasmic translational initiation |
| GO:1904803 | regulation of translation involved in cellular response to UV |
| GO:1904971 | regulation of viral translation |
| GO:1904972 | negative regulation of viral translation |
| GO:1904973 | positive regulation of viral translation |
| GO:1905082 | regulation of mitochondrial translational elongation |
| GO:1905083 | negative regulation of mitochondrial translational elongation |
| GO:1905084 | positive regulation of mitochondrial translational elongation |
| GO:1905143 | eukaryotic translation initiation factor 2 complex assembly |
| GO:1905173 | eukaryotic translation initiation factor 2B complex assembly |
| GO:1905255 | regulation of RNA binding transcription factor activity |
| GO:1905256 | negative regulation of RNA binding transcription factor activity |
| GO:1905257 | positive regulation of RNA binding transcription factor activity |
| GO:1905380 | regulation of snRNA transcription by RNA polymerase II |
| GO:1905381 | negative regulation of snRNA transcription by RNA polymerase II |
| GO:1905382 | positive regulation of snRNA transcription by RNA polymerase II |
| GO:1905535 | regulation of eukaryotic translation initiation factor 4F complex assembly |
| GO:1905536 | negative regulation of eukaryotic translation initiation factor 4F complex assembly |
| GO:1905537 | positive regulation of eukaryotic translation initiation factor 4F complex assembly |
| GO:1905636 | positive regulation of RNA polymerase II regulatory region sequence-specific DNA binding |
| GO:1905744 | regulation of mRNA cis splicing, via spliceosome |
| GO:1905745 | negative regulation of mRNA cis splicing, via spliceosome |
| GO:1905746 | positive regulation of mRNA cis splicing, via spliceosome |
| GO:1990113 | RNA polymerase I assembly |
| GO:1990114 | RNA polymerase II core complex assembly |
| GO:1990115 | RNA polymerase III assembly |
| GO:1990145 | maintenance of translational fidelity |
| GO:1990216 | symbiont-mediated activation of of host transcription |
| GO:1990250 | transcription-coupled nucleotide-excision repair, DNA damage recognition complex |
| GO:1990269 | RNA polymerase II C-terminal domain phosphoserine binding |
| GO:1990433 | CSL-Notch-Mastermind transcription factor complex |
| GO:1990497 | regulation of cytoplasmic translation in response to stress |
| GO:1990513 | CLOCK-BMAL transcription complex |
| GO:1990580 | regulation of cytoplasmic translational termination |
| GO:1990589 | ATF4-CREB1 transcription factor complex |
| GO:1990590 | ATF1-ATF4 transcription factor complex |
| GO:1990611 | regulation of cytoplasmic translational initiation in response to stress |
| GO:1990625 | negative regulation of cytoplasmic translational initiation in response to stress |
| GO:1990787 | negative regulation of hh target transcription factor activity |
| GO:1990817 | poly(A) RNA polymerase activity |
| GO:1990935 | splicing factor binding |
| GO:1990969 | modulation by host of viral RNA-binding transcription factor activity |
| GO:1990983 | regulation of translational initiation by tRNA modification |
| GO:2000142 | regulation of DNA-templated transcription initiation |
| GO:2000143 | negative regulation of DNA-templated transcription initiation |
| GO:2000144 | positive regulation of DNA-templated transcription initiation |
| GO:2000232 | regulation of rRNA processing |
| GO:2000233 | negative regulation of rRNA processing |
| GO:2000234 | positive regulation of rRNA processing |
| GO:2000235 | regulation of tRNA processing |
| GO:2000236 | negative regulation of tRNA processing |
| GO:2000237 | positive regulation of tRNA processing |
| GO:2000631 | regulation of pre-miRNA processing |
| GO:2000632 | negative regulation of pre-miRNA processing |
| GO:2000633 | positive regulation of pre-miRNA processing |
| GO:2000634 | regulation of primary miRNA processing |
| GO:2000635 | negative regulation of primary miRNA processing |
| GO:2000636 | positive regulation of primary miRNA processing |
| GO:2000677 | regulation of transcription regulatory region DNA binding |
| GO:2000678 | negative regulation of transcription regulatory region DNA binding |
| GO:2000679 | positive regulation of transcription regulatory region DNA binding |
| GO:2000730 | regulation of termination of RNA polymerase I transcription |
| GO:2000731 | negative regulation of termination of RNA polymerase I transcription |
| GO:2000732 | positive regulation of termination of RNA polymerase I transcription |
| GO:2000765 | regulation of cytoplasmic translation |
| GO:2000766 | negative regulation of cytoplasmic translation |
| GO:2000767 | positive regulation of cytoplasmic translation |
| GO:2000804 | regulation of termination of RNA polymerase II transcription, poly(A)-coupled |
| GO:2000805 | negative regulation of termination of RNA polymerase II transcription, poly(A)-coupled |
| GO:2000806 | positive regulation of termination of RNA polymerase II transcription, poly(A)-coupled |
| GO:2001124 | regulation of translational frameshifting |
| GO:2001125 | negative regulation of translational frameshifting |
| GO:2001126 | positive regulation of translational frameshifting |
| GO:2001207 | regulation of transcription elongation by RNA polymerase I |
| GO:2001208 | negative regulation of transcription elongation by RNA polymerase I |
| GO:2001209 | positive regulation of transcription elongation by RNA polymerase I |
